# A trimetallic bismuth(I)-based allyl cation

**DOI:** 10.1038/s41557-024-01691-x

**Published:** 2025-01-06

**Authors:** Davide Spinnato, Nils Nöthling, Markus Leutzsch, Maurice van Gastel, Lucas Wagner, Frank Neese, Josep Cornella

**Affiliations:** Max-Planck-Institut für Kohlenforschung, Mülheim an der Ruhr, Germany

**Keywords:** Chemical bonding, Chemical bonding

## Abstract

The chemistry of low-valent bismuth compounds has recently unlocked new concepts in catalysis and unique electronic structure fundamentals. In this work, we describe the synthesis and characterization of a highly reduced bismuth salt featuring a cationic core based on three contiguous Bi(I) centres. The triatomic bismuth-based core exhibits an electronic configuration that mimics the canonical description of the archetypical carbon-based *π*-allyl cation. Structural, spectroscopic and theoretical analyses validate the unique *π*-delocalization between the bismuth’s highly diffused 6*p* orbitals, resulting in a bonding situation in which the three bismuth atoms are interconnected by two bonds, formally possessing a 1.5 bond order each. This electronic situation defines this complex as the heaviest and stable *π*-allyl cation of the periodic table. Furthermore, we demonstrate that the newly synthesized complex is able to act as a synthon for the transfer of a Bi(I) cation to forge other low-valent organobismuth complexes.

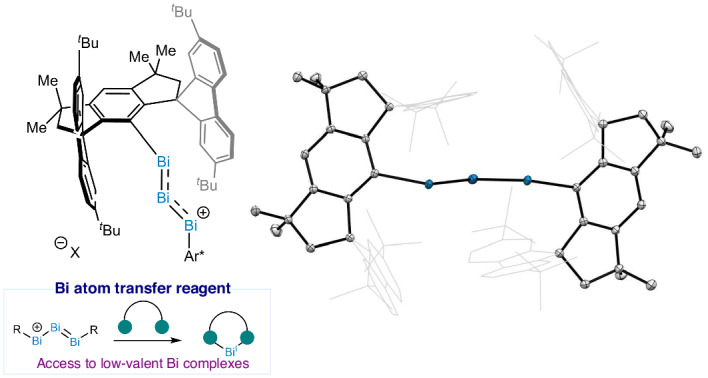

## Abstract (web summary)

Heavier analogues of unsaturated organic molecules are of interest because of their bonding situation and their potential use in synthesis. Now, a Bi(I)-based allyl cation, which can be seen as a heavy congener of all-carbon *π*-allyl cations, has been reported. This complex serves as a synthon for Bi(I) transfer, enabling access to low-valent organobismuth compounds.

## Main

**Fig. 1:**
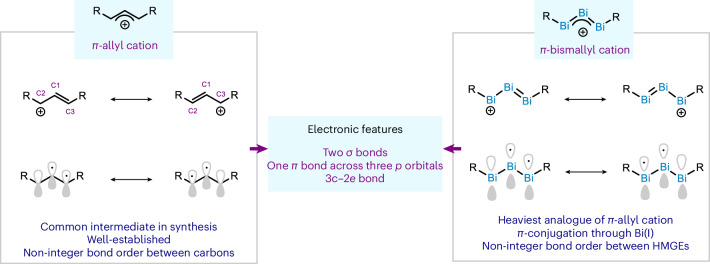
The allyl cation system. Carbon-based *π*-allyl cation (left) and its heaviest stable analogue based on bismuth (right).

The *π*-allyl cation is one of the most recognizable intermediates in organic chemistry, and represents a textbook example to explain molecular orbital theory, resonance and chemical bonding^1–7^. The unique allylic system of the triatomic carbon arrangement has been the object of study of numerous research groups and has served as guiding principle to develop a plethora of synthetic transformations^8–12^. From the electronic point of view, the *π*-allyl cation is comprised of three carbon atoms (C1, C2 and C3; Fig. 1, left), with C1 connected to C2 and C3 in a 1.5 bond order. More specifically, these carbons are *sp*^2^ hybridized and are united through two *σ* bonds. In addition, the three available 2*p* orbitals allow the delocalization of the two additional electrons, thus conforming the characteristic delocalized *π*-system^13,14^. Fascinated by its structure, chemists have looked at synthesizing heavier analogues of the *π*-allyl cation; yet, the analogues reported are cyclopropenium-type structures^15–17^ or, in some cases, vinylogous systems which capitalize on the delocalization of the positive charge into neighbouring heteroatoms^18,19^. Hence, a genuine *π*-allyl cation analogue based on heavier elements remains elusive. Due to its privileged place in the periodic table as the last stable element, the heaviest analogue of this cationic structure should be within reach using bismuth. A direct comparison of the frontier orbitals involved in bonding leads to the realization that a cationic triatomic complex of bismuth with formal oxidation state +1 for the three contiguous bismuth atoms (Bi)_3_^+^ would be analogous to that of the carbon-based allyl cation (Fig. 1, right). This seemingly simple analogy presents an enormous synthetic challenge: examples of unsupported neutral or cationic dicoordinated Bi(I) compounds are rare^20^. Siddiqui et al. developed a cationic Bi(I) supported by two sterically encumbered cyclic alkyl(amino) carbene^21^, and Zhao and Mo reported a bis(silylene)-stabilized Bi(I) cation^22^. Recently, the group of Driess and Frenking developed a similar structure with germylenes as supporting ligands^23^. For many years, overlapping of large and diffused orbitals leading to chemical bonding between 5*p* and 6*p* orbitals was regarded as a challenge in organometallic chemistry^24,25^. However, this canonical notion has been challenged with several examples of heavy main-group elements (HMGEs) that form double and triple bonds, or all-metal *φ*- and *σ*-aromatic structures akin to the lightest main group elements^24–33^. Based on our recent findings on a monocoordinated bismuthinidene **1** (ref. ^34^), here we present the synthesis, structure, characterization and reactivity of a unique cationic complex featuring three contiguously bonded bismuth atoms in a low oxidation state. The electronic structure calculations reveal the delocalization of two electrons through the non-hybridized 6*p* orbitals of the trimetallic (Bi_3_)^+^ core—due to the low energy of the buried 6*s*^2^ orbitals—characteristic of the classical *π*-allyl cation.

## Results and discussion

### Synthesis and structure determination

**Fig. 2:**
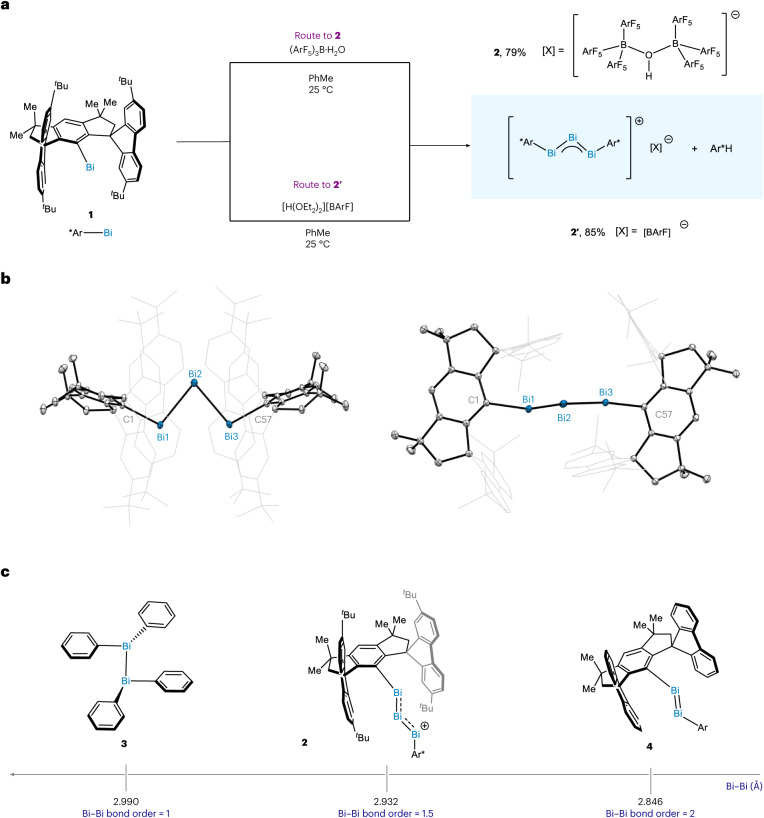
Preparation of a trimetallic Bi(I)-based allyl cation and structure analysis. **a**, Synthesis of **2** and **2′**. **b**, Solid-state structure of **2** at 100 K in two orientations (anisotropic displacement ellipsoids are displayed at a probability level of 50%; hydrogen atoms, anions and solvent molecules are omitted for clarity). **c**, Comparison of Bi‒Bi bond length and formal bond orders.

When **1** was mixed with monohydrated tris(pentafluorophenyl)borane [(ArF_5_)_3_B·H_2_O] in PhMe-*d*_8_ at 25 °C, **2** precipitated from the mixture as an air-sensitive, dark-brown solid. The protonated organic framework was obtained as co-product (Fig. 2a). Similarly, when **1** is mixed with Brookhardt’s acid [(Et_2_O)_2_H]·[BArF] (BArF = tetrakis[3,5-bis(trifluoromethyl)phenyl]borate) in PhMe at 25 °C, the same cationic moiety is obtained, with BArF as the counterion (**2′**). The structure of **2** and **2′** were unambiguously determined by single-crystal X-ray diffraction (SC-XRD) which revealed their ionic nature. In both compounds, the cation is constituted by a triatomic bismuth core connected in a linear zigzag fashion with the bulky organic backbone; whereas [(ArF_5_)_3_B]_2_OH serves as counteranion in **2**, BArF is the counteranion in **2′**. In **2**, the two distances between Bi1–Bi2 and Bi2–Bi3 (2.93428(12) Å and 2.93294(11) Å, respectively) are 2% shorter (∆*d* = 0.0571 Å) than the Bi–Bi single bond (2.990(2) Å) in **3** (Fig. 2c)^35^, and 3% longer (∆*d* = 0.08721 Å) than the Bi=Bi double-bond distance in dibismuthene **4** (2.8464(4) Å)^34^. Together with previously described bond lengths^27,34–37^, the distances in **2** fall in the range between a single and a double bond. At the same time, the Bi1−C1 and Bi3-C57 distances in **2** suggests a single bond (2.2833(16) Å and 2.2869(16) Å in **2** versus 2.2783(10) Å in **1**) (see below)^20,34,37,38^. The observed C1‒Bi1‒Bi2, C57–Bi2–Bi3 and Bi1‒Bi2‒Bi3 bond angles (101.33(4)°, 101.24(4)° and 80.637(3)°, respectively) are indicative of the limited ability of bismuth to form hybridized *sp*^2^ orbitals, primarily due to the contraction of the 6*s* orbital as a consequence of relativistic effects^27,39,40^. In solution, compound **2** is stable in dicholoromethane-*d*_2_ and could be fully characterized at 25 °C by NMR. At this temperature, **2** exhibits a dynamic exchange behaviour, leading to a pseudosymmetric NMR dataset; however, this character is lost at −100 °C, in line with the crystal structure which possesses a *P-*1 space group (Supplementary Fig. 14). A single set of C_(Ar)_–F signals can also be observed at 25 °C, arising from the [(ArF_5_)_3_B]_2_OH anion. At lower temperatures, this equivalence is broken and three distinct C_(Ar)_–F groups appear, in agreement with previous observations for this anion^41^. The structural features and solution behaviour of **2′** resemble those of **2**. Compounds **2** and **2′** appear to be diamagnetic, and no signal was observed by EPR.

### Theoretical and spectroscopic analysis

**Fig. 3:**
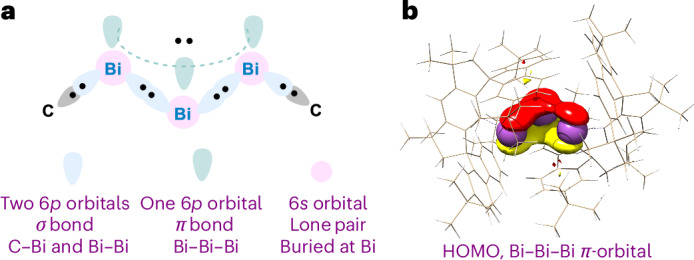
Valence electronic structure of 2. **a**, Lewis structure representing the (Bi)_3_^+^ core and the electrons involved in bonding. **b**, Calculated highest occupied molecular orbital (HOMO) containing the delocalized electron pair (purple, 6*s* orbitals; red, *π* orbital (above nodal plane); yellow, *π* orbital (below nodal plane).

On the basis of the crystal structures, the geometric structure of the cationic moiety in **2** was optimized without constraints using the ORCA 5.0^42^ program suite together with the B3LYP functional^43^, the D3(BJ) dispersion correction^44^ and the def2-TZVP basis set with associated effective core potentials^45^. The obtained stationary point was confirmed to be a minimum through the calculation of harmonics frequencies. The calculated Bi1/3–Bi2 distance of 2.912 Å and the Bi1/3‒C_Ar_ distance of 2.295 Å (average) are in overall agreement with the SC-XRD data of 2.93361(12) Å and 2.2851(16) Å, respectively. Given the strong sensitivity of the Bi‒Bi distance to the bond order between the two atoms, these results provide evidence for the notion that the electronic situation in the (Bi)_3_^+^ core is properly represented by the calculations. A closer analysis of the electronic structure of the cationic core in **2** confirms that the two Bi1/3 are nearly equivalent and distinct from the bridging Bi2. As deduced from the analysis of the occupied orbitals, all bismuth atoms feature a non-bonding lone pair of electrons corresponding to the inert 6*s*^2^ (Fig. 3a). At the same time, the terminal bismuth atoms further engage in two-electron–two centre single bonds with the ligand framework as well as with Bi2 respectively. The central Bi2 atom forges single bonds with the terminal atoms instead. The remaining two valence electrons are delocalized over the three centres in the core. The Mayer bond order^46^ for the Bi1‒Bi2 and Bi2‒Bi3 bonds is equal to 1.4, consistent with a bonding situation in which each Bi‒Bi pair is interconnected by one bond, each formally possessing a 1.5 bond order. In contrast, a value of 0.91 was found for the Bi‒C_Ar_ bond, as expected for single bonds. The calculated Mayer bond order for the Bi1‒Bi3 is only 0.28, which is indicative of a very weak interaction between these two centres. In terms of charge delocalization, the outer Bi1/3 centres carry slightly more positive charge (natural population analysis (NPA) charge, ∼+0.7) than the bridging Bi2 (NPA charge, ∼+0.15). These charges add up to more than one full positive charge due to the charge transfer of the Bi1/3 centres to the C1/57 which possess a negative charge of ∼−0.34 each.

**Fig. 4:**
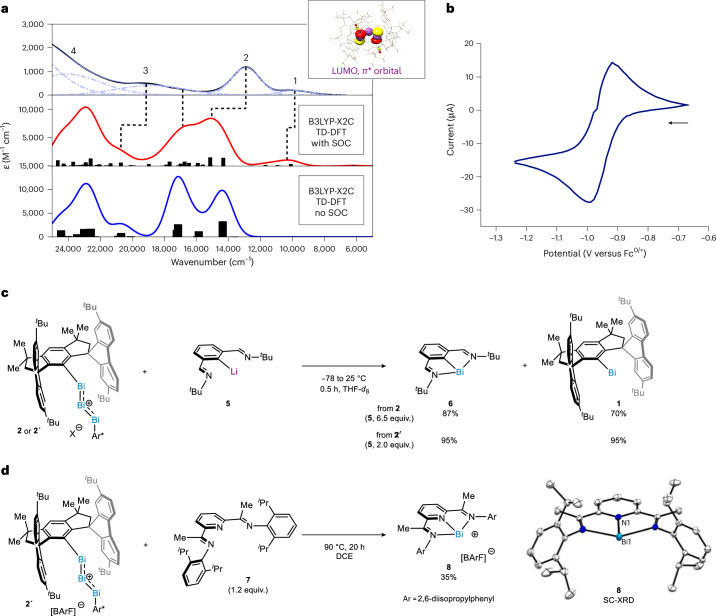
Spectroscopic, electrochemical and reactivity studies. **a**, Experimental UV–Vis spectrum resolved into individual Gaussian absorption bands labelled 1–4 and shown in light purple, and calculated UV–Vis absorption spectra: red profile, including SOC; blue profile, without SOC (left); *ε*, molar extinction coefficient. Calculated lowest unoccupied molecular orbital (LUMO) corresponding to the π* orbital (top right). **b**, Cyclic voltammogram of **2** in DCM using 0.1 M [nBu_4_N][BArF] as supporting electrolyte at ambient temperature. Scan rate, 100 mV s^−1^, referenced to the ferrocenium redox couple (Fc^0/**+**^). The arrow indicates the direction of the potential sweep. **c**, Reaction of **2** or **2′** as transfer reagent of Bi(I) cations. Yield and distribution of products **6** and **1** were determined by ^1^H NMR analysis of the crude mixture using 1,3,5-trimethoxybenzene as the internal standard. **d**, Reaction of **2′** as transfer reagent of Bi(I) cations towards the synthesis of a tridentate cationic Bi(I) complex **8** and its solid-state structure (SC-XRD at 100 K where atomic displacement parameters are shown at a probability level of 50%, and anion, disordered parts and solvent molecules are omitted for clarity). Yield corresponds to isolated pure material; DCE, 1,2-dichloroethane. Source data

The ultraviolet–visible absorption spectra (UV–Vis) of compounds **2** and **2′** show absorption bands at 27,701, 19,305, 12,887 and 10,000 cm^−1^ (Fig. 4a, top). This was also calculated using time-dependent density functional theory (TD-DFT). These all-electron calculations were performed with and without the inclusion of spin-orbit coupling (SOC) using the X2C-relativistic Hamiltonian^47^ and the spin-orbit mean-field operator^48^ together with finite nucleus^49,50^ and picture change corrections^51^. Singlet- and triplet excited states calculated from the scalar-relativistic TD-DFT calculations were allowed to interact via the SOC using quasi-degenerate perturbation theory. The resulting calculated spectra (Fig. 4a, middle and bottom) compare favourably with experiment if the SOC is included. In particular, the trailing intensity observed between 8,000 and 12,000 cm^−1^ results from a series of singlet-to-triplet transitions that borrow intensity via the SOC, while the main bands observed around 13,000 cm^−1^ and 19,000 cm^−1^ are dominantly singlet–singlet based. Interestingly, the calculated spectra predicts that the observed intensities in the UV–visible spectra derive from transitions that all involve the same acceptor orbital, namely the lowest unoccupied molecular orbital (LUMO) of the Bi_3_ core. The first two excited states are of (Bi)_3_ *σ* → (Bi)_3_ π* and (Bi)_3_ *π* → (Bi)_3_ π* in nature, and account for the bulk of the intensity observed experimentally at 13,000 cm^−1^. The remaining transitions all account for a complex set of ligand-to-metal charge transfer into the (Bi)_3_^+^ π* acceptor orbital.

### Reactivity

The rich optical spectrum with all transitions having the same LUMO as acceptor points to the (Bi)_3_^+^ core being electrophilic, and hence, facile single-electron transfer reduction should be within reach. Indeed, cyclic voltammetry of **2** in dichloromethane (DCM) reveals a reversible reduction wave at *E*_1/2_ = −0.95 versus Fc^0/+^, which we ascribed to the facile reduction to the corresponding fleeting *π*-allyl radical (Fig. 4b and Supplementary Figs. 19–23). Unfortunately, our attempts to isolate this paramagnetic congener of the starting complex **2** via chemical reduction have proven unsuccessful so far. The very low-lying LUMO and a highly electrophilic (Bi)_3_^+^ core led us to explore complex **2** as a potential electrophilic source of Bi(I) cations. Recently, some main-group-based complexes have been used as transfer agents to easily transfer pnictogen atoms^52,53^. When **2** was treated with a solution of organolithium reagent **5**, *N*,*C*,*N*-bismuthinidene **6** was obtained in 80% yield, with partial recovery of **1** (Fig. 4c). Such atom-transfer reactivity was also tested on the synthesis of an elusive tridentate polynitrogenated Bi(I) complex, currently inaccessible due to chemoselectivity issues arising from the forcing reducing conditions required from the parent Bi(III). Indeed, **2′** reacts with **7** at 90 °C to forge compound **8** in 35% yield as a blue crystalline solid. Complex **8** was isolated and completely characterized by NMR and high-resolution mass spectrometry (HRMS), and its connectivity confirmed by SC-XRD crystallography. The ability to shuttle Bi(I) atoms between two molecules represents a blueprint of reactivity that opens new avenues for the synthesis of low-valent bismuth-based compounds^37,54^.

## Conclusions

We have synthesized genuine analogues of the *π*-allyl cation by replacing the typical carbon-based framework with three contiguous bismuth atoms. The synthesis of these complexes demonstrates the ability of very diffuse and large frontier *p* orbitals of HMGEs to effectively overlap, similarly to their lighter congeners, thus allowing effective *π*-delocalization electrons. Intriguingly, the molecular architecture of the bismallyl cation is characterized by two Bi‒Bi bonds formally possessing a non-integer bond order. However, as expected when HMGEs are involved, the electronic description becomes more entangled due to SOC and relativistic effects. Indeed, according to our interpretation of the absorption spectrum, the intensity in the red region of the spectrum (∼700–900 nm) originates from triplet states that borrow intensity from their singlet counterparts by strong SOC mixing. This situation is highly characteristic of the heavy element bismuth which tends to break the established spin- and space-selection rules that govern the properties of most complexes bearing lighter main-group elements. In addition, due to the electrophilicity of the highly reduced (Bi)_3_^+^ core, the compound can be used to effectively transfer a Bi(I) atom, providing a blueprint of reactivity for assembling materials and molecular architectures based on low-valent bismuth compounds. Overall, this complex expands the understanding of chemical bonding in this area of the periodic table and sets novel boundaries in the chemistry of main-group elements towards conjugated systems.

## Methods

### Synthesis of 2

In an argon-filled glovebox, **1** (80 mg, 0.085 mmol, 1.5 equiv.) and monohydrated tris(pentafluorophenyl)borane (BCF) (30 mg, 0.056, 1.0 equiv.) were weighed in an oven-dried 12 mL vial, followed by the addition of 4.5 mL of anhydrous toluene. Upon stirring at 25 °C, a dark-brown precipitate is formed. The mixture is stirred for 1 h and diluted with 2.0 mL of anhydrous pentane. The heterogeneous mixture is transferred to a glass-fritted filter (porosity IV) and the solid material is washed several times with anhydrous pentane (total amount 15 mL). The dark-brown solid is dried under high vacuum to obtain **2** (70 mg, 79%). ^1^H NMR (600 MHz, DCM-*d*_2_, 298 K): *δ* (ppm) 7.35 (d, *J* = 1.8 Hz, 8H, Ar-*H*), 7.21 (d, *J* = 7.9 Hz, 8H, Ar-*H*), 7.12 (dd, *J* = 7.9, 1.8 Hz, 8H, Ar-*H*), 6.82 (s, 2H, H-1, Ar-*H*), 6.63 (bs, 1H, H-100, O-*H*), 2.11 (s, 8H, H-6, C*H*_2_), 1.50 (s, 24H, H-16, C(C*H*_3_)_2_), 1.23 (s, 72H, H-15, C(C*H*_3_)_3_). ^13^C NMR (151 MHz, DCM-*d*_2_, 298 K): *δ* (ppm) 157.6, 156.6, 156.4, 152.9, 148.0 (dm, *J* = 240.3 Hz), 146.6, 140.0, 139.8 (dm, *J* = 248.2 Hz), 137.0 (dm, *J* = 247.7 Hz), 126.2, 125.0, 123.1, 122.2, 66.6, 58.4, 44.1, 35.1, 32.2, 32.1. The signal of C-101 (119.5 ppm) was only visible in a ^13^C{^19^F} NMR spectrum. ^19^F NMR (564 MHz, DCM-*d*_2_, 298 K): *δ* (ppm) ‒133.51 (bs), ‒159.74 (t, *J* = 19.9 Hz), ‒165.79 (t, *J* = 20.2 Hz). The compound melts at 295 °C in a flame-sealed argon-filled capillary. HRMS (electrospray ionization, positive (ESI pos)): calc. for C_112_H_130_Bi_3_^+^ [M]^+^: 2101.95790; found: 2101.9602. Anal. calc. for **2**·toluene-*d*_8_ (C_162_H_131_D_16_B_2_Bi_3_F_30_O): C, 58.18; H, 4.91; D, –; B, 0.65; Bi, 18.75; F, 17.04; O, 0.48; found: C, 58.30; H, 4.09; B, 0.63; Bi, 18.58; F, 16.87. Deuterium and oxygen could not be measured.

### Synthesis of 2′

In an argon-filled glovebox, **1** (126 mg, 0.133 mmol, 3.00 equiv.) and HBArF·2Et_2_O (45.0 mg, 0.044 mmol, 1.00 equiv.) were weighed in an oven-dried 25.0 mL Schlenk tube, followed by the addition of 4.00 mL of anhydrous toluene. Upon stirring at 25 °C, a dark-brown precipitate is formed. The mixture is stirred for 15 min and diluted with 2.00 mL of anhydrous pentane. The heterogeneous mixture is transferred to a glass-fritted filter (porosity IV) and the solid material is washed several times with anhydrous pentane (total amount, 20.0 mL). The dark-brown solid is dried under high vacuum to obtain **2′** (112 mg, 0.038 mmol, 85%). ^1^H NMR (600 MHz, DCM-*d*_2_, 298 K): *δ* (ppm) 7.74–7.70 (m, 8H, Ar-*H*), 7.56 (s, 4H, Ar-*H*), 7.35 (d, *J* = 1.8 Hz, 8H, Ar-*H*), 7.22 (dd, *J* = 7.9, 0.5 Hz, 8H, Ar-*H*), 7.12 (dd, *J* = 7.9, 1.8 Hz, 8H, Ar-*H*), 6.82 (s, 2H, Ar-*H*), 2.11 (s, 8H, C*H*_2_), 1.49 (s, 24H, C(C*H*_3_)_2_), 1.23 (s, 72H, C(C*H*_3_)_3_). ^13^C NMR (151 MHz, DCM-*d*_2_, 298 K): *δ* (ppm) 162.2 (q, *J* = 49.8 Hz), 157.6, 156.6, 156.39, 152.9, 146.6, 140.0, 135.4–134.8 (m), 129.3 (qq, *J* = 31.5, 3.0 Hz), 126.2, 125.0 (q, *J* = 272.2 Hz), 124.9, 123.1, 122.2, 118.0–117.8 (m), 66.6, 58.0, 44.1, 35.2, 32.2, 32.1. ^11^B (192 MHz, DCM-*d*_2_, 298 K) *δ* (ppm) ‒6.6. ^19^F NMR (564 MHz, DCM-*d*_2_, 298 K): δ (ppm) ‒62.9. The compound melts at 310 °C in a flame-sealed argon-filled capillary. HRMS (ESI pos) calc. for C_112_H_130_Bi_3_^+^ [M]^+^: 2,101.95747; found: 2,101.95790.

### Synthesis of 8

In an argon-filled glovebox, an oven-dried Schlenk tube is charged with **2′** (15 mg, 0.0044 mmol, 1 equiv.), and **7** (2.5 mg, 0.0052 mmol, 1.2 equiv). Finally, anhydrous 1,2-dichloroethane (0.22 mL) is added and the tube is placed in an oil bath at 90 °C. After 20 h, the mixture is cooled to 25 °C and the volatiles are evaporated under high vacuum. The crude material is treated under an inert atmosphere with a 1:1 mixture of toluene/pentane (3 mL in total) and the blue liquid phase is place into a vial at –35 °C. After 10 d blue crystals could be obtained in 35% yield (2.4 mg, 0.0020 mmol) after washing the material with anhydrous pentane and drying it under high vacuum. ^1^H NMR (600 MHz, DCM-*d*_*2*_) *δ* (ppm) 9.11 (d, *J* = 7.8 Hz, 2H, Ar-*H*(pyridine)), 7.74–7.69 (m, 8H, Ar-*H*(BArF)), 7.55 (s, 4H, Ar-*H*(BArF)), 7.43–7.35 (m, 6H, Ar-*H*), 7.07 (t, *J* = 7.9 Hz, 1H, Ar-*H*(pyridine)), 3.99 (s, 6H, C*H*_3_), 2.42 (hep, *J* = 6.9 Hz, 4H, C*H*(CH_3_)_2_), 1.17 (d, *J* = 6.9 Hz, 12H, CH(C*H*_3_)_2_), 1.09 (d, *J* = 6.8 Hz, 12H, CH(C*H*_3_)_2_). ^13^C NMR (151 MHz, DCM-*d*_2_, 298 K) *δ* (ppm)168.8, 162.1 (q, *J* = 49.8 Hz), 154.8, 141.7, 138.4, 135.9, 135.6–134.5 (m), 129.5, 129.3 (qq, *J* = 31.5, 3.0 Hz), 125.0 (q, *J* = 272.4 Hz), 124.8, 118.0–117.8 (m), 29.4, 25.6, 24.0, 19.7. ^11^B (192 MHz, DCM-*d*_2_, 298 K) *δ* (ppm) −6.7. ^19^F NMR (282 MHz, DCM-*d*_2_, 298 K) −62.8. The compound melts at 280 °C in a flame-sealed argon-filled capillary. HRMS (ESI pos) calc. for C_33_H_43_Bi_1_N_3_^+^ [M]^+^: 690.32552; found: 690.32555. Anal. calc. for **8**·toluene (C_72_H_63_BBiF_24_N_3_): C, 52.54; H, 3.86; B, 0.66; Bi, 12.70; F, 27.70; N, 2.55; found: C, 52.46; H, 3.84; B, 0.67; Bi, 12.67; F, 27.64; N, 2.53.

## Online content

Any methods, additional references, Nature Portfolio reporting summaries, source data, extended data, supplementary information, acknowledgements, peer review information; details of author contributions and competing interests; and statements of data and code availability are available at 10.1038/s41557-024-01691-x.

## Supplementary information

Supplementary Information Supplementary Figs. 1–80 and Tables 1–6.

Supplementary Data 1 Crystallographic data for compound 2 (CCDC 2336523).

Supplementary Data 2 Crystallographic data for compound 2′ (CCDC 2368301).

Supplementary Data 3 Crystallographic data for compound 8 (CCDC 2368300).

Supplementary Data 4 X-ray crystal structure analysis, bond length and angles.

Supplementary Data 5 Coordinates from quantum chemical calculations on compound 2.

## Source data

Source Data Fig. 4 Source data for Fig. 4a,b.

## Data Availability

All data generated and analysed during this study are included in this Article and its Supplementary Information. Crystallographic data for the structures reported in this article have been deposited at the Cambridge Crystallographic Data Centre, under deposition numbers CCDC 2336523 (**2**), 2368301 (**2′**) and 2368300 (**8**). Source data are provided with this paper.
