# Supplementary material for: A trimetallic bismuth(I)-based allyl cation
Source: Nat Chem. 2025 Jan 6;17(2):265–70. doi: 10.1038/s41557-024-01691-x (PMC11794141; doi:10.1038/s41557-024-01691-x)
Supplement: Supplementary file 1 — Supplementary Figs. 1–80 and Tables 1–6. [file 41557_2024_1691_MOESM1_ESM.pdf]

nature chemistry

Supplementary information https://doi.org/10.1038/s41557-024-01691-x

A trimetallic bismuth(I)-based allyl cation

In the format provided by the authors and unedited

Table of contents

1. General considerations **2**

2. Synthesis of starting materials 1 and BCF • H_2O **3**

2.1. Synthesis of **1** 3
2.2. Synthesis and characterization of **BCF • H_2O** 4

3. Synthesis, Characterization and reactivity of 2 .... **6**

3.1. Synthesis and characterization of **2** 6
3.2. Synthesis and characterization of **2'** 17
3.3. Reactivity of **2** as a Bi(I) atom transfer 24
3.4. Reactivity of **2'** as a Bi(I) atom transfer: N,C,N pincer Bi(I) synthesis 25
3.5. Reactivity of **2'** as a Bi(I) atom transfer: N,N,N pincer Bi(I) synthesis 26

4. X-ray Crystal Structure Analyses **37**

4.1. Single crystal structure analysis of **2**• 2 toluene-d_8 37
4.2 Structure search in the Cambridge Structural Database (CSD) 41
4.3 Structure chemical discussions 45
4.4. Single crystal structure analysis of **2'** 51
4.5. Single crystal structure analysis of **8** 55

5. Computational Details **58**

5.1. Computational methods 58
5.2. Calculated absorption of **2** 58
5.3. Comparison of different functionals 59
5.4. Calculated orbitals for the C-Bi_3^+-C core 61

6. References **61**

1. General considerations

All experiments were conducted in flame-dried glassware under argon atmosphere by using standard Schlenk techniques or a MBraun glovebox and freshly dried and degassed solvents. THF, THF-d_8, toluene, toluene-d_8 and *n*-pentane were distilled from potassium and stored over 4 Å molecular sieves (activated at 200 °C under a high vacuum for 3 d) under argon prior to use, whereas Dichloromethane-d_2 was dried over CaH_2 and stored over 4 Å molecular sieves. Anhydrous $BiBr_3$ (97%) and cobaltocene (min. 98%) were purchased from Strem Chemicals, and tris(pentafluorophenyl)borane was purchased from TCI chemicals. Tris(pentafluorophenyl)borane and cobaltocene was stored in the freezer of the glove box prior to use. All other reagents were obtained from commercial suppliers and used without further purification.

NMR data were recorded using a Bruker AVIII HD 300 MHz, a Bruker AVIII 500 MHz or a Bruker AVNeo 600 MHz NMR spectrometer. ^{1}H and ^{13}C chemical shifts are reported relative to the solvent residual peaks as an internal reference. For ^{1}H NMR the following residual proton peaks of the deuterated solvents were used: CD_2Cl_2, $\delta_H(CHDCl_2)$ 5.32 ppm; THF-d_8, $\delta_H[(CD_2)_3CHDO]$ 1.720 ppm. For ^{13}C NMR: CD_2Cl_2, δ 53.84 ppm; THF-d_8, δ 25.31 ppm. Unless mentioned otherwise ^{13}C and ^{11}B spectra were generally acquired with broadband proton decoupling. Other nuclei (^{19}F, ^{11}B, ^{15}N) are reported relative to common standards ($CFCl_3$, $BF_3{\cdot}OEt_2$, $MeNO_3$).

ESI-MS: ESQ 3000 (Bruker). Melting points were measured with an EZ-Melt Automated Melting Point Apparatus from Standford Research Systems. UV-Vis-NIR measurements were performed at room temperature under argon using a Cary6000i spectrophotometer equipped with an InGaAs detector. IR spectra were measured by using a Thermo Scientific NicoletTM iS5 Spectrometer with an ID7 ATR accessory in a glovebox. The details of single crystal X-ray diffraction analysis, and computational studies are described in the following sections.

2. Synthesis of starting materials 1 and BCF • H_2O

2.1. Synthesis of **1**

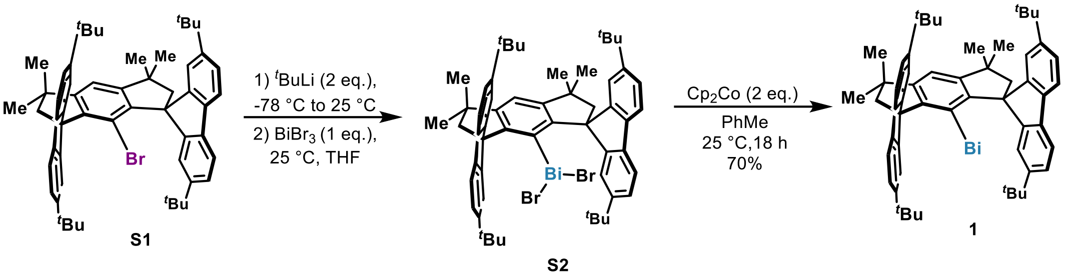

Compound **1** was synthesized in a two steps procedure starting from **S1**.[1]

Synthesis of **S2**: to a suspension of **S1** (4.00 g, 4.88 mmol, 80 mL THF) at –78 °C, tBuLi (1.7 M in pentane, 5.75 mL, 9.77 mmol, 2.0 equiv.) was added dropwise under argon. The suspension was allowed to reach to room temperature. After 90 min, the reaction mixture turned into a pale brown solution. In the glovebox, $BiBr_3$ (2.19 g, 4.88 mmol, 1.0 equiv., in 40 mL THF) was added into the reaction mixture, and the reaction mixture turned into a bright yellow suspension. After stirring at room temperature for 18 h, the reaction mixture was evaporated to dryness and further purified by column chromatography on silica gel (iso-hexane/CH_2Cl_2 = 4:1, v/v, to CH_2Cl_2), afforting 3.10 g tBu-MsFluind-$BiBr_2$ (**S2**) as a bright yellow solid in 57% yield.

^{1}H NMR (300 MHz, THF) δ 7.70 (s, 1H), 7.52 (d, *J* = 8.0 Hz, 4H), 7.31 (dd, *J* = 8.0, 1.8 Hz, 4H), 7.29 – 7.07 (m, 4H), 2.34 (s, 4H), 1.62 (s, 12H), 1.25 (s, 36H).

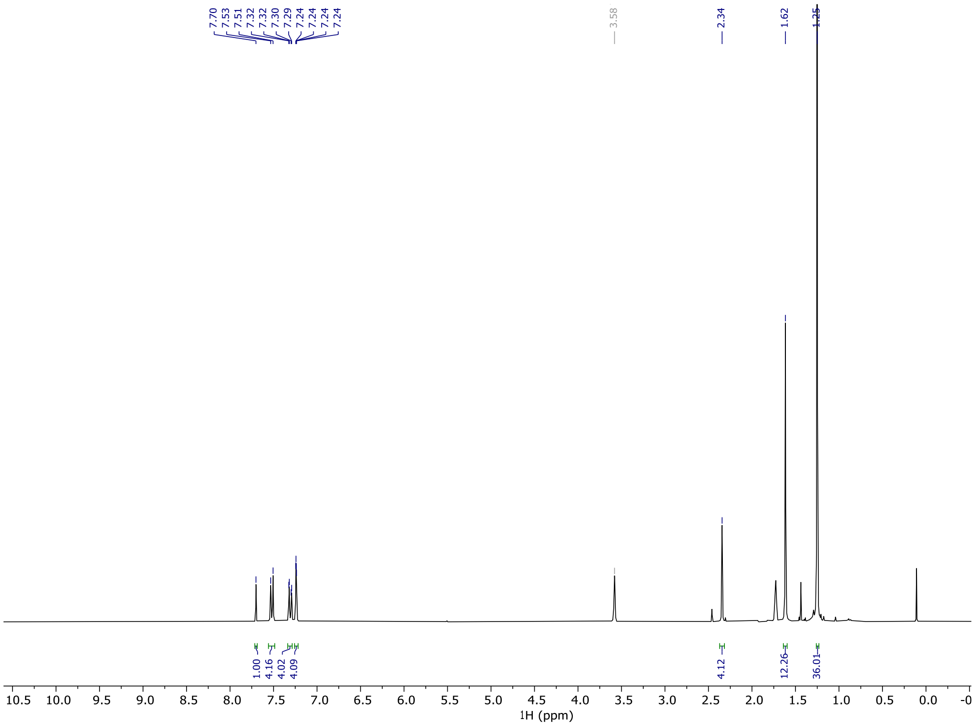

Supplementary Figure S1. ^{1}H NMR spectrum of **S2** (THF-d_8, 300 MHz, 298 K).

Synthesis of **1**: In an argon-filled glovebox, a dried-oven Schlenk was charged with **S2** (1.3 g, 1.17 mmol) and dissolved with 140 mL of anhydrous and degassed toluene. Then cobaltocene (446 mg, 2.36 mmol, 2.0 equiv.) was added portion wise under stirring. The reaction mixture was allowed to react for 18 h at room temperature, during which the solution turned pale yellow and cobaltocenium bromide precipitated. The insoluble salt was filtered off under argon, and the solvent was removed until ca. 10 mL of toluene were left. The mixture was treated with n-pentane (2×20 mL) under argon, filtered and the solid was dried under high vacuum overnight, yielding tBu-MsFluind-Bi(I) (**1**) as a bright yellow powder (780 mg, 70%).

^{1}H NMR (300 MHz, THF) δ 8.06 (d, *J* = 7.9 Hz, 4H), 7.51 (dd, *J* = 7.9, 1.8 Hz, 4H), 6.74 (d, *J* = 1.5 Hz, 4H), 1.70 (s, 4H), 1.67 (s, 36H), 1.05 (s, 12H), -1.06 (s, 1H).

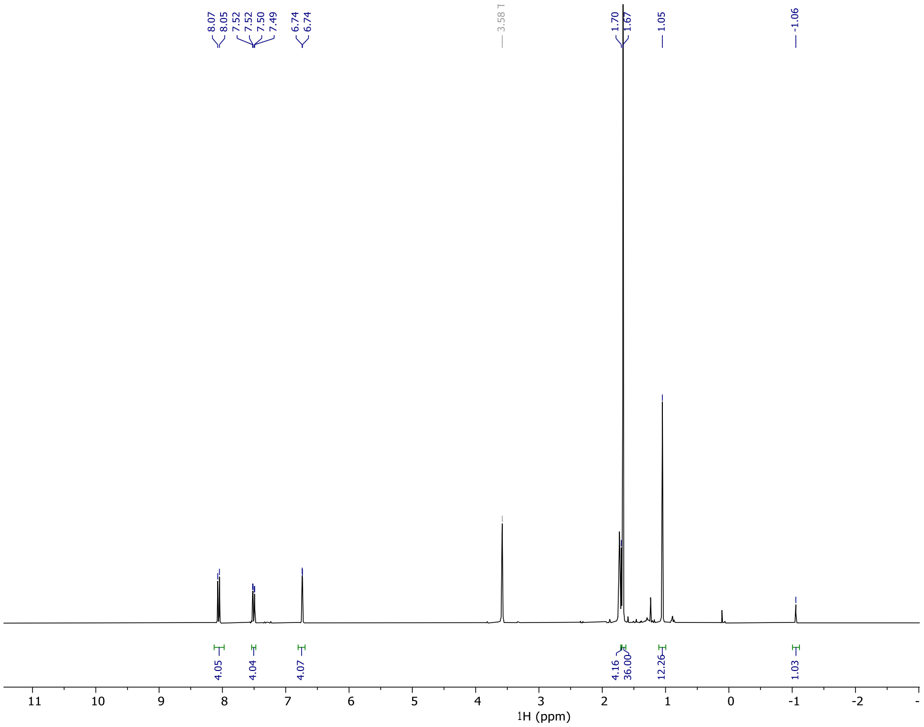

Supplementary Figure S2. ^{1}H NMR spectrum of **1** (THF-d_8, 300 MHz, 298 K).

2.2. Synthesis and characterization of **BCF • H_2O**

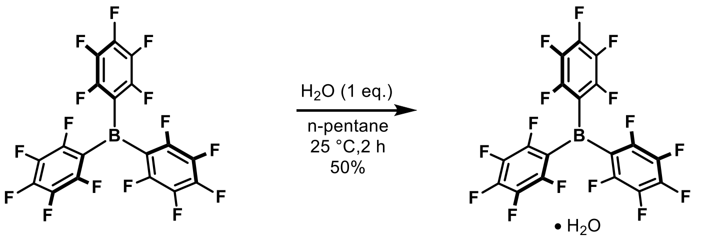

The compound was synthesized starting from $(C_6F_5)_3B$ (purchased from TCI).[2] A 2.0 µL portion of H_2O (0.11 mmol, 1 equiv.) was added into a solution of $(C_6F_5)_3B$ (56 mg, 0.11 mmol, 1 equiv.) in n-pentane (12 mL). The mixture was stirred for 2 h and then filtered. The precipitate was washed two times with pentane (5 mL) and dried under vacuum over 30 min, giving BCF • H_2O as a white solid (32 mg, 55% yield). ^{1}H NMR (300 MHz, toluene-d_8, 298 K): δ 4.63 (br). ^{19}F NMR (282 MHz,

toluene-d_8, 298 K): δ -134.96 (dd, *J* = 23.8, 8.6 Hz), -153.91 (d, *J* = 20.1 Hz), -162.37 (ddd, *J* = 24.2, 20.3, 8.7 Hz). FT-IR data: *v*OH = 3668, 3598.

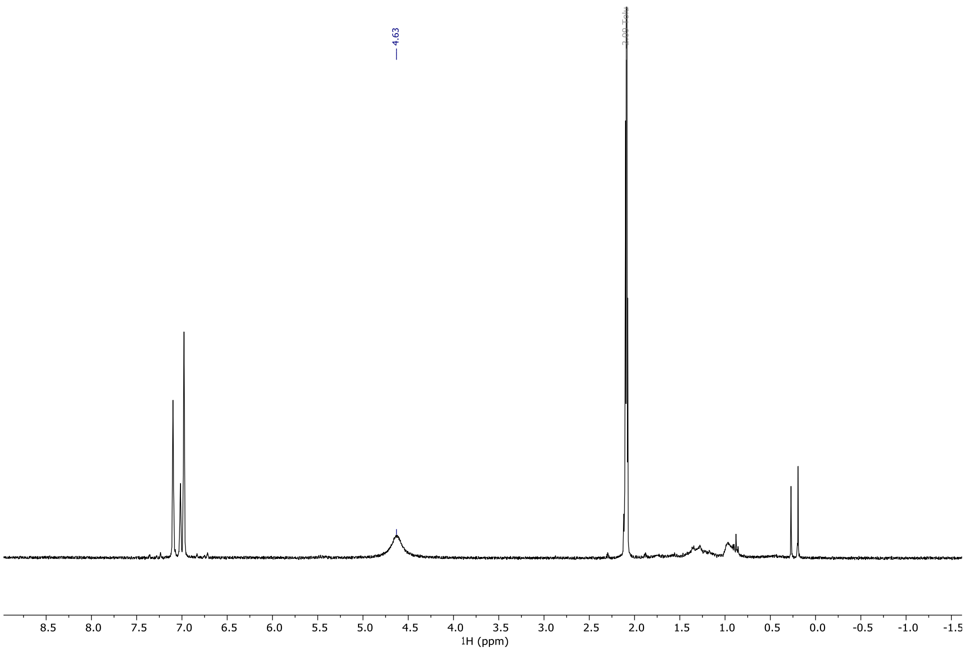

Supplementary Figure S3. ^{1}H NMR spectrum of **1** (toluene-d_8, 300 MHz, 298 K).

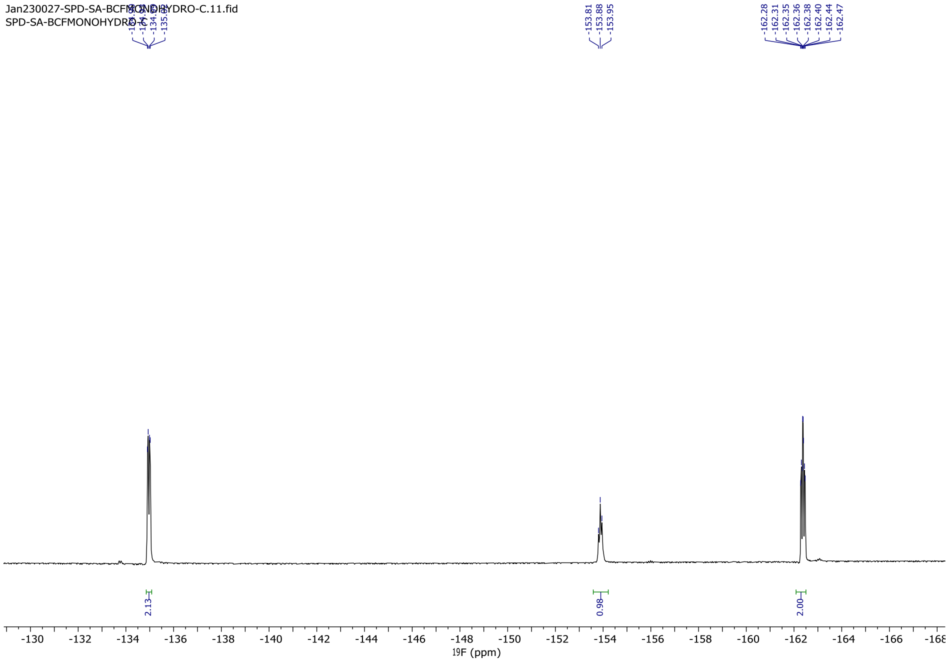

Supplementary Figure S4. ^{19}F NMR spectrum of **1** (toluene-d_8, 282 MHz, 298 K).

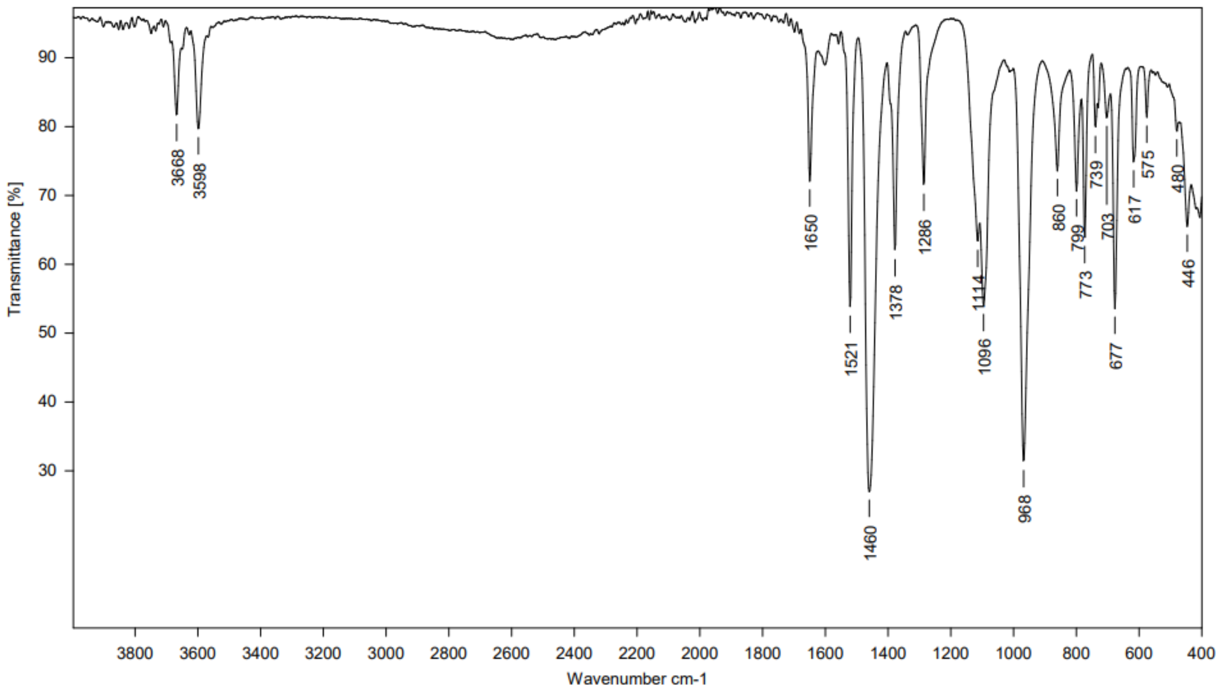

Supplementary Figure S5. FT-IR spectrum of **BCF • H$_2$O**.

3. Synthesis, Characterization and reactivity of 2

3.1. Synthesis and characterization of **2**

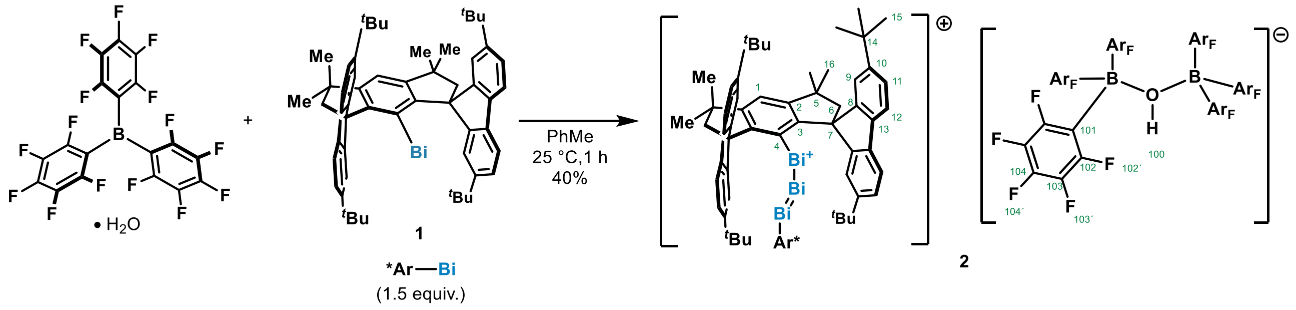

Procedure: In an argon-filled glove box, **1** (80 mg, 0.085 mmol, 1.5 equiv.) and mono-hydrated tris(pentafluorophenyl)borane (BCF) (30 mg, 0.056, 1.0 equiv.) were weighed in an oven-dried 12 mL vial, followed by the addition of 4.5 mL of anhydrous toluene. Upon stirring at 25 °C, a dark brown precipitate is formed. The mixture is stirred for 1 h and diluted with 2.0 mL of anhydrous pentane. The heterogeneous mixture is transferred to a glass-fritted filter (porosity IV) and the solid material is washed several times with anhydrous pentane (total amount 15 mL). The dark brown solid is dried under high vacuum to obtain **2** (70 mg, 79%).

^{1}H NMR (600 MHz, DCM-d_2, 298 K): δ 7.35 (d, J = 1.8 Hz, 8H, H-9), 7.21(d, J = 7.9 Hz, 8H, H-12), 7.12 (dd, J = 7.9, 1.8 Hz, 8H, H-11), 6.82 (s, 2H, H-1), 6.63 (bs, 1H, H-100), 2.11 (s, 8H, H-6), 1.50 (s, 24H, H-16), 1.23 (s, 72H, H-15).

^{13}C NMR (151 MHz, DCM-d_2, 298 K): δ 157.6 (C-8), 156.6 (C-3), 156.4 (C-2), 152.9 (C-10), 148.0 (dm, J = 240.3 Hz, C-102), 146.6 (C-4), 140.0 (C-13), 139.8 (dm, J = 248.2 Hz, C-104), 137.0 (dm, J = 247.7 Hz, C-103), 126.2 (C-11), 125.0 (C-9), 123.1 (C-1), 122.2 (C-12), 66.6 (C-7), 58.4 (C-6), 44.1 (C-5), 35.1 (C-14), 32.2 (C-15), 32.1 (C-16). *Signal of C-101 (119.5 ppm) was only visible in a ^{13}C{^{19}F} NMR spectrum.

^{19}F NMR (564 MHz, DCM-d_2, 298 K): δ –133.51 (bs), –159.74 (t, J = 19.9 Hz), –165.79 (t, J = 20.2 Hz).

M.p.: the compound melts at 295 °C in a flame-sealed argon-filled capillary.

HRMS (ESI pos): calc'd for $C_{112}H_{130}Bi_3^+$ $[M]^+$ 2101.95790; found 2101.9602.

Stability: air sensitive.

XRD: single crystals of **2** suitable for X-ray diffraction analysis were obtained by layering a filtered toluene-d_8 solution of Ar^{F}B•(H_2O) on a filtered solution of **1** in toluene-d_8. The mixture was then shaken for ca. 2 min. and left resting at 30 °C until crystals appeared at the bottom of the vial (ca. 2 d).

Anal. calc'd for **2**• 2 toluene-d_8 ($C_{162}H_{131}D_{16}B_2Bi_3F_{30}O$): C, 58.18; H, 4.91;D, -;* B, 0.65; Bi, 18.75; F, 17.04; O, 0.48;* found C, 58.30; H, 4.09; B, 0.63; Bi, 18.58; F, 16.87. * Deuterium and Oxygen could not be measured.

Nuclear magnetic resonance spectroscopy (NMR) spectra

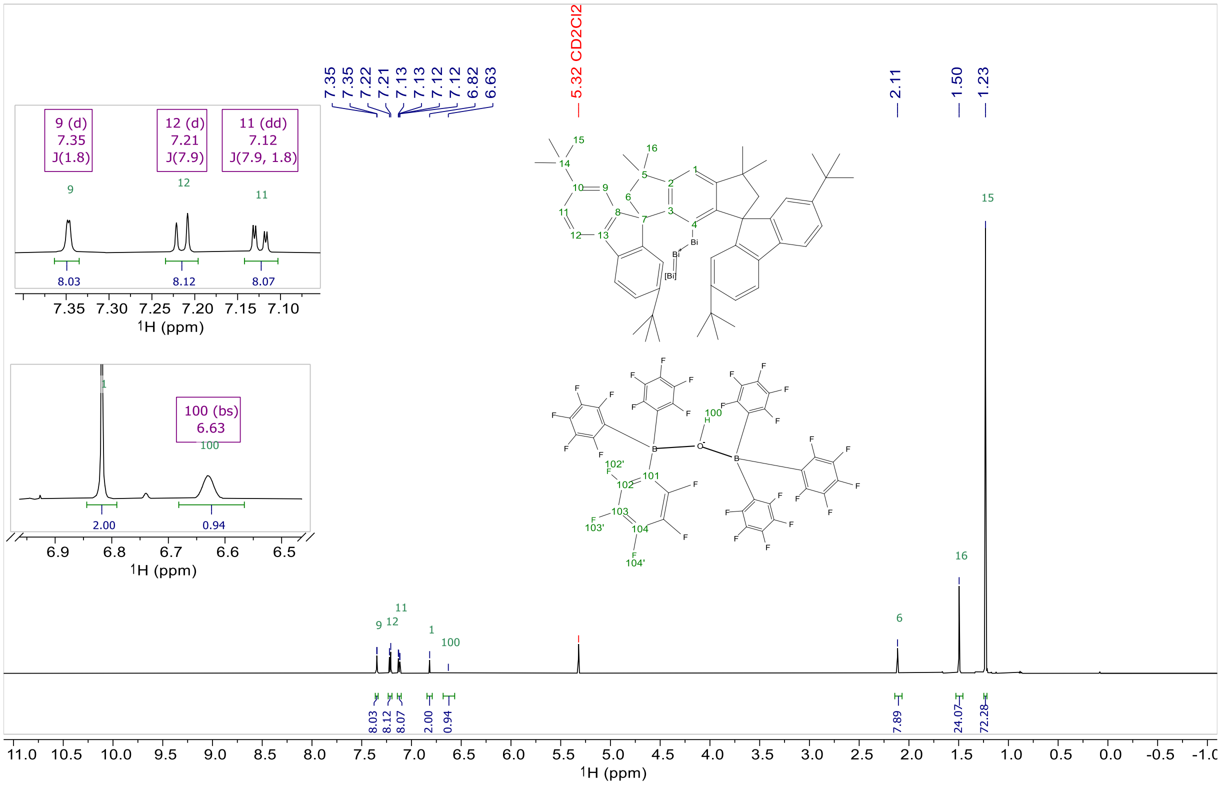

Supplementary Figure S6. ^{1}H NMR spectrum of **2** (DCM-d_2, 600 MHz, 298 K).

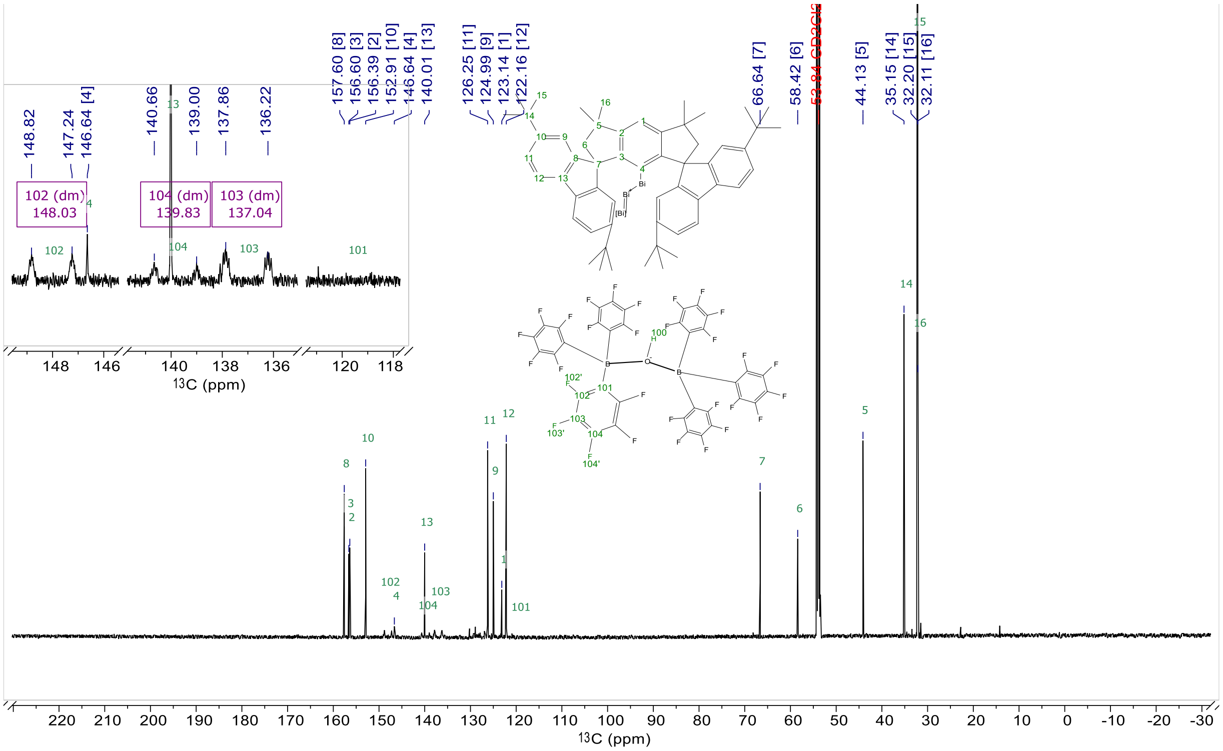

Supplementary Figure S7. ^{13}C NMR spectrum of **2** (DCM-d_2, 151 MHz, 298 K).

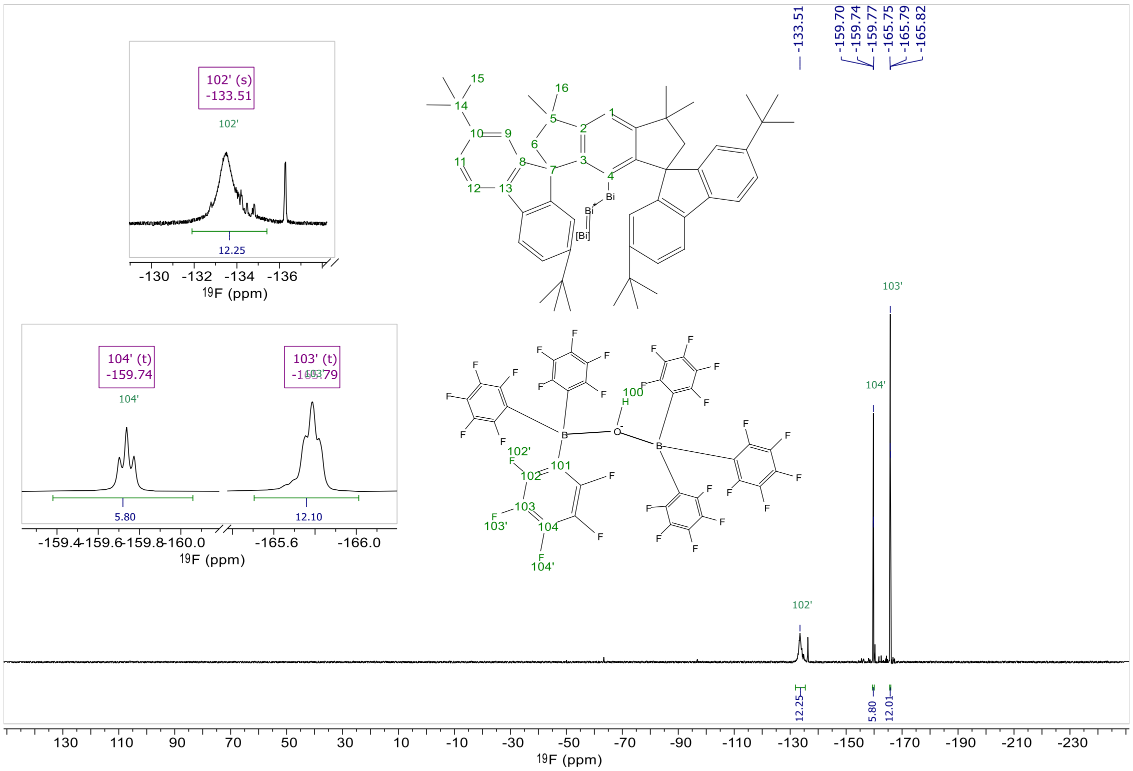

Supplementary Figure S8. ^{19}F NMR spectrum of **2** (DCM-d_2, 564 MHz, 298 K).

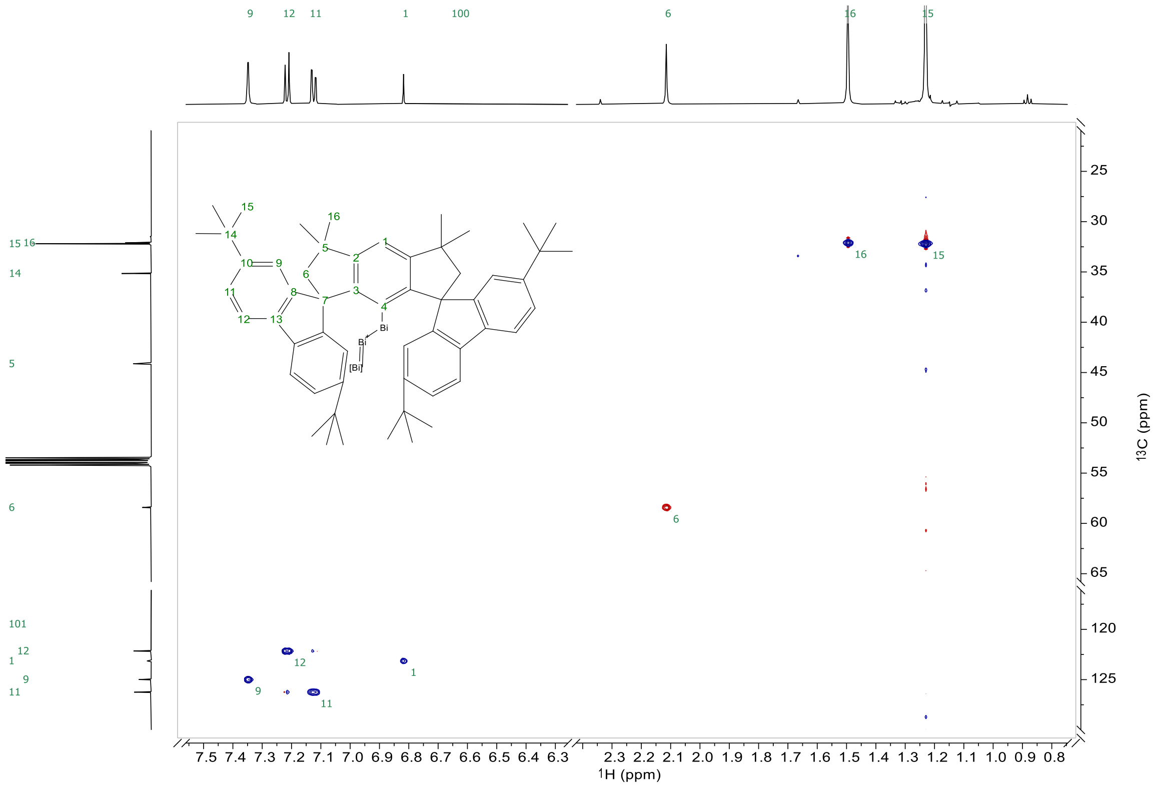

Supplementary Figure S9. ^{1}H-^{13}C edited HSQC spectrum of **2** (DCM-d_2, 600 MHz, 298 K).

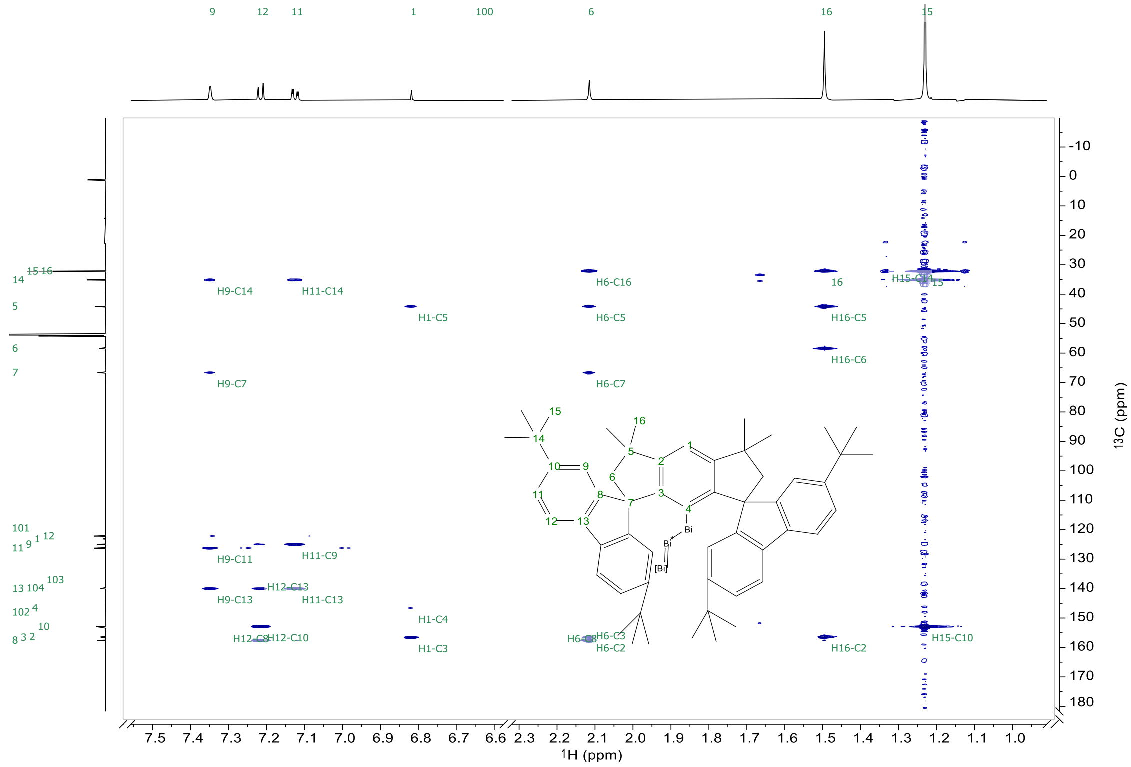

Supplementary Figure S10. ^{1}H-^{13}C HMBC spectrum of **2** (DCM-d_2, 600 MHz, 298 K).

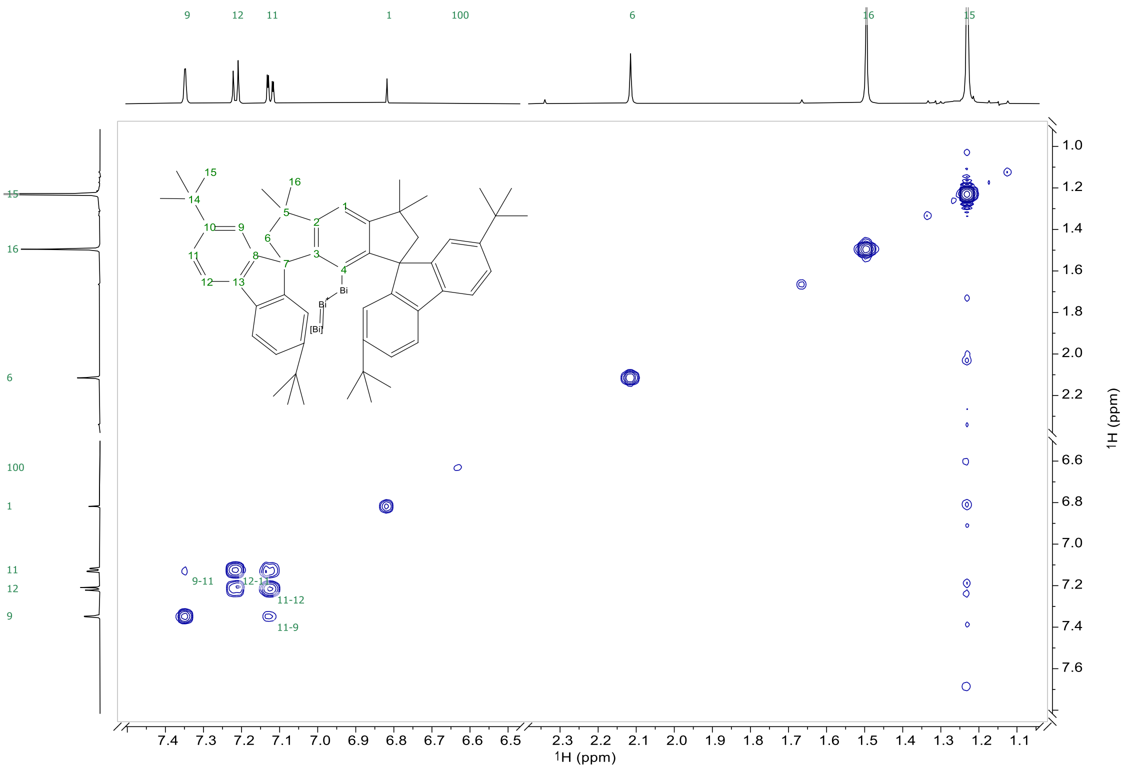

Supplementary Figure S11. ^{1}H-^{1}H COSY spectrum of **2** (DCM-d_2, 600 MHz, 298 K).

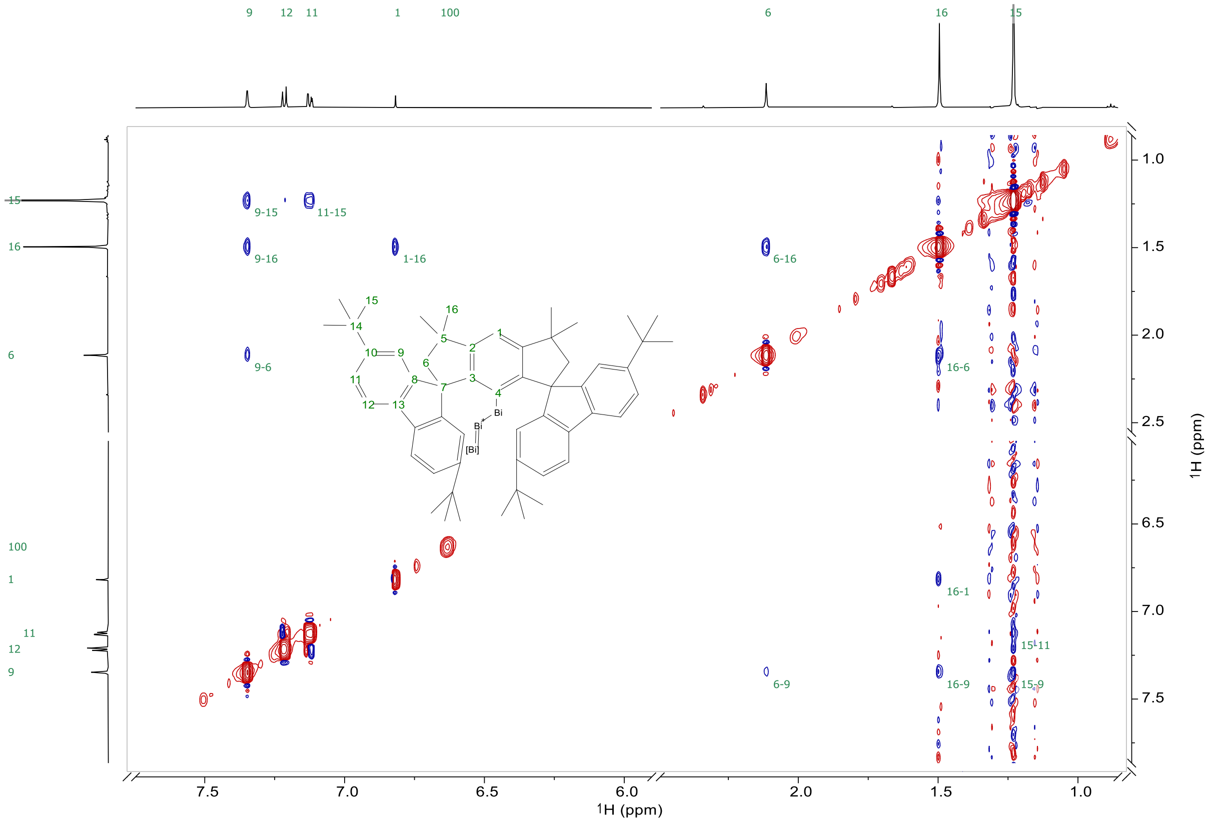

Supplementary Figure S12. ^{1}H-^{1}H EASY-ROESY spectrum of **2** (DCM-d_2, 600 MHz, 298 K).

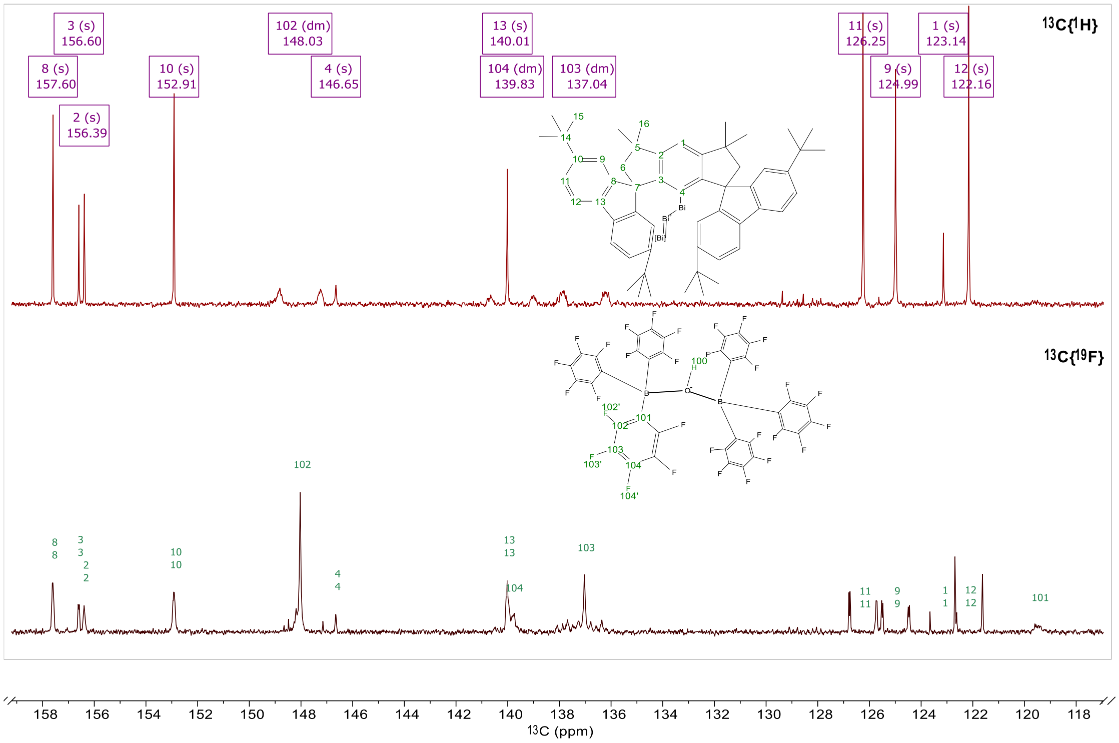

Supplementary Figure S13. Stacked spectra: $^{13}C\{^1H\}$ & $^{13}C\{^{19}F\}$ of **2** (DCM-d_2, 298 K).

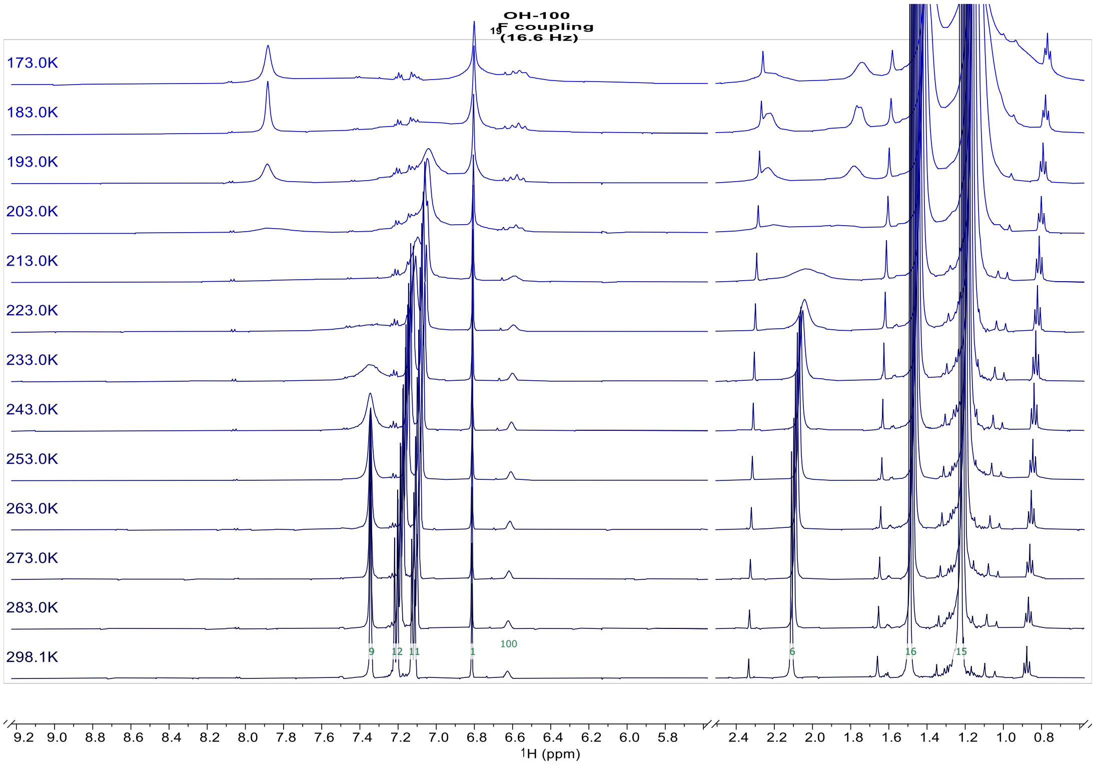

Supplementary Figure S14. Stacked Variable Temperature (VT) ^{1}H NMR spectra of **2** (DCM-d_2, 500 MHz).

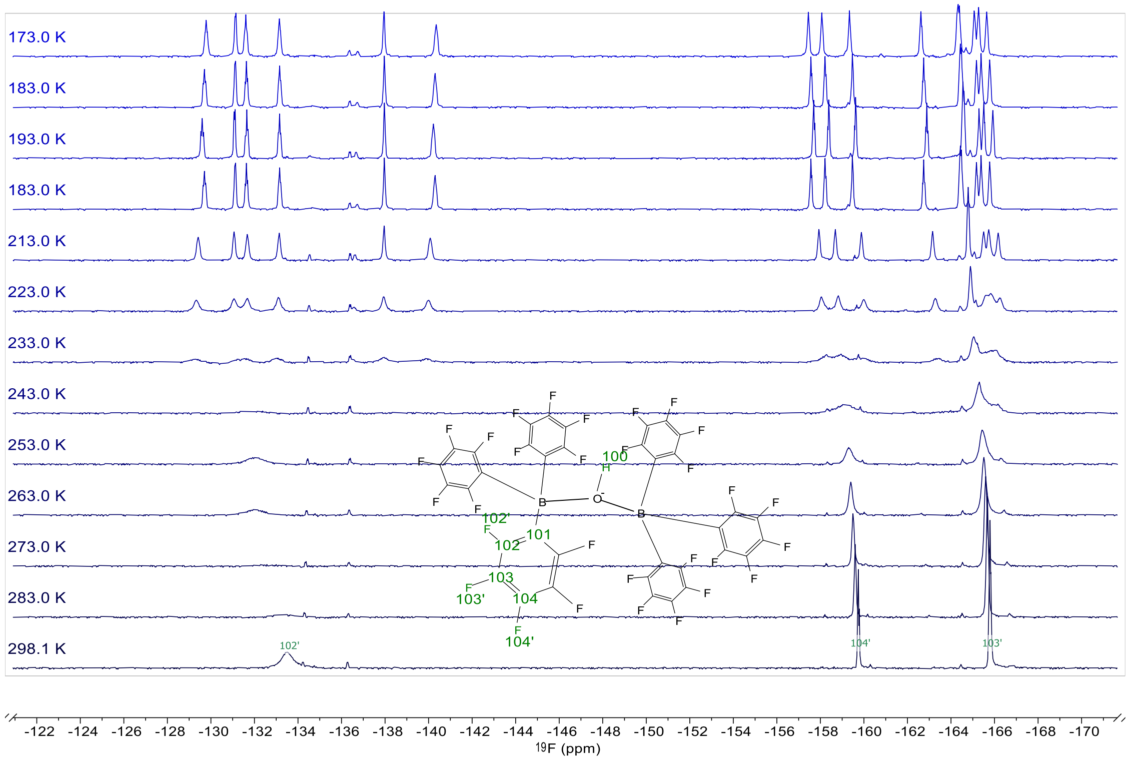

Supplementary Figure S15. Stacked VT ^{19}F NMR spectra of **2** (DCM-d_2, 470.3 MHz).

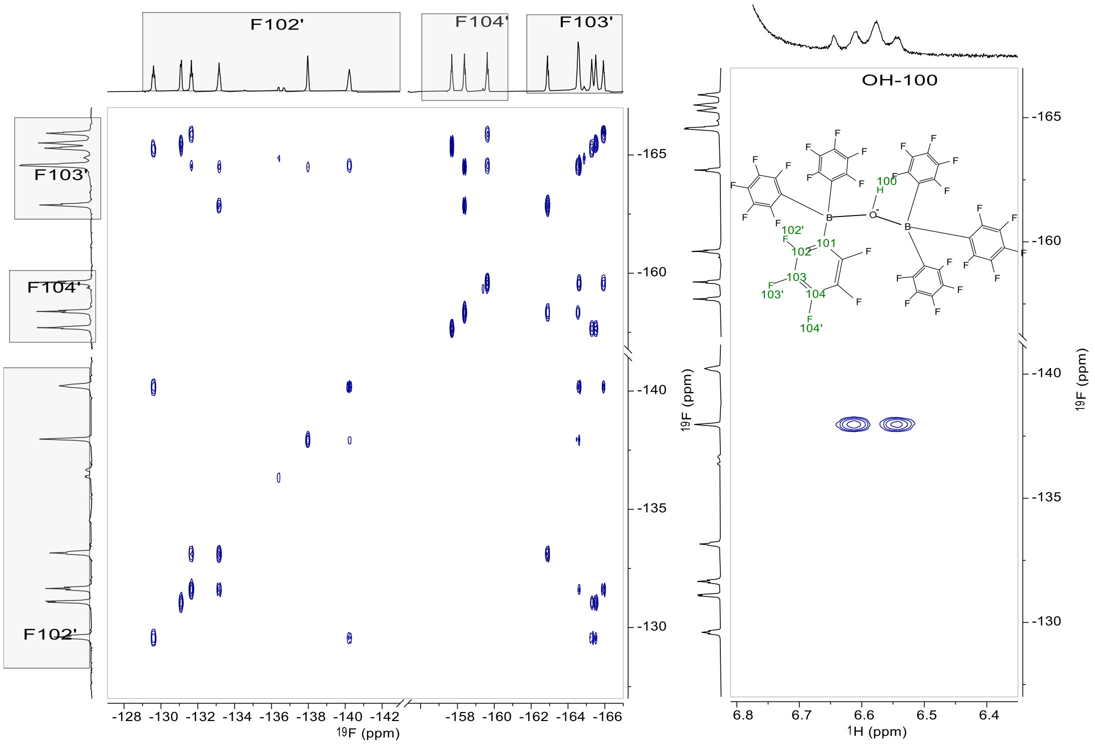

Supplementary Figure S16. ^{19}F-^{19}F COSY of **2** (DCM-d_2, 470.3 MHz, 193 K).

Elemental analysis for **2•** 2 toluene-d_8*:*

MIKROLAB
Mikroanalytisches Laboratorium Kolbe

Dr. Davide Spinnato

Max-Planck-Institut für Kohlenforschung
Dr. Josep Cornellà
Nachhaltige Katalyse für die Organische Synthese

Anschrift : Osterfelder Str. 3
D-46047 Oberhausen
Telefon : +49 - (0)208 - 32502
Telefax : +49 - (0)208 - 382314
Email : info@mikro-lab.de
Webseite : www.mikro-lab.de

Datum : 13.06.2024

Probenbezeichnung	% C	% H	% Bi	% F	% B	% Dry					Argon	V2O5
SPD - SA - 700 - 00	58,30	4,09	18,58	16,87	0,63	0,25					x	x

*) Sauerstoff aufgrund chemischer Störungen nicht bestimmbar
*) Deuterium nicht bestimmbar

Mit freundlichen Grüssen
Patrick Springer

UV-Vis-NIR

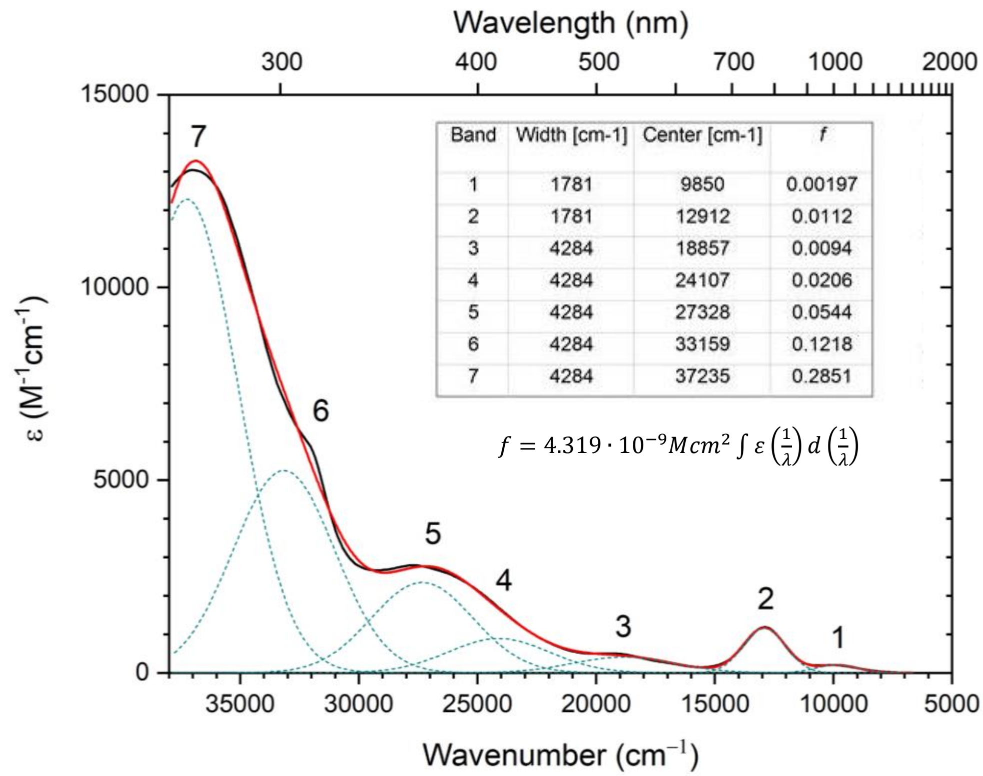

Band	Width [cm-1]	Center [cm-1]	*f*
1	1781	9850	0.00197
2	1781	12912	0.0112
3	4284	18857	0.0094
4	4284	24107	0.0206
5	4284	27328	0.0544
6	4284	33159	0.1218
7	4284	37235	0.2851

Supplementary Figure S17. UV-Vis-NIR (0.80 mM of **2** in DCM). Experimental data (black trace); overall fit (red trace); 7 individual Gaussian bands fit to the data (dashed light blue traces) and their mathematical parametrization.

IR:

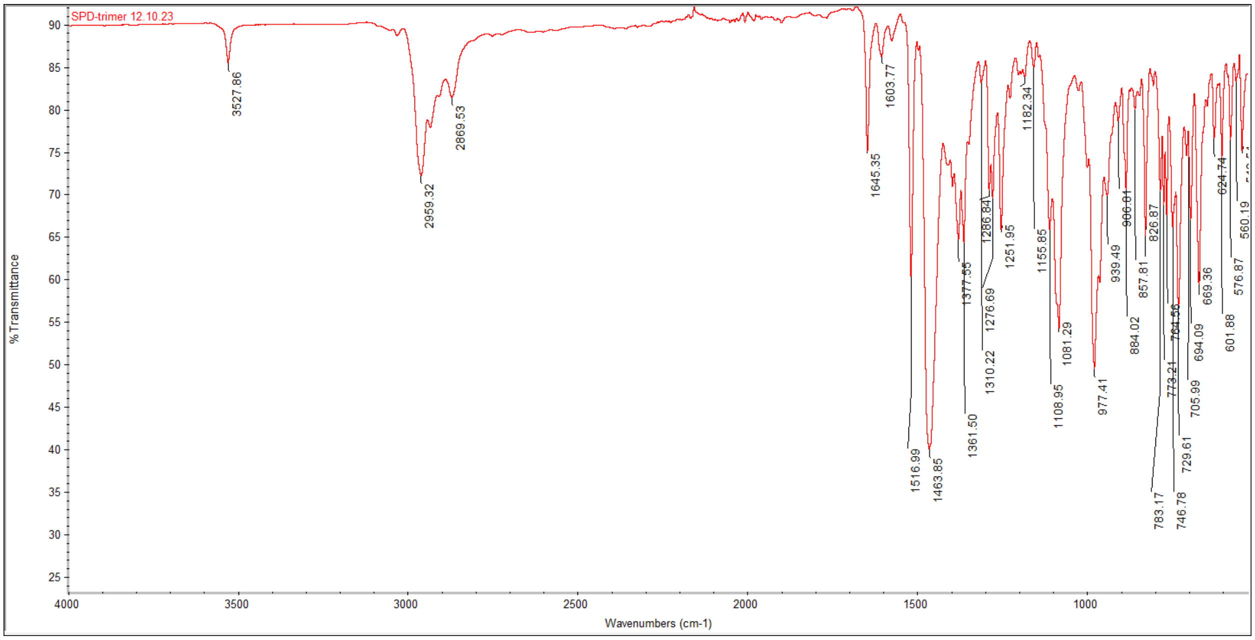

Supplementary Figure S18. FT-IR spectrum of **2**.

Cyclic voltammetry studies

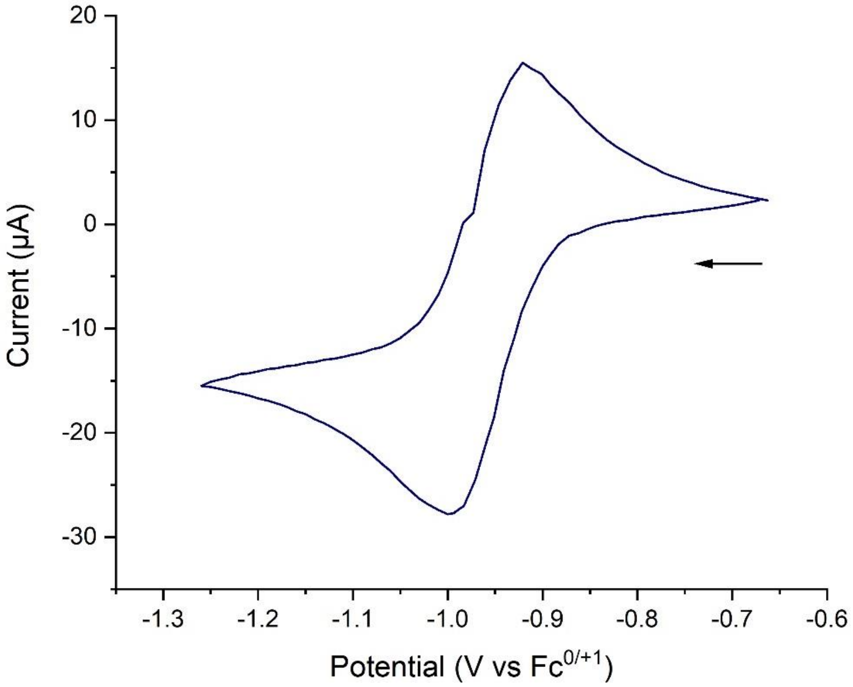

Supplementary Figure S19. Cyclic voltammogram of **2** (cathodic scan) in dichloromethane using 0.10 M [nBu$_4$N][BArF] as supporting electrolyte at ambient temperature; scan rate: 100 mV/s, referenced to Fc$^{0/+}$.

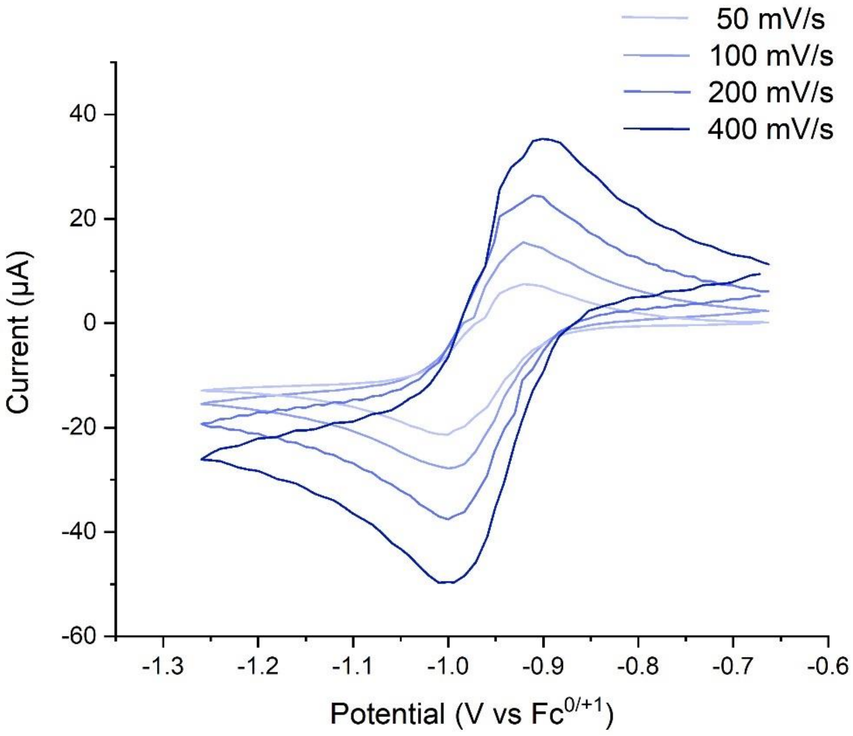

Supplementary Figure S20. Cyclic voltammogram of **2** (cathodic scan) in dichloromethane using 0.10 M [nBu$_4$N][BArF] as supporting electrolyte at ambient temperature; variable scan rate: from 50mV/s to 400 mV/s, referenced to Fc$^{0/+}$.

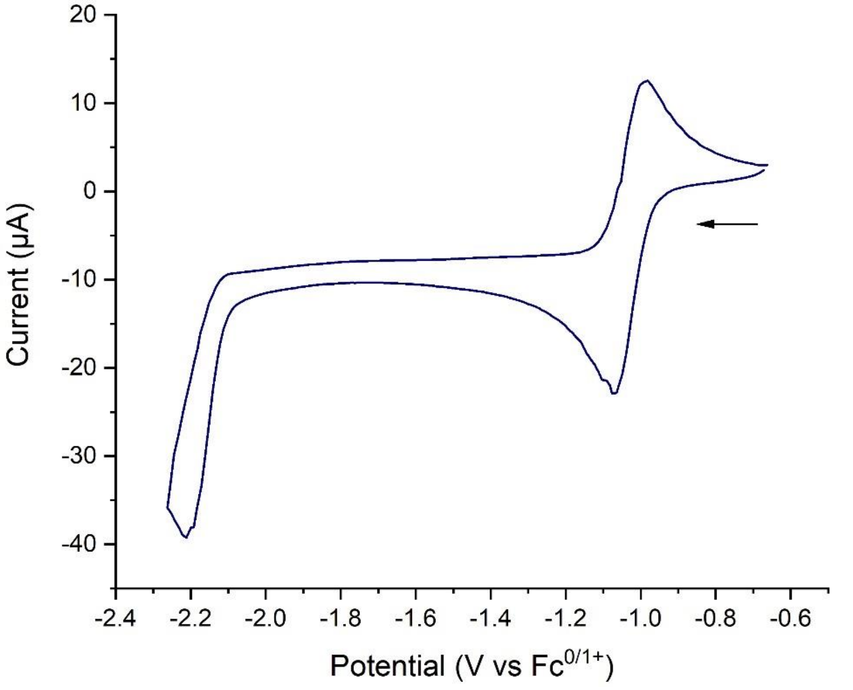

Supplementary Figure S21. Extended cyclic voltammogram of **2** (cathodic scan) in dichloromethane using 0.10 M [nBu$_4$N][BArF] as supporting electrolyte at ambient temperature; scan rate: 100 mV/s, referenced to Fc$^{0/+}$.

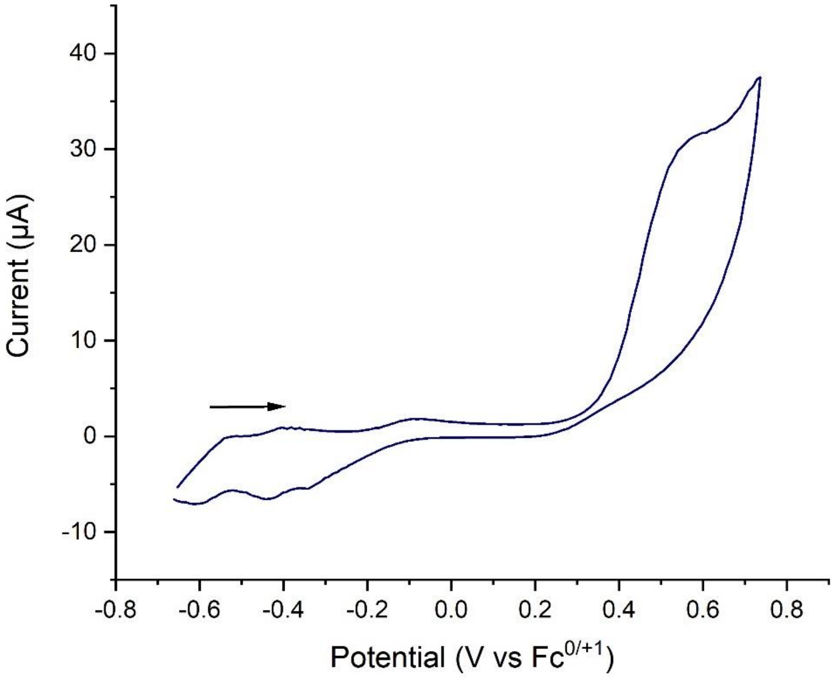

Supplementary Figure S22. Cyclic voltammogram of **2** (anodic scan) in dichloromethane using 0.10 M [nBu$_4$N][BArF] as supporting electrolyte at ambient temperature; scan rate: 100 mV/s, referenced to Fc$^{0/+}$.

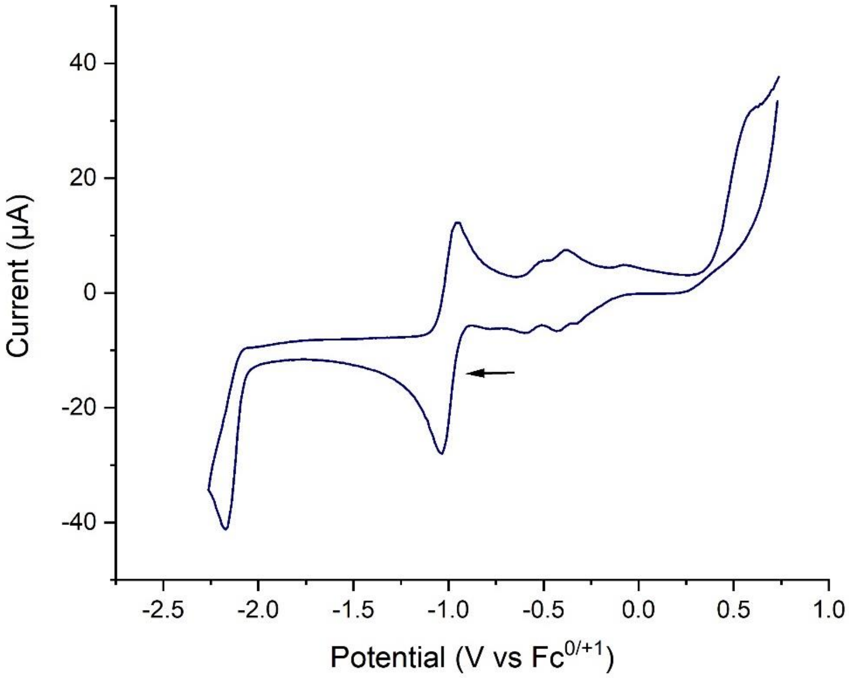

Supplementary Figure S23. Full cyclic voltammogram of **2** in dichloromethane using 0.10 M [nBu$_4$N][BArF] as supporting electrolyte at ambient temperature; scan rate: 100 mV/s, referenced to Fc$^{0/+}$.

Complex **2** shows a perfectly reversible reduction event at –0.95V (reference to $Fc^{0/+1}$) which we assigned to the single electron reduction of the $(Bi)_3^+$ delivering the corresponding $(Bi)_3^{\bullet}$. Upon further scanning at more negative potential (–2.1V referenced to $Fc^{0/+1}$) a second, but irreversible, redox event takes place (Supplementary Figure S21). This second event promote the appearance of small, but clearly detectable, redox event at around –0.20V which cannot be detected when scanning starts at –0.60V (Supplementary Figure S22 and Supplementary Figure S23). When scanning is performed starting from –0.60V up to 1.0 V, an irreversible oxidation event occur at ca. +0.75V.

3.2. Synthesis and characterization of **2'**

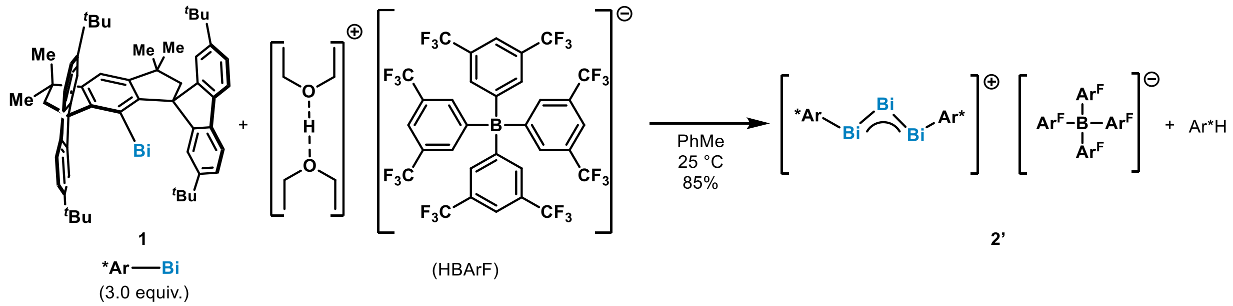

Procedure: In an argon-filled glove box, **1** (126 mg, 0.133 mmol, 3.00 equiv.) and HBArF•2 Et_2O (45.0 mg, 0.044, 1.00 equiv.) were weighed in an oven-dried in a 25.0 mL Schlenk tube, followed by the addition of 4.00 mL of toluene (anhydrous). Upon stirring at 25 °C, a dark brown precipitate is formed. The mixture is stirred for 15 min and diluted with 2.00 mL of anhydrous pentane. The heterogeneous mixture is transferred to a glass-fritted filter (porosity IV) and the solid material is washed several times with anhydrous pentane (total amount 20.0 mL). The dark brown solid is dried under high vacuum to obtain **2'** (112 mg, 0.038 mmol, 85%).

Characterization data of **2'**:

^{1}H NMR (600 MHz, DCM-*d*$_2$, 298 K): δ 7.74 – 7.70 (m, 8H, H-102), 7.56 (s, 4H, H-104), 7.35 (d, *J* = 1.8 Hz, 8H, H-9), 7.22 (dd, *J* = 7.9, 0.5 Hz, 8H, H-12), 7.12 (dd, *J* = 7.9, 1.8 Hz, 8H, H-11), 6.82 (s, 2H, H-1), 2.11 (s, 8H, H-6), 1.49 (s, 24H, H-6), 1.23 (s, 72H, H-72).

^{13}C NMR (151 MHz, DCM-*d*$_2$, 298 K): δ δ 162.2 (q, *J* = 49.8 Hz, C-101), 157.6 (C-8), 156.6 (C-3), 156.39 (C-2), 152.9 (C-10), 146.6 (C-4), 140.0 (C-13), 135.4 – 134.8 (m, C-102), 129.3 (qq, *J* = 31.5, 3.0 Hz, C-103), 126.2 (C-11), 125.0 (q, *J* = 272.2 Hz, C-105), 124.9 (C-9), 123.1 (C-1), 122.2, (C-12) 118.0 – 117.8 (m, C-104), 66.6 (C-7), 58. (C-6), 44.13 (C-5), 35.2 (C-14), 32.2 (C-15), 32.1 (C-16).

^{11}B (192 MHz, DCM-*d*$_2$, 298 K) δ –6.6.

^{19}F NMR (564 MHz, DCM-*d*$_2$, 298 K): *δ* –62.9.

M.p.: the compound melts at 310°C in a flame-sealed argon-filled capillary.

HRMS (ESI pos): calc'd for $C_{112}H_{130}Bi_3^+$ $[M]^+$ 2101.95747; found 2101.95790.

Stability: air sensitive.

XRD: single crystals of **2’** suitable for X-ray diffraction analysis were obtained by slow diffusion of pentane into a chlorobenzene solution of **2’** at room temperature; crystals appeared after 24 h.

Nuclear magnetic resonance spectroscopy (NMR) spectra

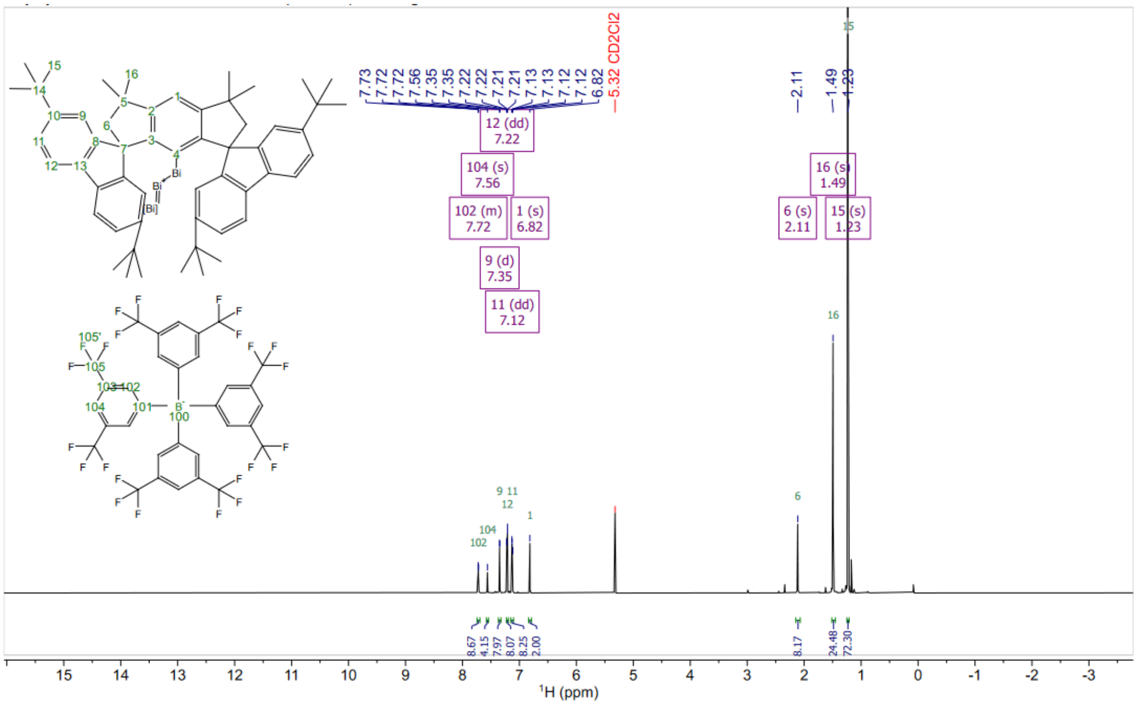

Supplementary Figure S24. ^{1}H NMR spectrum of **2’** (DCM-d_2, 600 MHz, 298 K).

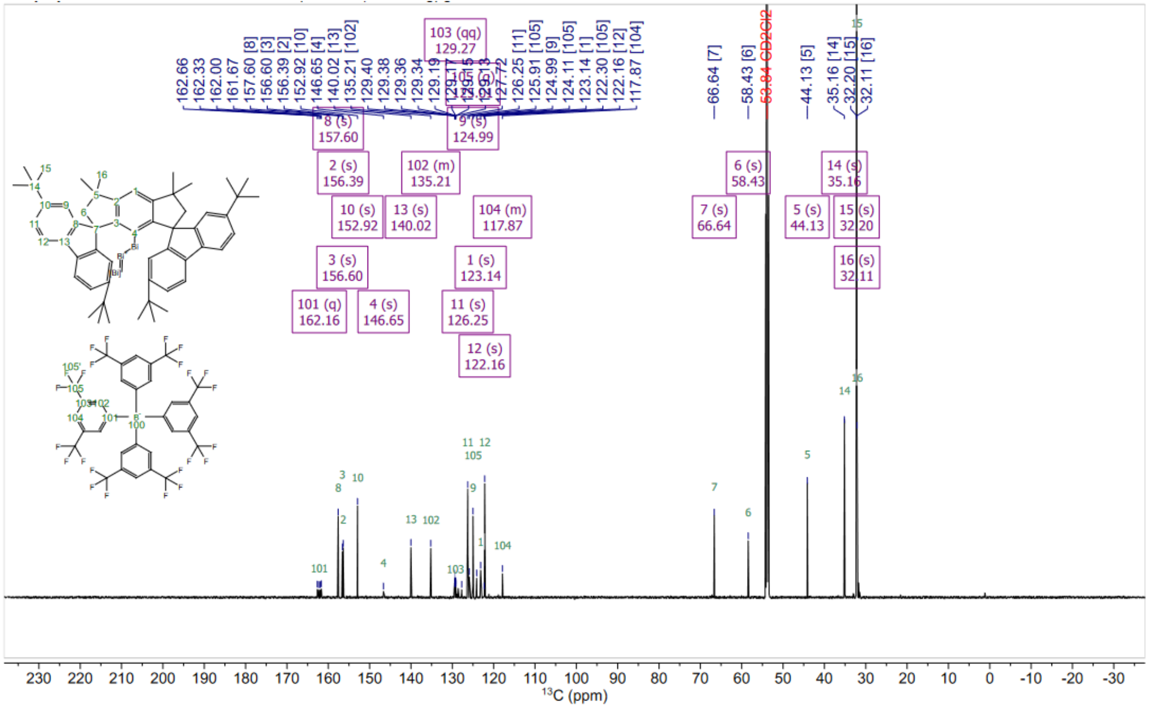

Supplementary Figure S25. ^{13}C NMR spectrum of **2’** (DCM-d_2, 151 MHz, 298 K).

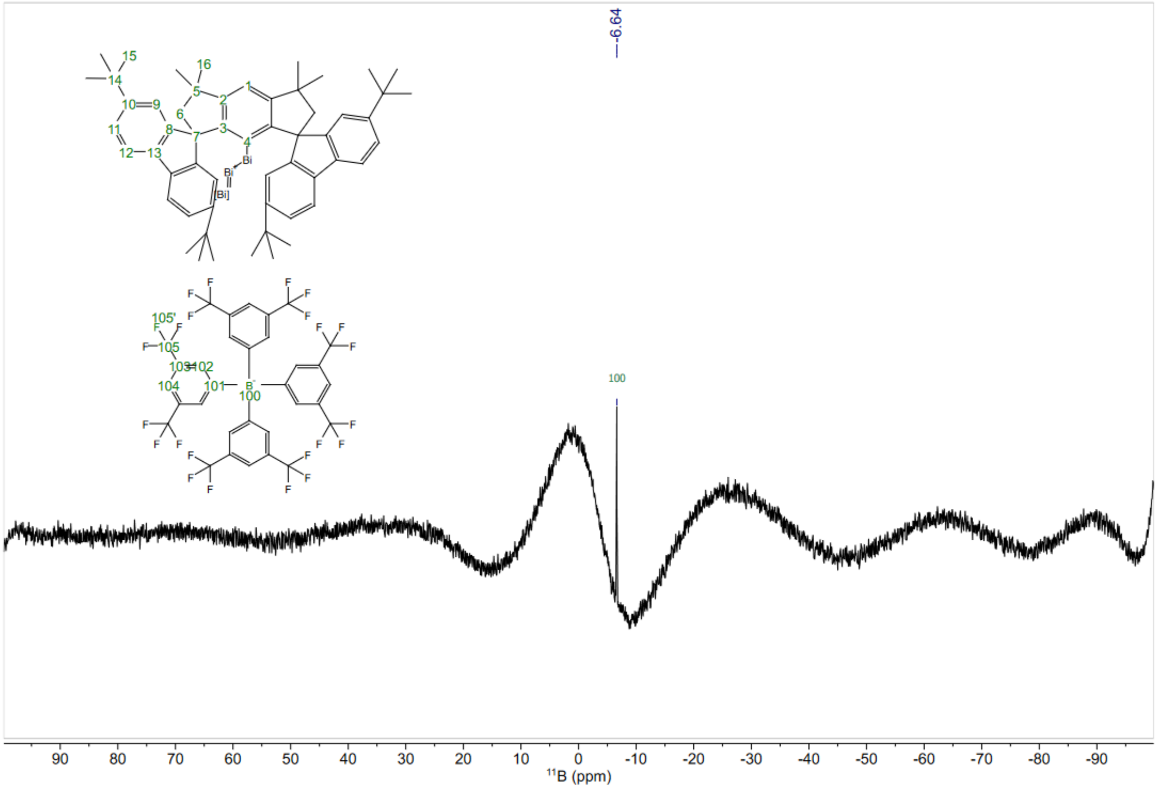

Supplementary Figure S26. ^{11}B NMR spectrum of **2’** (DCM-d_2, 192 MHz, 298 K).

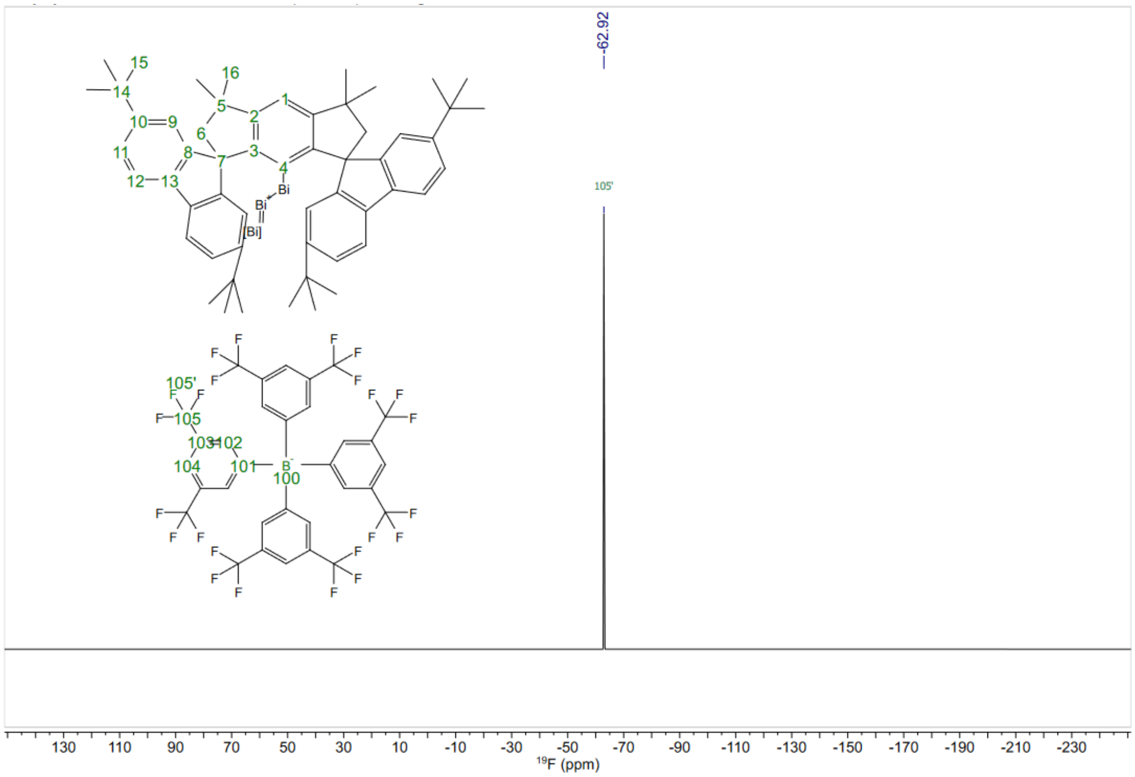

Supplementary Figure S27. ^{19}F NMR spectrum of **2’** (DCM-d_2, 564 MHz, 298 K).

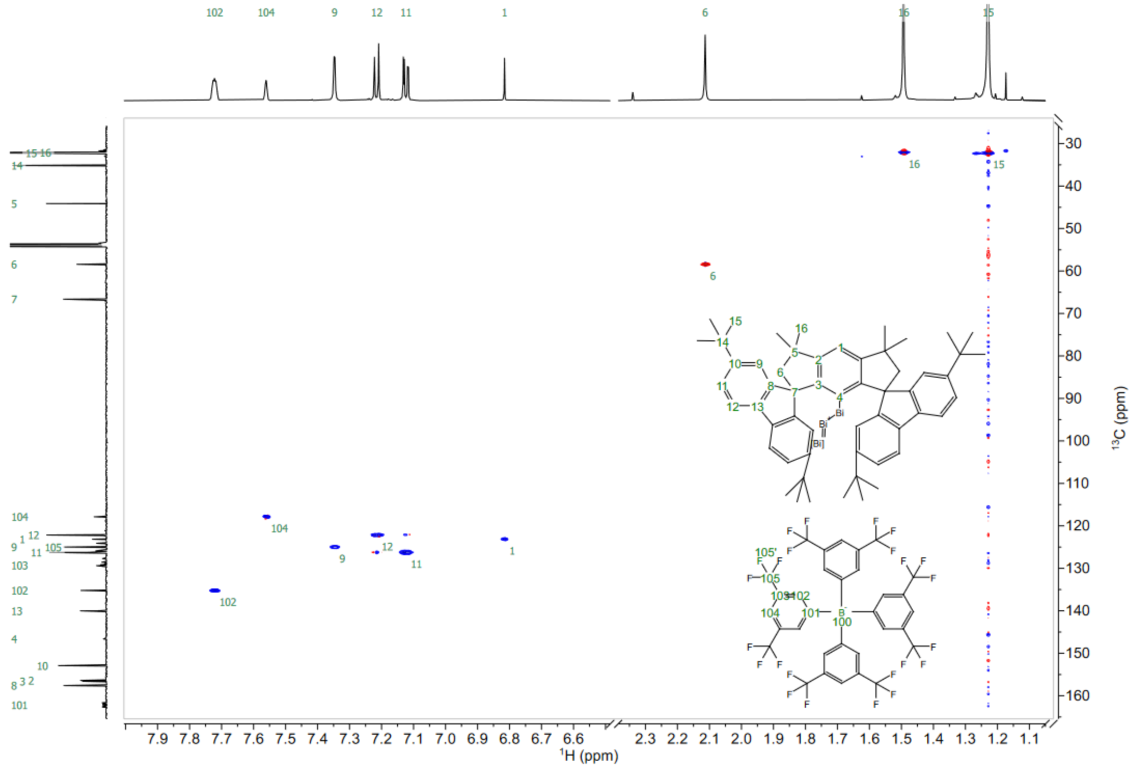

Supplementary Figure S28. ^{1}H-^{13}C edited HSQC spectrum of **2'** (DCM-d_2, 600 MHz, 298 K).

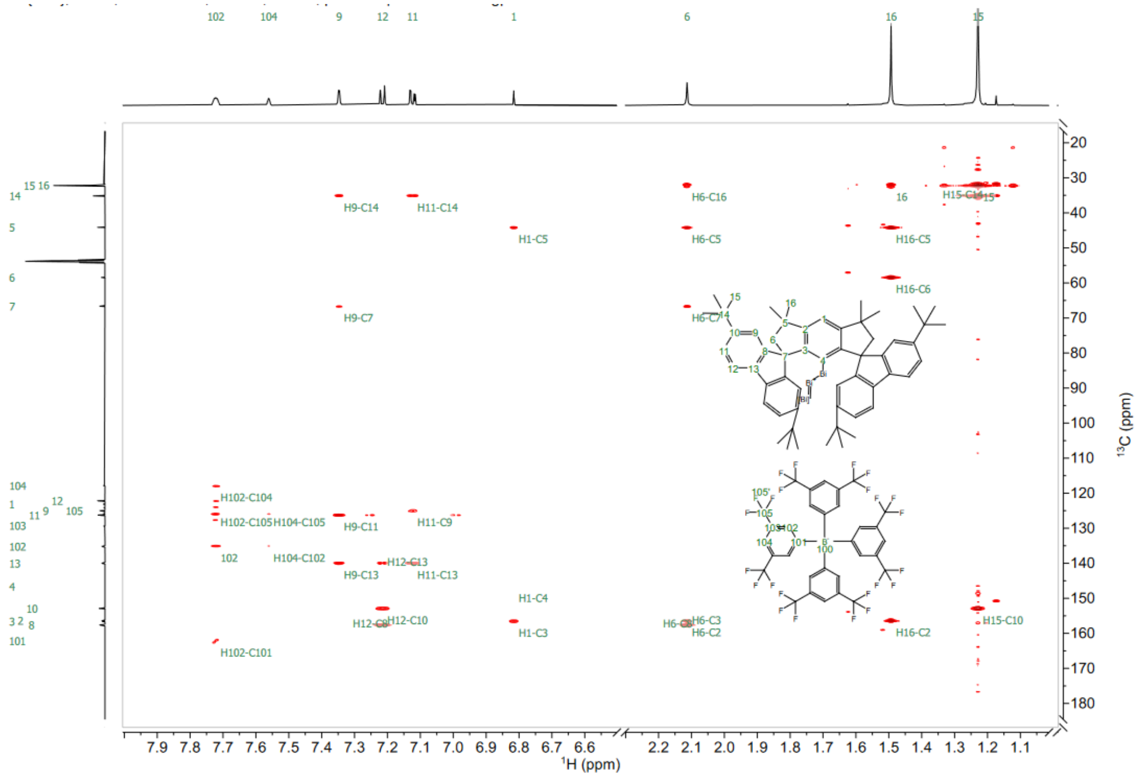

Supplementary Figure S29. ^{1}H-^{13}C HMBC spectrum of **2'** (DCM-d_2, 600 MHz, 298 K).

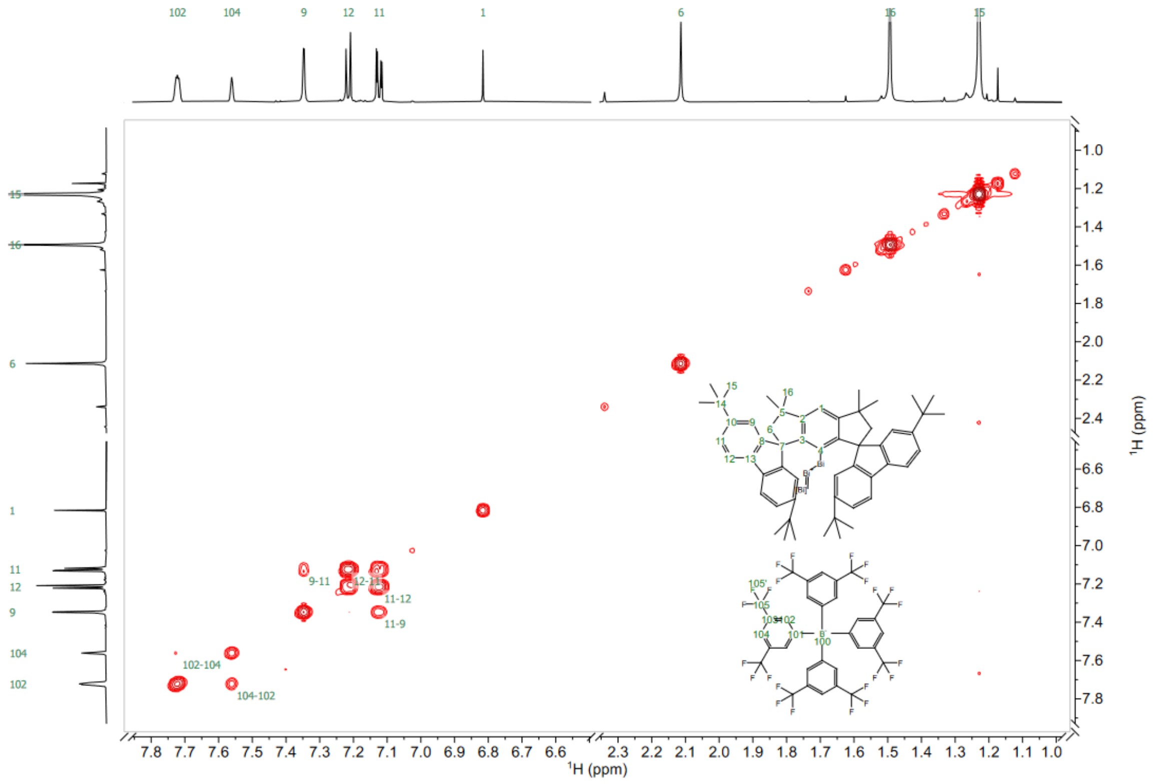

Supplementary Figure S30. ^1H-^1H COSY spectrum of **2’** (DCM-d_2, 600 MHz, 298 K).

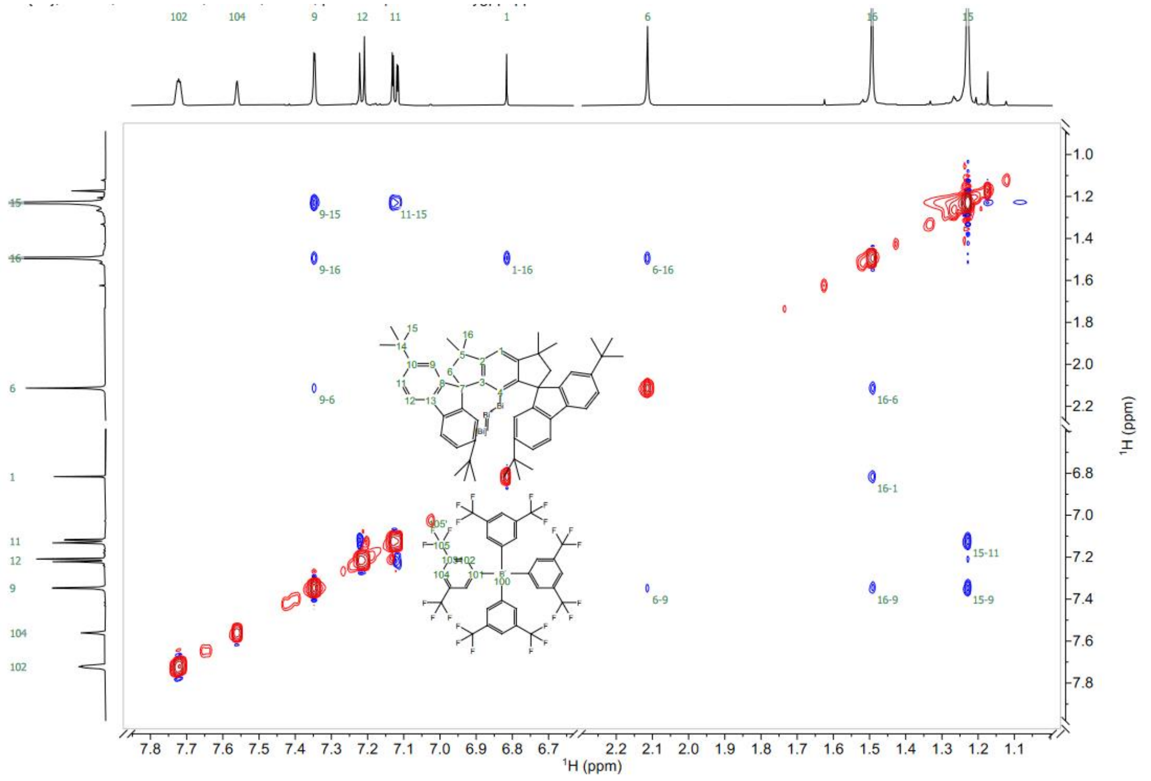

Supplementary Figure S31. ^1H-^1H NOESY spectrum of **2’** (DCM-d_2, 600 MHz, 298 K).

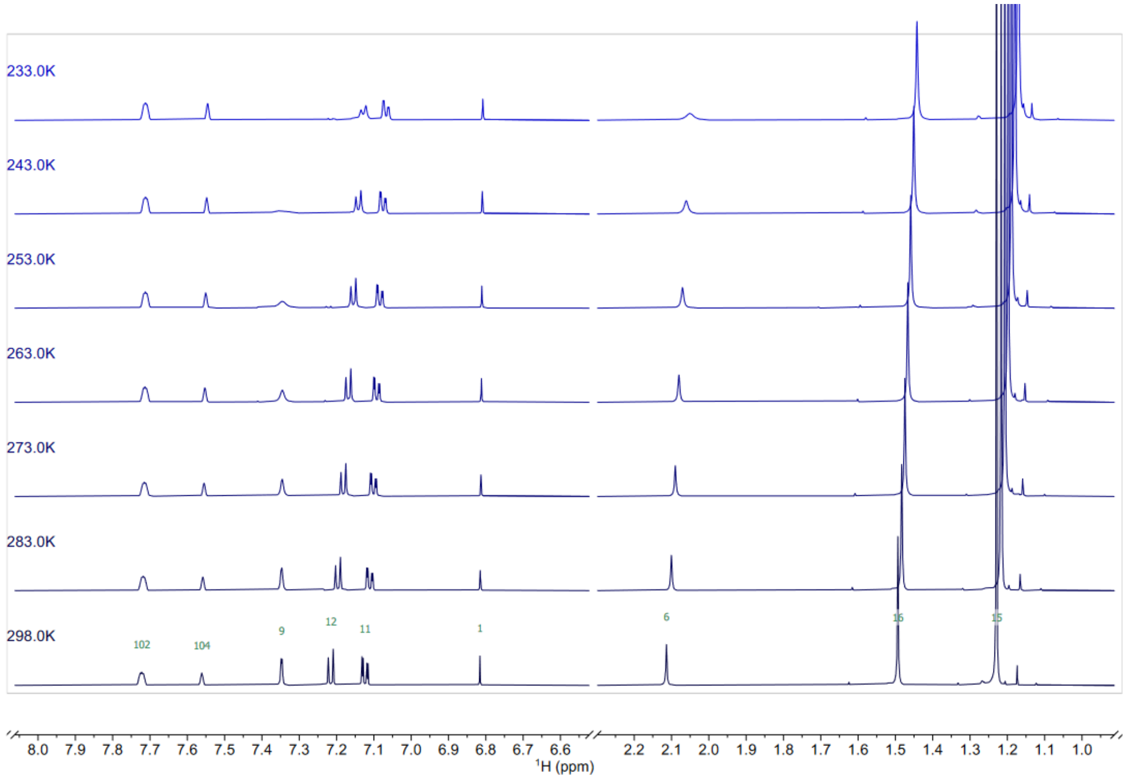

Supplementary Figure S32. VT ^{1}H NMR spectra of **2’** (DCM-d_2, 600 MHz).

UV-Vis-NIR

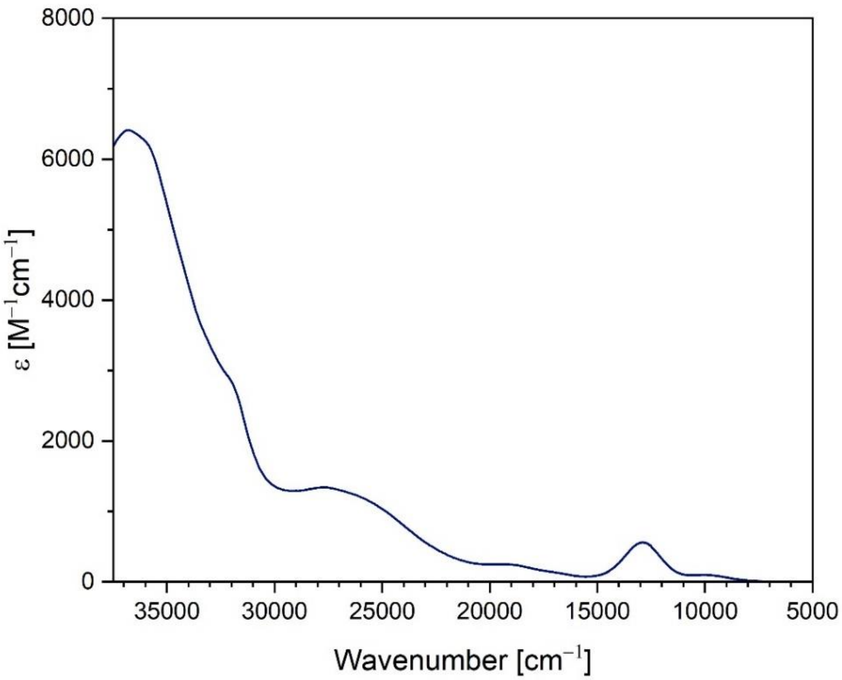

Supplementary Figure S33. UV-Vis-NIR (1.1 mM of **2’** in DCM).

Comment: The UV-Vis absorption spectra is comparable with the measurement of **2**, thus supporting the existence of the same Bi_3^+ core chromophore (*cfr*. Supplementary Figure S17).

Cyclic voltammetry studies

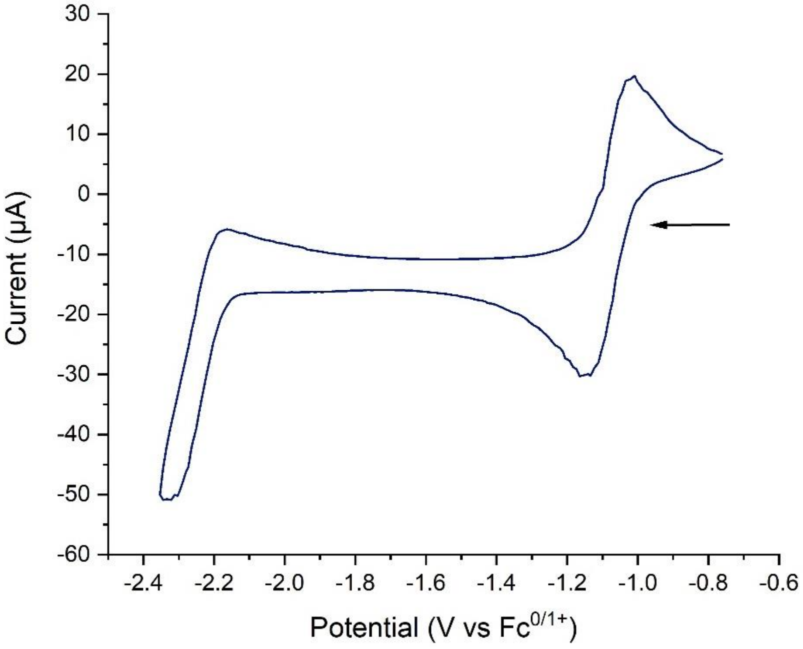

Supplementary Figure S34. Extended cyclic voltammogram of **2’** (cathodic scan) in dichloromethane using 0.10 M [nBu$_4$N][BArF] as supporting electrolyte at ambient temperature; scan rate: 100 mV/s, referenced to $Fc^{0/+}$(*cfr*. Supplementary Figure S21).

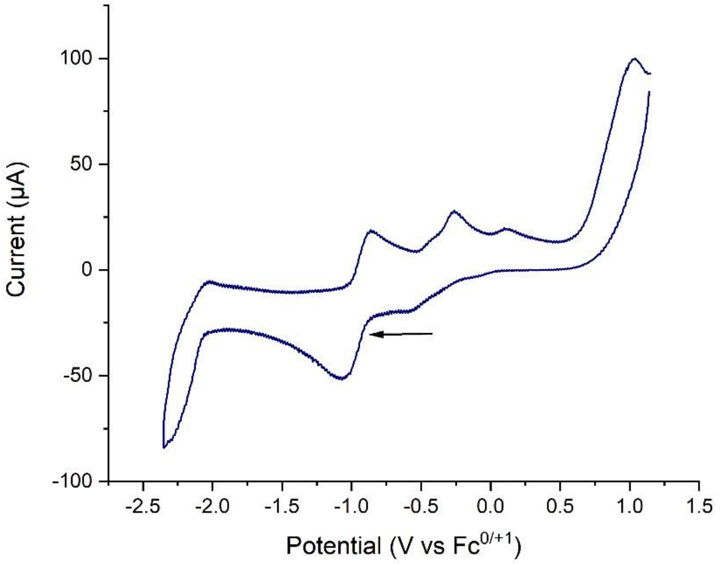

Supplementary Figure S35. Full cyclic voltammogram of **2’** in dichloromethane using 0.10 M [nBu$_4$N][BArF] as supporting electrolyte at ambient temperature; scan rate: 100 mV/s, referenced to $Fc^{0/+}$.

Comment: The main features of the cyclic voltammetry study for **2’** are comparable with the measurement of **2**, supporting the presence of the same Bi_3^+ cation (*cfr*. Supplementary Figure S23).

3.3. Reactivity of **2** as a Bi(I) atom transfer

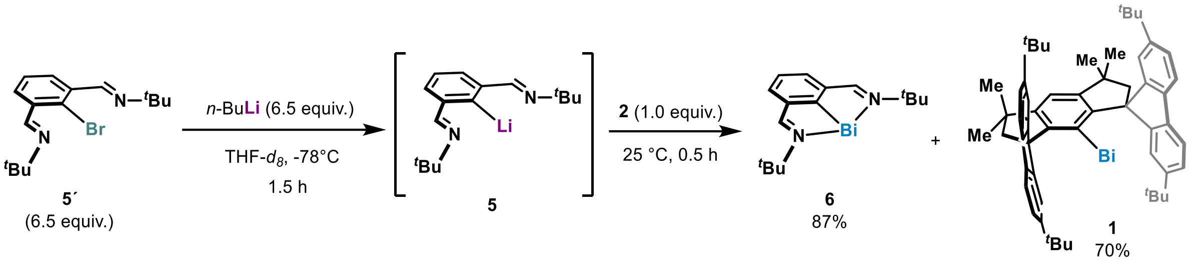

Procedure: In an argon-filled glove box, a stock solution of **5´** is prepared in a Schlenk tube using THF-d_8 as solvent (20.0 mg, 0.0620 mmol, 20 equiv. in 1.00 mL of solvent, 0.0620 M). The tube is then taken out of the glovebox and the solution is cooled to –78 °C. After 15 min stirring, 0.0250 mL of a 2.50 M solution of *n*-BuLi in hexanes (0.0620 mmol, 20.0 equiv.) was added dropwise. The mixture was stirred at this temperature for 1.5 h (the mixture is yellow and homogeneous). After this time, 0.300 mL of the solution containing **5** (6.5 equiv.) is transferred dropwise to a second Schlenk tube containing a mixture of **2** in toluene-d_8 (10.0 mg, 0.00300 mmol, in 0.125 mL). After complete addition, the mixture is stirred for 0.5 h. After this time, the dark green mixture is diluted with a 0.0380 M stock solution of 1,3,5 trimethoxybenzene (0.200 mL, 0.00660 mmol, 2.2 equiv.) and quantitative ^{1}H NMR is measured. The crude mixture shows formation of the desired product in 87% and 70% recovery of **1** (yield normalized to 100%).

HRMS (ESI pos) of crude mixture: calc’d for $C_{16}H_{23}Bi_1N_2^+$ $[M]^+$ 452.16599; found 452.16597.

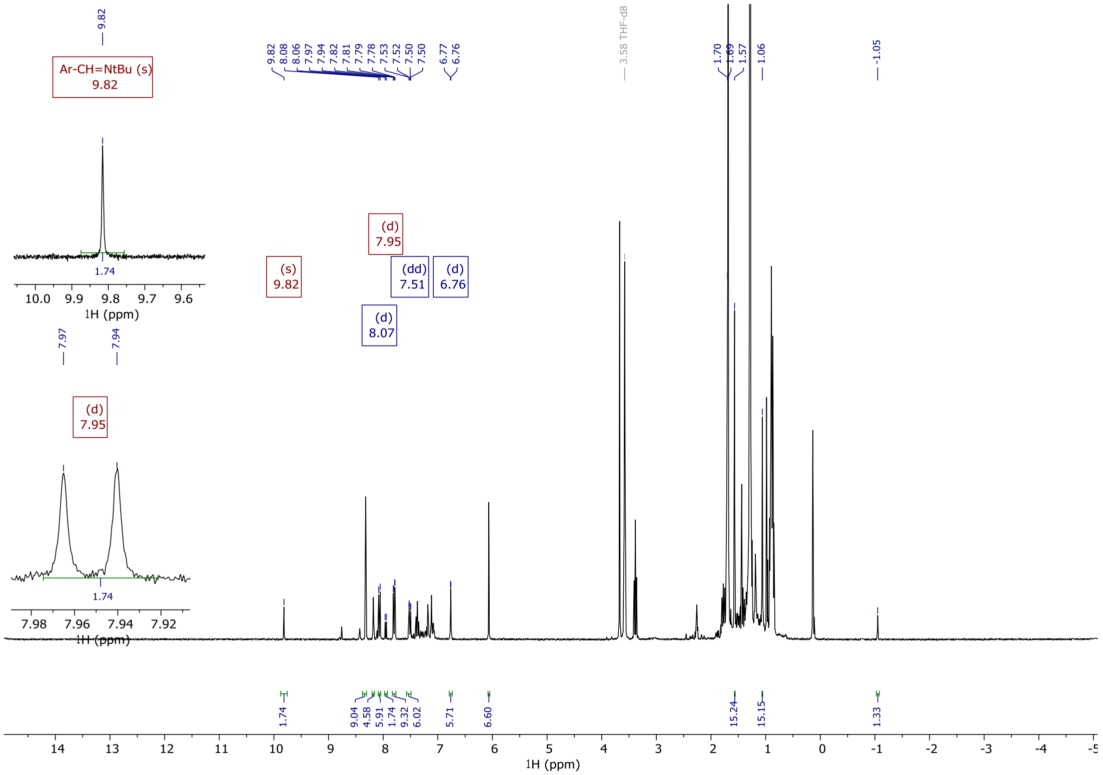

Supplementary Figure S36. Crude ^{1}H NMR spectrum (THF-d_8, 300 MHz, 298 K).

Reported ^{1}H NMR for **6**: ^{1}H NMR (300 MHz, THF-d_8) δ 9.81 (s, 2H), 7.94 (d, *J* = 7.4 Hz, 2H), 7.08 (t, *J* = 7.4 Hz, 1H), 1.57 (s, 18H).[4]

Reported ^{1}H NMR for **1** (600 MHz, THF-d_8, 298 K): δ 8.05 (dd, *J* = 7.8, 0.5 Hz, 4H), 7.50 (dd, *J* = 7.9, 1.9 Hz,4H), 6.73 (dd, *J* = 1.9, 0.5 Hz, 4H), 1.69 (s, 4H), 1.67 (s, 36H), 1.04 (s, 12H), –1.06 ppm (s, 1H).[2]

3.4. Reactivity of **2’** as a Bi(I) atom transfer: N,C,N pincer Bi(I) synthesis

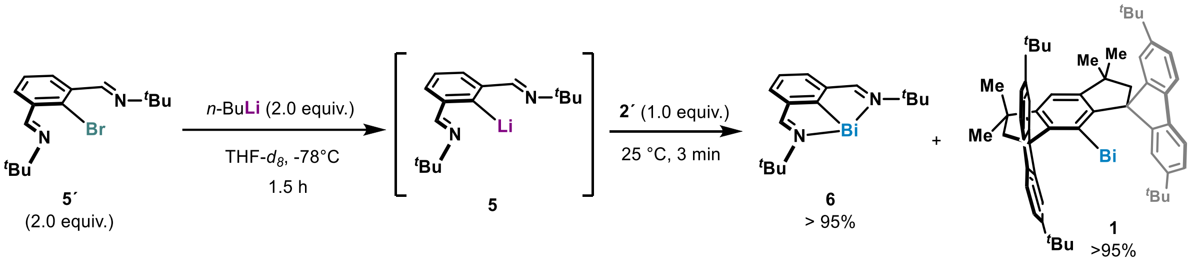

Procedure: In an argon-filled glove box, a stock solution of **5´** is prepared in a Schlenk tube using THF-d_8 as solvent (20.0 mg, 0.0620 mmol, 20 equiv. in 1.00 mL of solvent, 0.0620 M). The tube is then taken out of the glovebox and the solution is cooled to –78 °C. After 15 min stirring, 0.0250 mL of a 2.5 M solution of *n*-BuLi in hexanes (0.0620 mmol, 20 equiv.) was added dropwise. The mixture was left stirring at this temperature for 1.5 h (the mixture is yellow and homogeneous). After this time, 0.10 mL of the solution containing **5** (2.00 equiv.) is transferred dropwise onto a second Schlenk tube containing a mixture of **2’** in toluene-d_8 (9.00 mg, 0.00300 mmol, in 0.125 mL). After complete addition, the mixture is stirred for 3 min. After this time, the dark green mixture is diluted with a THF-d_8, 1,3,5 trimethoxybenzene (3.00 mg, 0.0180 mmol, 6.0 equiv.) is added and ^{1}H NMR is measured. The crude mixture shows formation of the desired product **6** and **1** in quantitative yield (yield normalized to 100% for **1**).

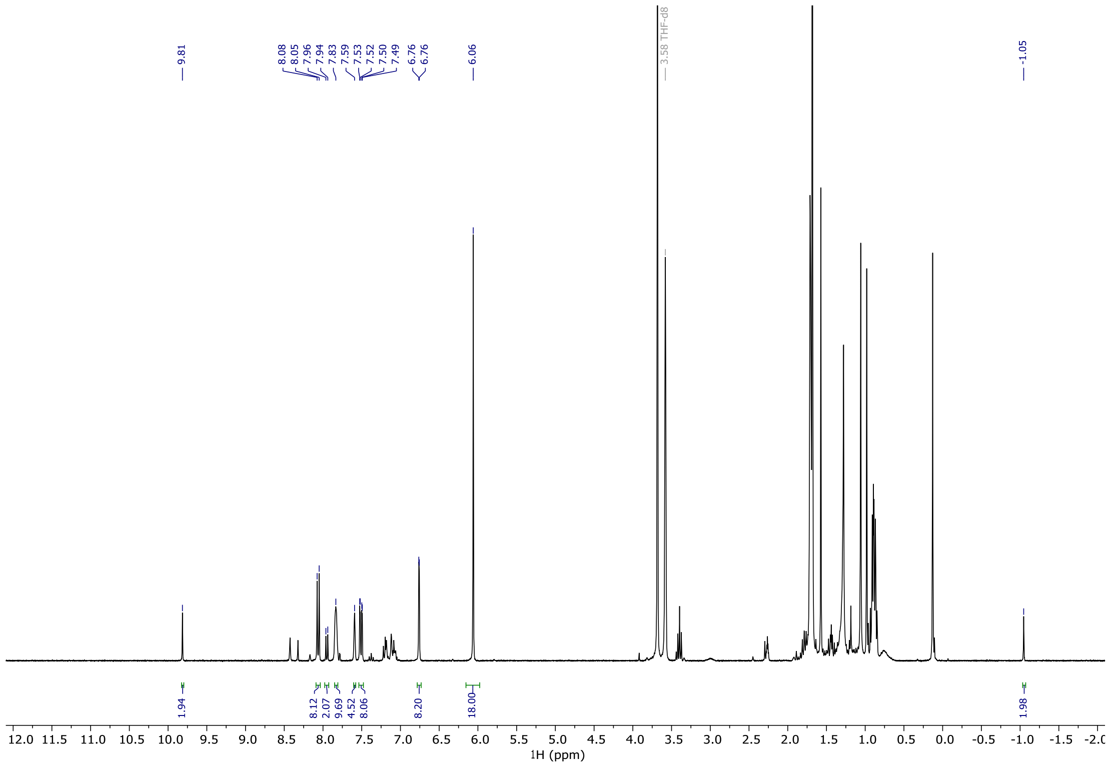

Supplementary Figure S37. Crude ^{1}H NMR spectrum (THF-d_8, 300 MHz, 298 K).

Reported ^{1}H NMR for **6**: ^{1}H NMR (300 MHz, THF-*d*$_8$) δ 9.81 (s, 2H), 7.94 (d, J = 7.4 Hz, 2H), 7.08 (t, J = 7.4 Hz, 1H), 1.57 (s, 18H).[3]

Reported ^{1}H NMR for **1** (600 MHz, THF-*d*$_8$, 298 K): δ 8.05 (dd, J = 7.8, 0.5 Hz, 4H), 7.50 (dd, J = 7.9, 1.9 Hz,4H), 6.73 (dd, J = 1.9, 0.5 Hz, 4H), 1.69 (s, 4H), 1.67 (s, 36H), 1.04 (s, 12H), –1.06 ppm (s, 1H).[2]

3.5. Reactivity of **2'** as a Bi(I) atom transfer: N,N,N pincer Bi(I) synthesis

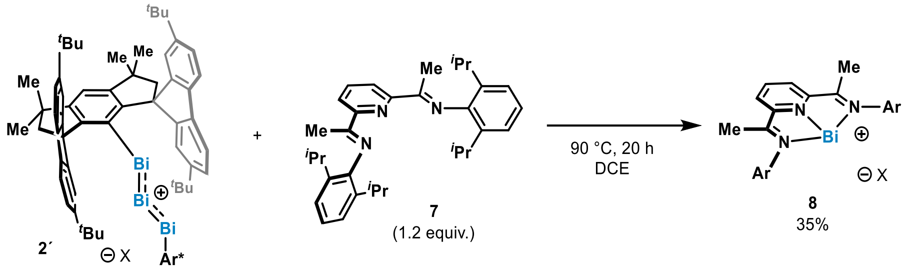

Procedure: In an argon-filled glove box, an oven dried Schlenk tube is charged with **2'** (15 mg, 0.0044 mmol, 1 equiv.), and **7** (2.5 mg, 0.0052 mmol, 1.2 equiv). Finally, anhydrous 1,2 dichloroethane (0.22 mL) is added and the tube is placed in an oil bath at 90 °C. After 20 h, the mixture is cooled to 25 °C and the volatiles are evaporated under high vacuum. The crude material is treated under an inert atmosphere with a 1:1 mixture of toluene/pentane (3mL in total) and the blue liquid phase is place into a vial at -35 °C degrees. After 10 d blue crystals of **8** could be obtained in 35% yield (2.4 mg, 0.0020 mmol) after washing the material with anhydrous pentane and drying it under high vacuum. In this reaction, however, **1** cannot be recovered since it undergoes oxidation with 1,2-dichloroethane delivering Ar*$BiCl_2$ and ethylene. In order to confirm the formation of Ar*$BiCl_2$, an authentic sample of **1** was heated at 90 °C in 1,2-dichloroethane, thus obtaining Ar*$BiCl_2$ and ethylene (*vide infra*).

Characterization data of **8**:

^{1}H NMR (600 MHz, DCM-*d*$_2$, 298 K) δ 9.11 (d, *J* = 7.8 Hz, 2H, H-2), 7.74 – 7.69 (m, 8H, H-202), 7.55 (s, 4H, H-204), 7.43 – 7.35 (m, 6H, H-8 & H-9), 7.07 (t, *J* = 7.9 Hz, 1H, H-1), 3.99 (s, 6H, H-5), 2.42 (hept, *J* = 6.9 Hz, 4H, H-10), 1.17 (d, *J* = 6.9 Hz, 12H, H-11), 1.09 (d, *J* = 6.8 Hz, 12H, H-12).

^{13}C NMR (151 MHz, DCM-*d*$_2$, 298 K) δ 168.8 (C-4), 162.1 (q, *J* = 49.8 Hz, C-201), 154.8 (C-3), 141.7 (C-7), 138.4 (C-1), 135.9 (C-6), 135.6 – 134.5 (m, C-202), 129.5 (C-9), 129.3 (qq, *J* = 31.5, 3.0 Hz, C-203), 125.0 (q, *J* = 272.4 Hz, C-205), 124.8 (C-8), 118.0 – 117.8 (m, C-204), 29.4 (C-10), 25.6 (C-12), 24.0 (C-11), 19.7 (C-5).

^{11}B (192 MHz, DCM-*d*$_2$, 298 K) δ -6.7.

^{19}F NMR (282 MHz, DCM-*d*$_2$, 298 K) -62.8.

M.p.: the compound melts at 280°C in a flame-sealed argon-filled capillary.

HRMS (ESI pos): calc'd for $C_{33}H_{43}Bi_1N_3^+$ $[M]^+$ 690.32552; found 690.32555.

Stability: air sensitive.

XRD: single crystals of **8** suitable for X-ray diffraction analysis were obtained by dissolving the blue solid in a toluene-pentane mixture and placing the sample at -35° C for 10 d.

Anal. calc'd for **8•** toluene ($C_{72}H_{63}BBiF_{24}N_3$): C, 52.54; H, 3.86; B, 0.66; Bi, 12.70; F, 27.70; N, 2.55; found: C, 52.46; H, 3.84; B, 0.67; Bi, 12.67; F, 27.64; N, 2.53.

MIKROLAB
Mikroanalytisches Laboratorium Kolbe

Davide Spinnato

Max-Planck-Institut für Kohlenforschung
Dr. Josep Cornellà
Nachhaltige Katalyse für die Organische Synthese

Anschrift : Osterfelder Str. 3
D-46047 Oberhausen
Telefon : +49 - (0)208 - 32502
Telefax : +49 - (0)208 - 382314
Email : info@mikro-lab.de
Webseite : www.mikro-lab.de

Datum : 30.08.2024

Probenbezeichnung	% C	% H	% N	% B	% F	% Bi	% Dry				Argon	V2O5
SPD - SA - 492 - 00	52,46	3,84	2,53	0,67	27,64	12,67	0,12				x	x

Mit freundlichen Grüssen

Patrick Springer

Nuclear magnetic resonance spectroscopy (NMR) spectra

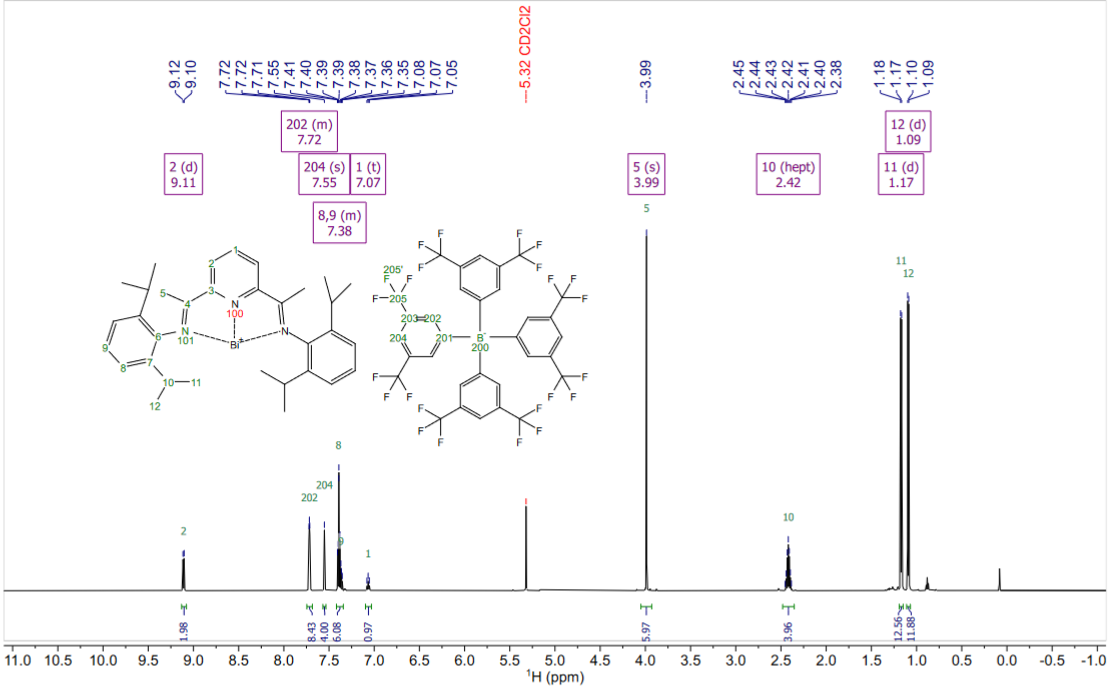

Supplementary Figure S38. ^{1}H NMR spectrum of **8** (DCM-d_2, 600 MHz, 298 K).

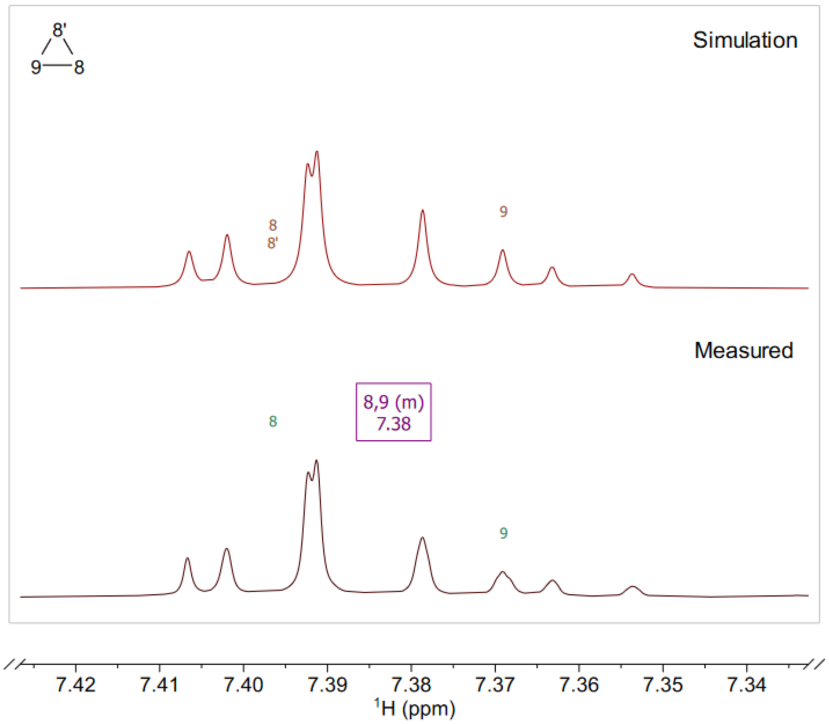

Spin Simulation

#	Label	Value	N	Spin	LineWidth (Hz)
1	8'	7.40 ppm	1	1/2	0.8
	J8'-8	1.60 Hz			
	J8'-9	7.80 Hz			
2	8	7.40 ppm	1	1/2	0.8
	J8-8'	1.60 Hz			
	J8-9	7.80 Hz			
3	9	7.37 ppm	1	1/2	0.8
	J9-8'	7.80 Hz			
	J9-8	7.80 Hz			

Supplementary Figure S39. S Simulated vs measured multiplet of protons #8,8´, and 9 in **8** (DCM-d_2, 600 MHz, 298 K).

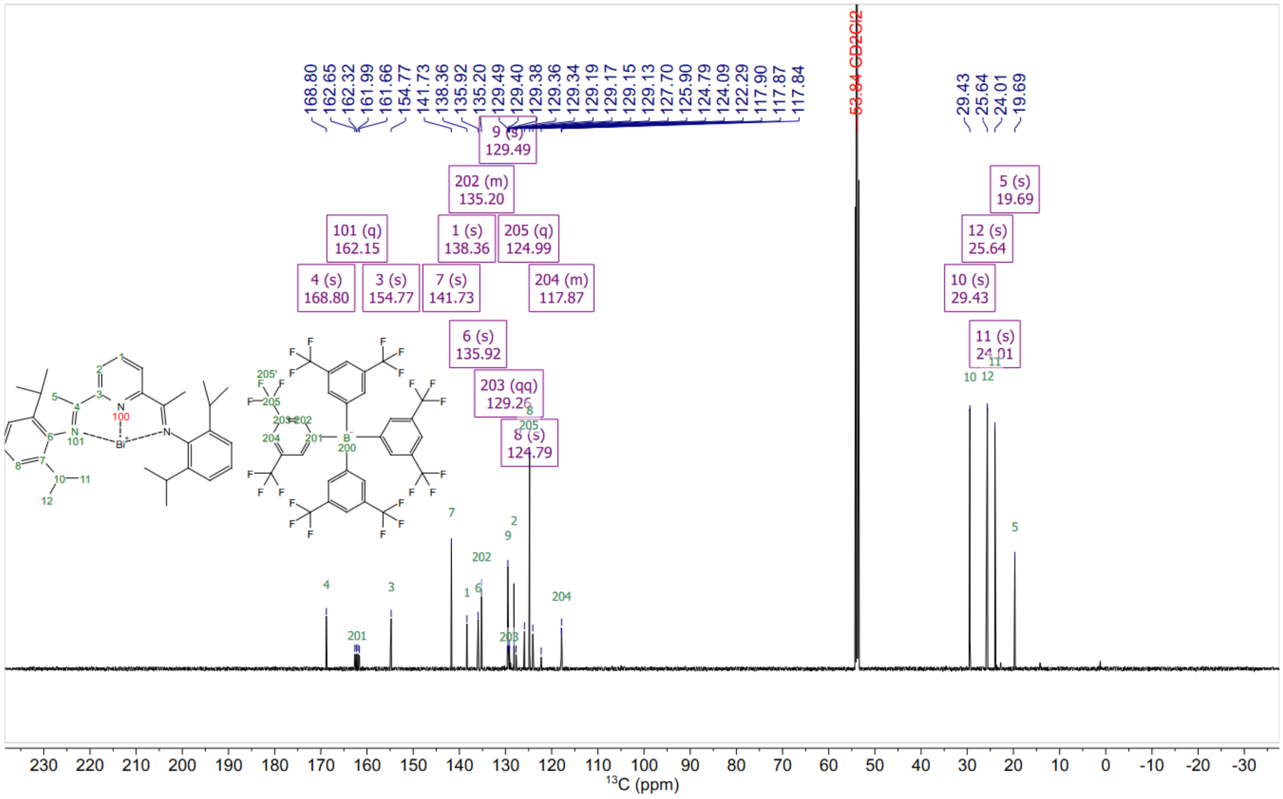

Supplementary Figure S40 ^{13}C NMR spectrum of **8** (DCM-d_2, 151 MHz, 298 K).

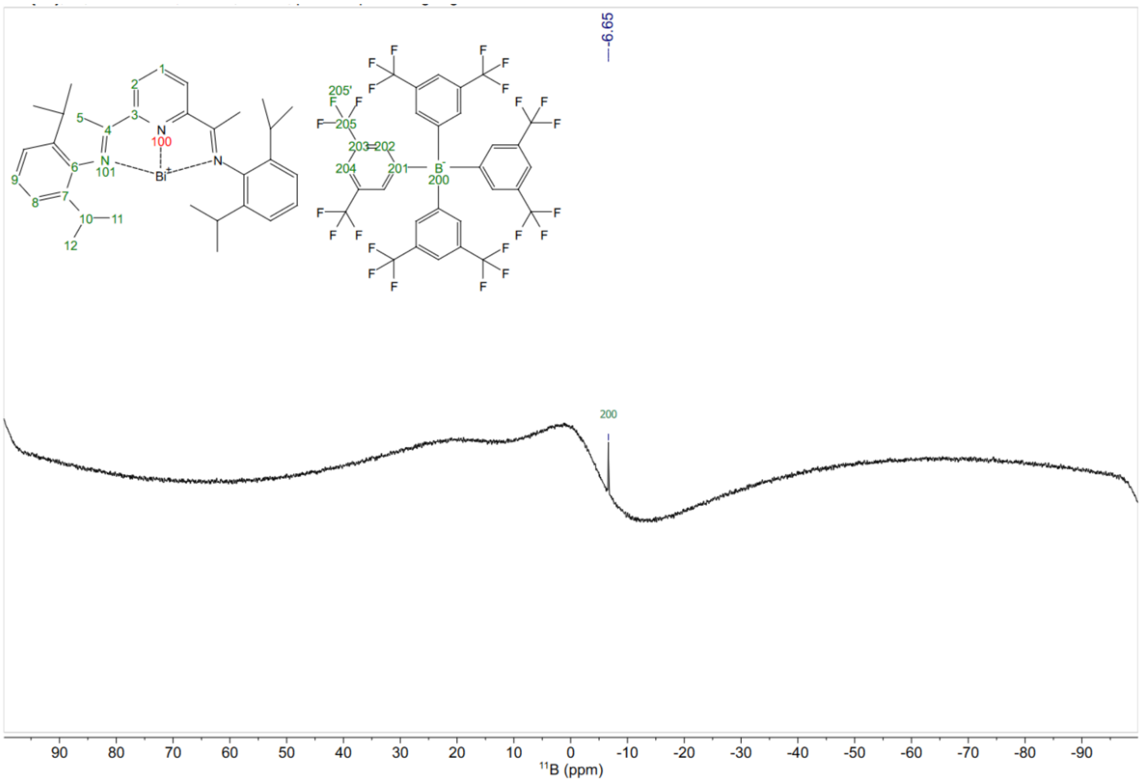

Supplementary Figure S41. ^{11}B NMR spectrum of **8** (DCM-d_2, 192 MHz, 298 K).

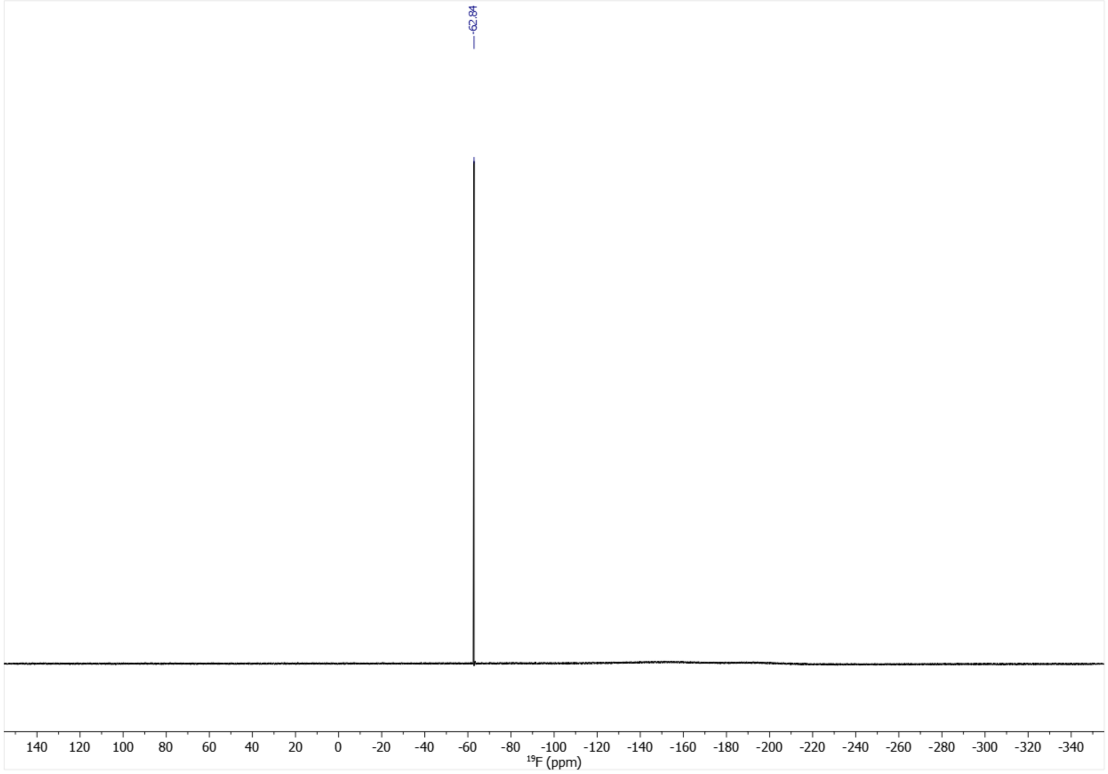

Supplementary Figure S42. ^{19}F NMR spectrum of **8** (DCM-d_2, 282 MHz, 298 K).

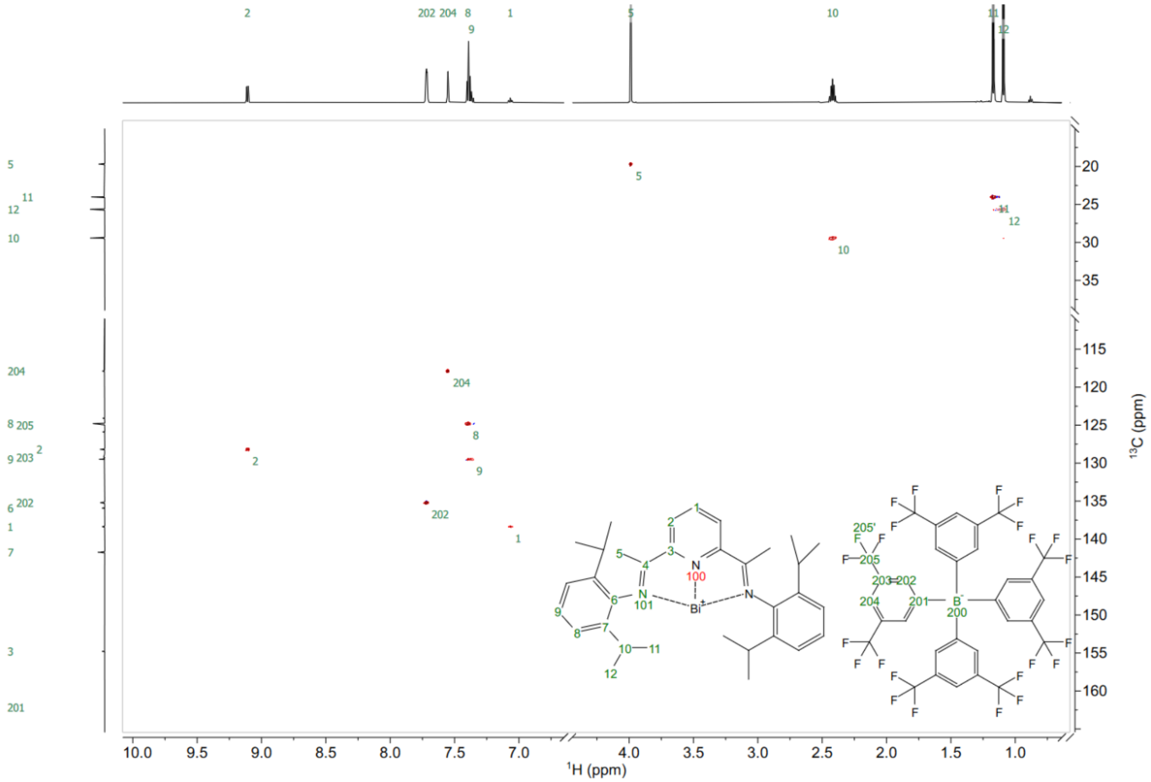

Supplementary Figure S43. ^{1}H-^{13}C edited HSQC spectrum of **8** (DCM-d_2, 600 MHz, 298 K).

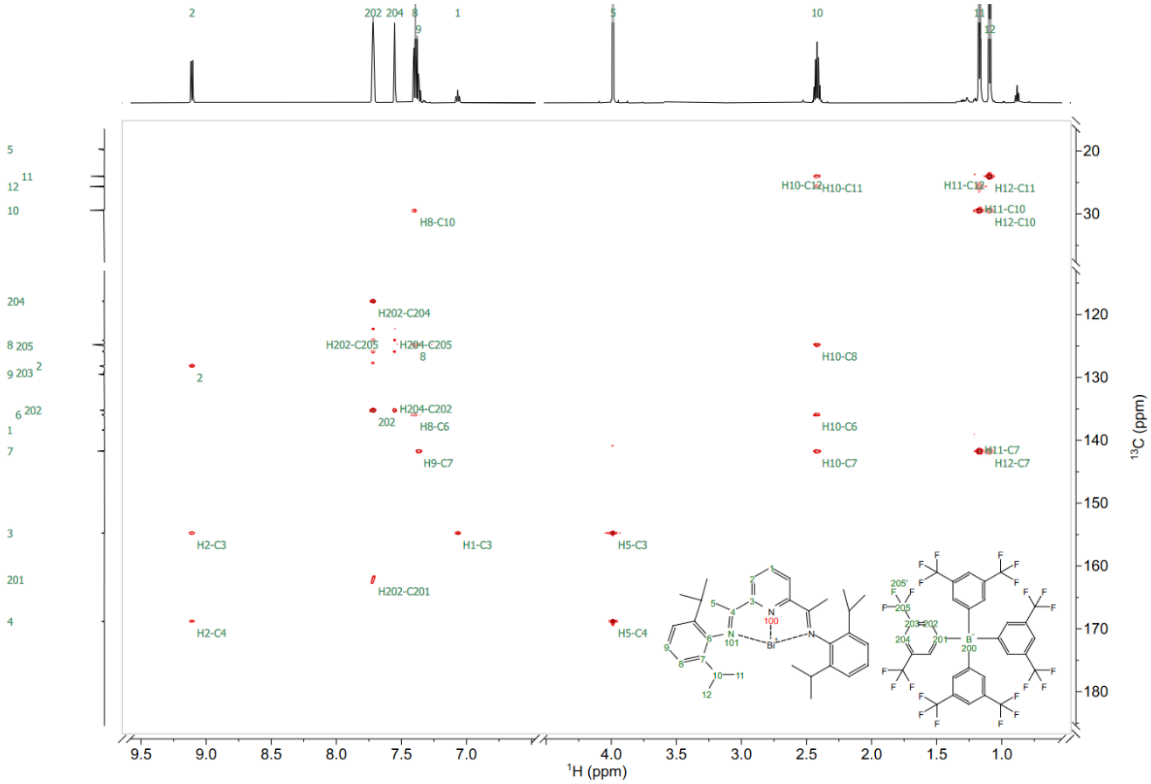

Supplementary Figure S44. ^{1}H-^{13}C HMBC NMR spectrum of **8** (DCM-d_2, 600 MHz, 298 K).

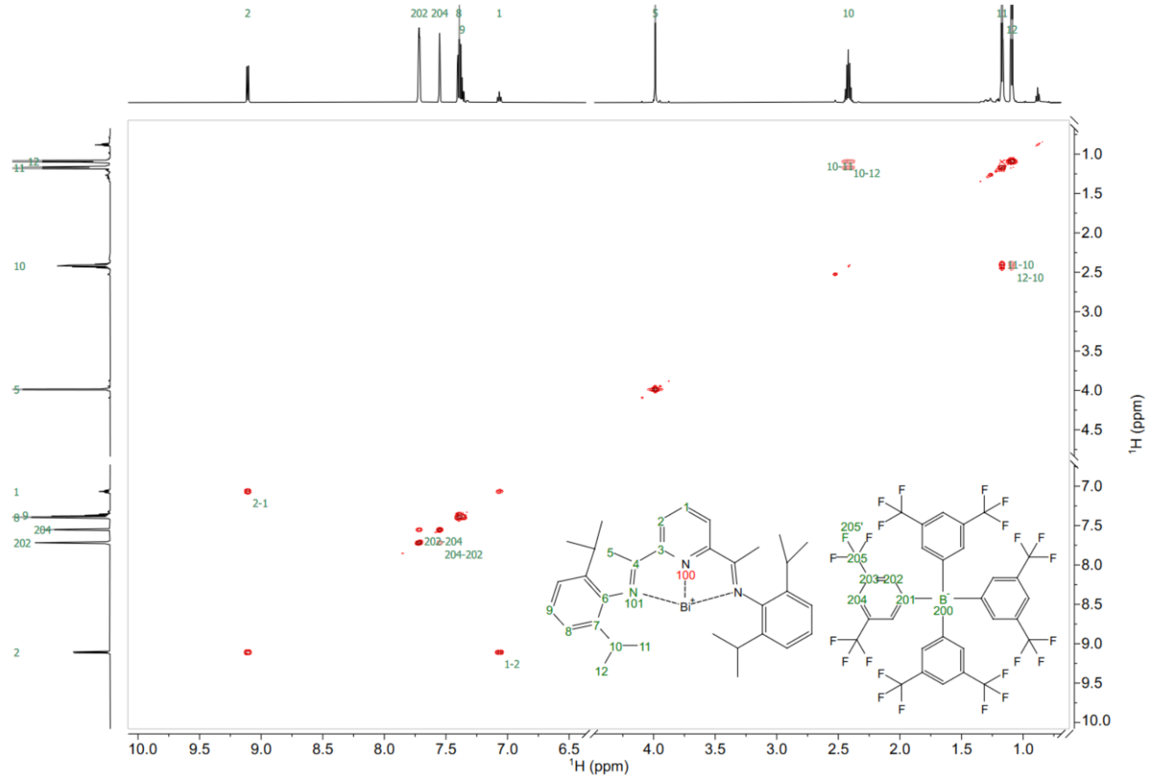

Supplementary Figure S45. ^{1}H-^{1}H COSY NMR spectrum of **8** (DCM-d_2, 600 MHz, 298 K).

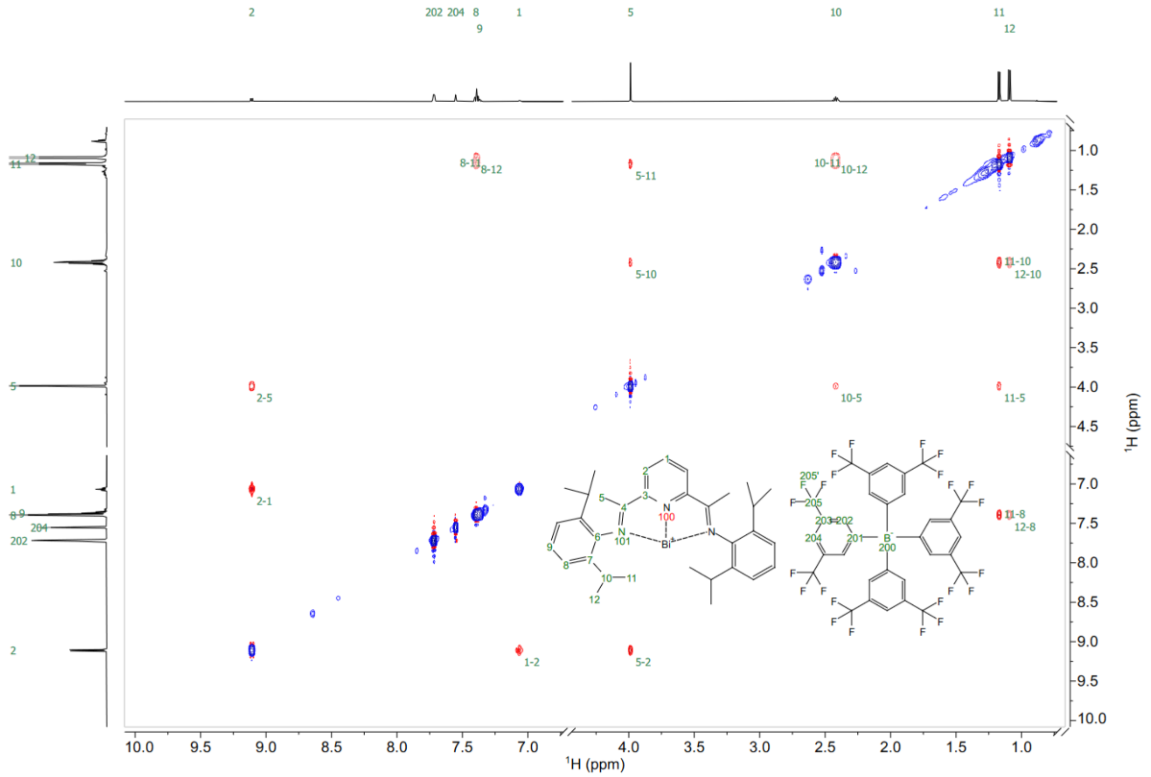

Supplementary Figure S46. ^{1}H-^{1}H NOESY spectrum of **8** (DCM-d_2, 600 MHz, 298 K).

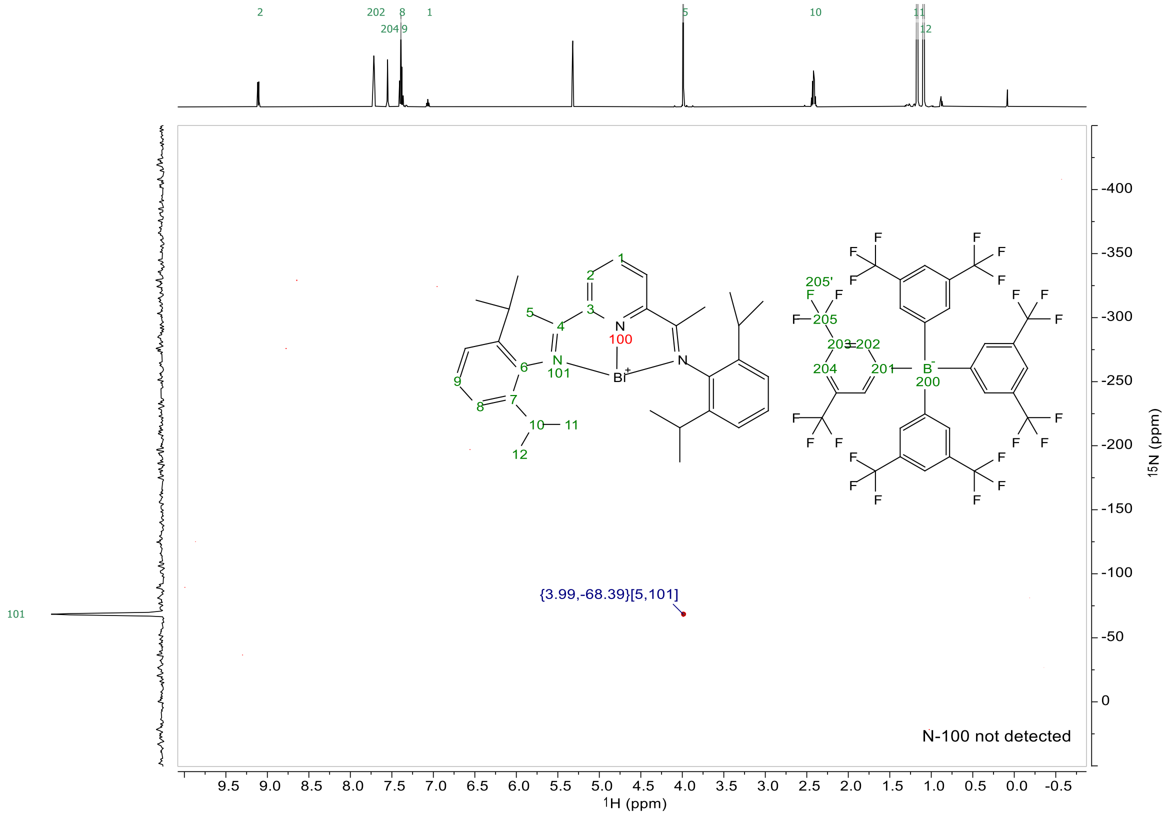

Supplementary Figure S47. ^{1}H-^{15}N HMBC spectrum of **8** (DCM-d_2, 600 MHz, 298 K).

*Evidences for the formation of Ar*BiCl$_2$ from* ***1*** *under transfer conditions:*

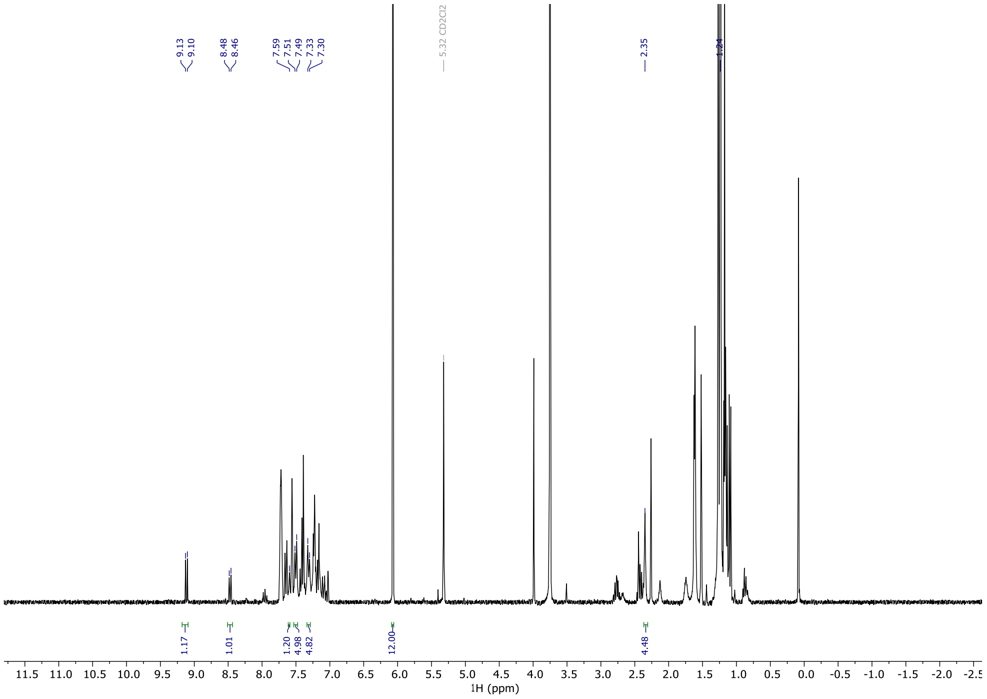

Supplementary Figure S48. ^{1}H NMR spectrum of crude mixture of transfer reaction towards **8** (DCM-d_2, 300 MHz, 298 K).

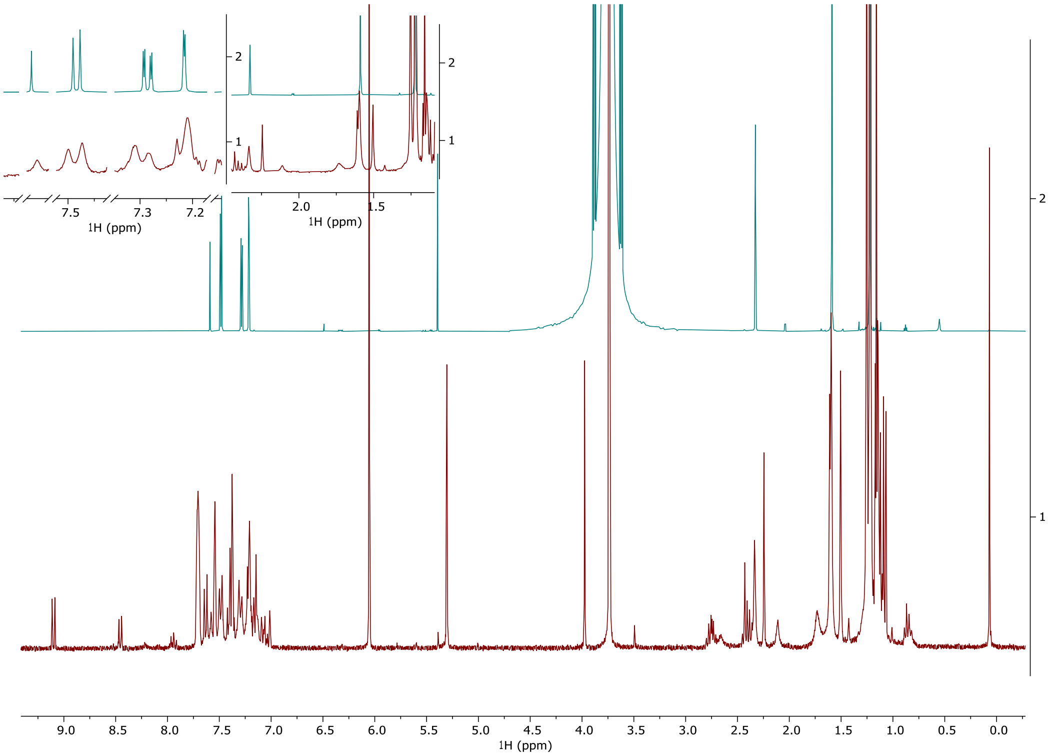

Supplementary Figure S49. Stacked ^{1}H NMR spectra of crude mixtures. Green spectrum: crude mixture of the reaction of **1** with 1,2-dichloroethane at 90° C. Red spectrum: crude mixture of the transfer reaction towards **8** (DCM-d_2, 300 MHz, 298 K).

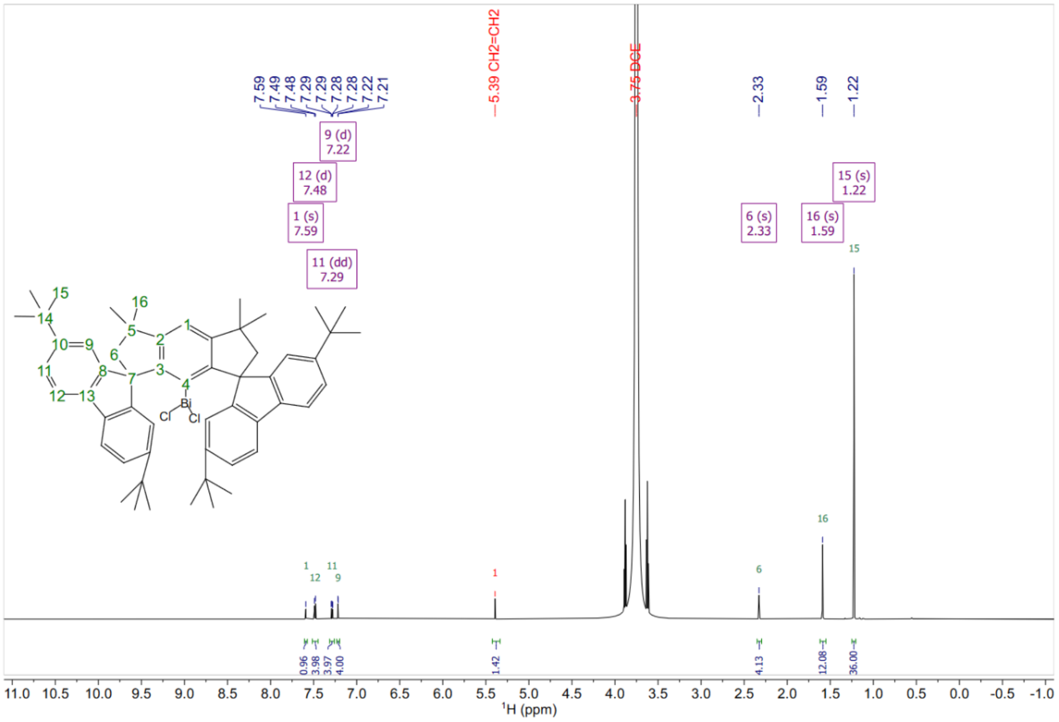

Supplementary Figure S50. ^{1}H NMR spectrum of crude reaction mixture of the reaction of **1** with 1,2-dichloroethane at 90 °C (1,2-dichloroethane-h_4, 600 MHz, 298 K).

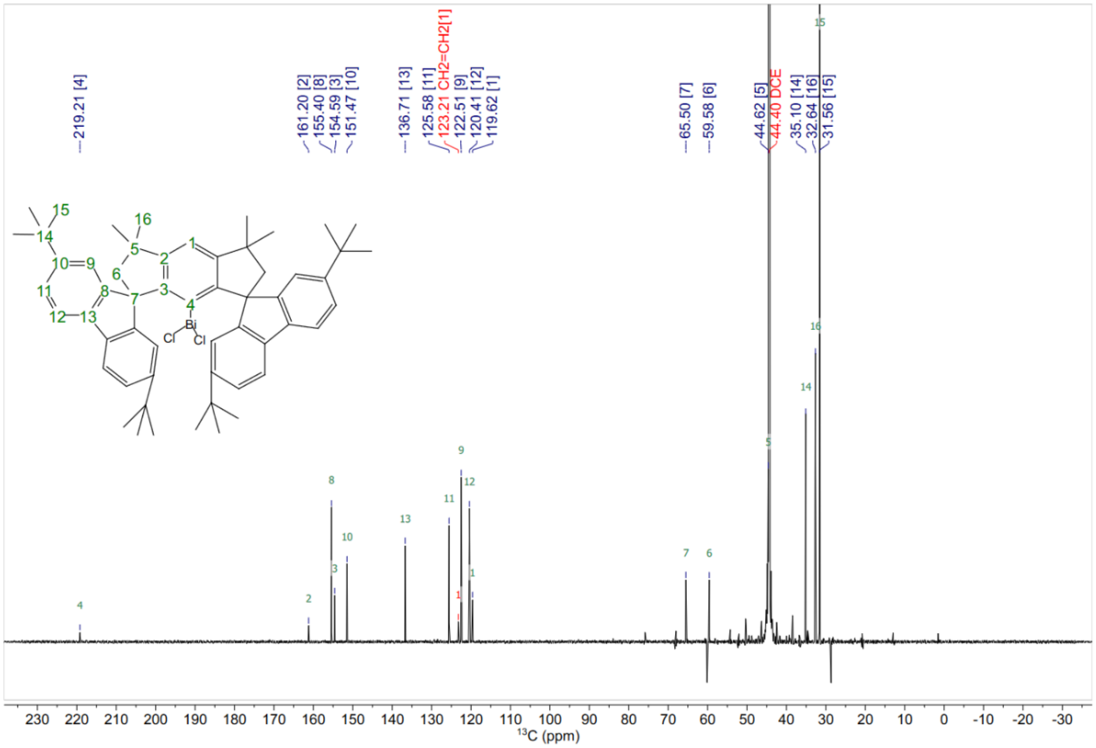

Supplementary Figure S51. ^{13}C NMR spectrum of the crude mixture of the reaction of **1** with 1,2-dichloroethane at 90 °C (1,2-dichloroethane-h_4, 600 MHz, 298 K). Symmetric artefacts around the solvent peak occurred due to broadband broadband decoupling.

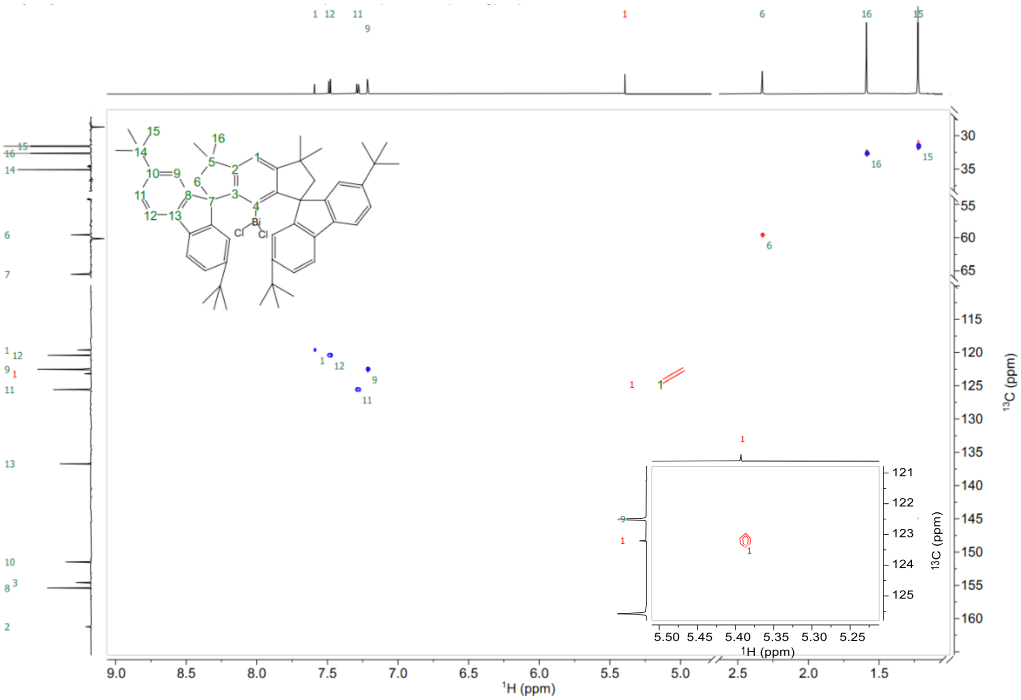

Supplementary Figure S52. ^{1}H-^{13}C edited HSQC spectrum of crude mixture of the reaction of **1** with 1,2-dichloroethane at 90 °C, (1,2-dichloroethane-h_4, 600 MHz, 298 K).

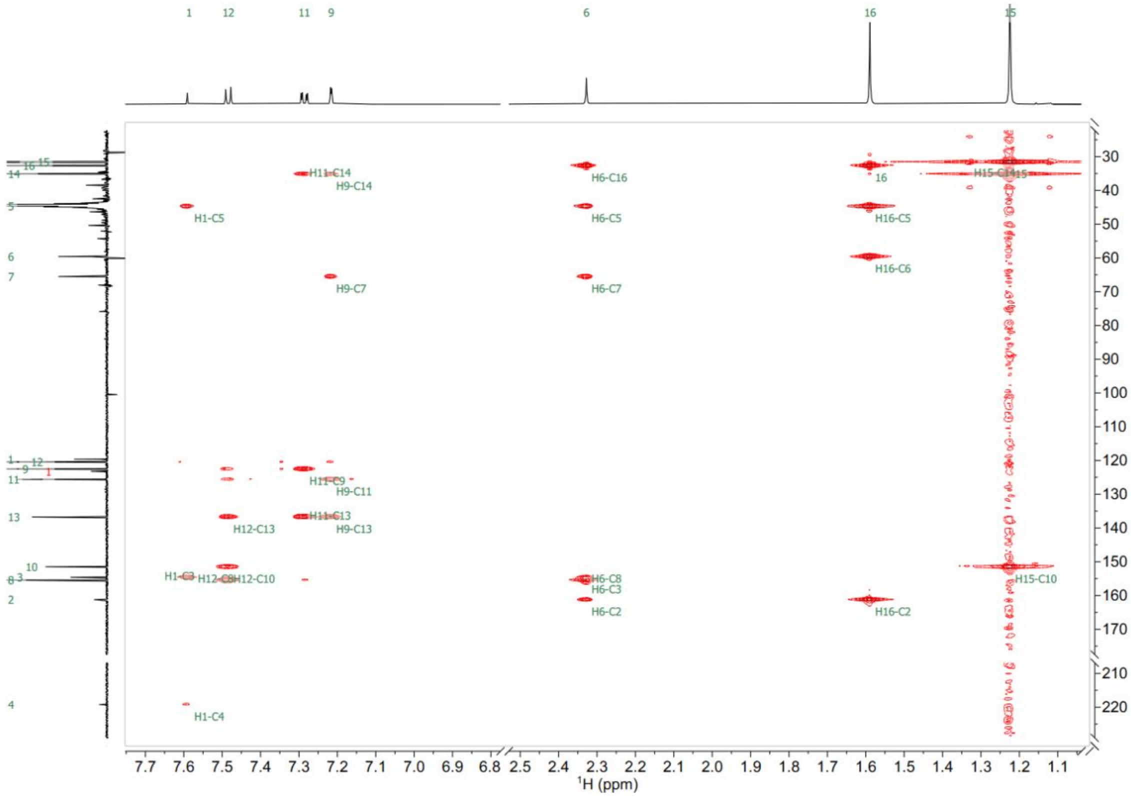

Supplementary Figure S53. ^{1}H-^{13}C HMBC spectrum of the crude mixture of the reaction of **1** with 1,2-dichloroethane at 90 °C (1,2-dichloroethane-h_4, 600 MHz, 298 K).

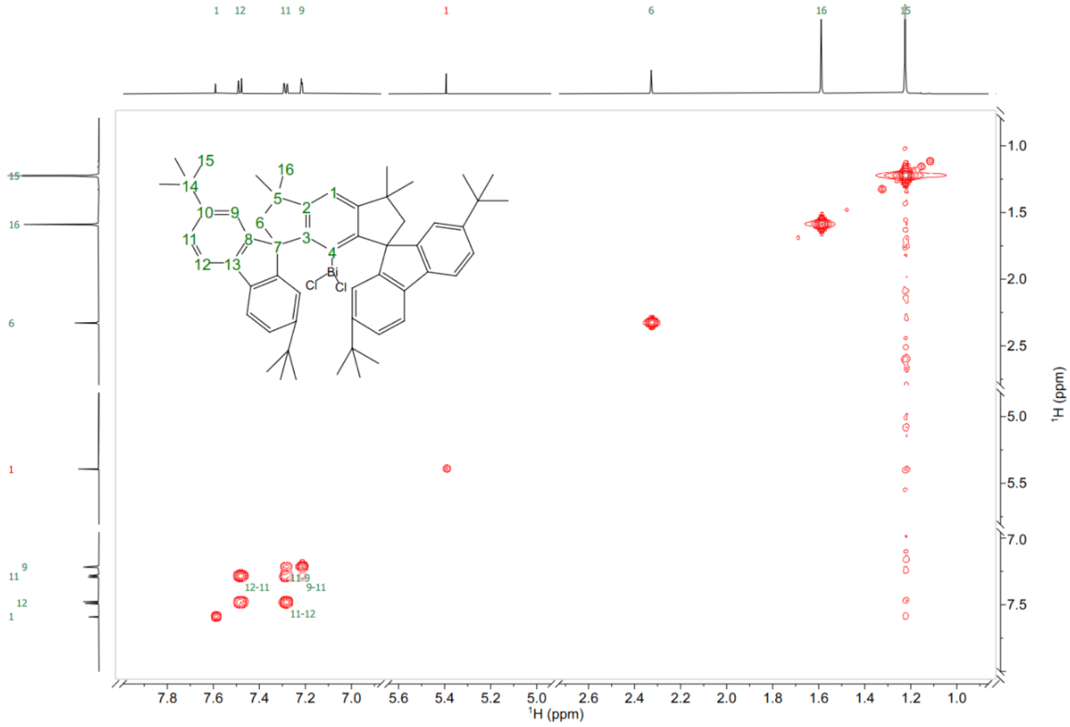

Supplementary Figure S54. ^{1}H-^{1}H COSY spectrum of the crude mixture of the reaction of **1** with 1,2-dichloroethane at 90 °C (1,2-dichloroethane-h_4, 600 MHz, 298 K).

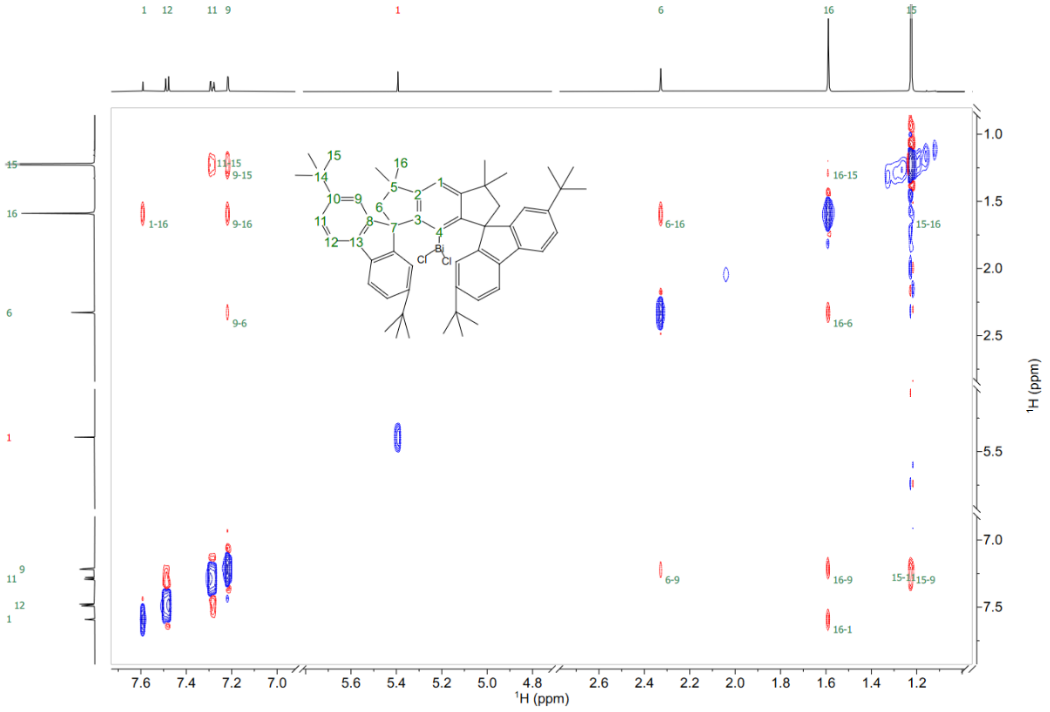

Supplementary Figure S55. ^1H-^1H NOESY spectrum of the crude mixture of the reaction of **1** with 1,2-dichloroethane at 90 °C (1,2-dichloroethane-h_4, 600 MHz, 298 K).

Overall, the spectroscopic data agrees well with previously reported data of similar compounds.[1]
HRMS (ESI pos): calc'd for $C_{56}H_{65}Bi_1Cl_2$ $[Na]^+$ 1039.41599; found 1039.41595.

4. X-ray Crystal Structure Analyses

4.1. Single crystal structure analysis of **2**• 2 toluene-d_8

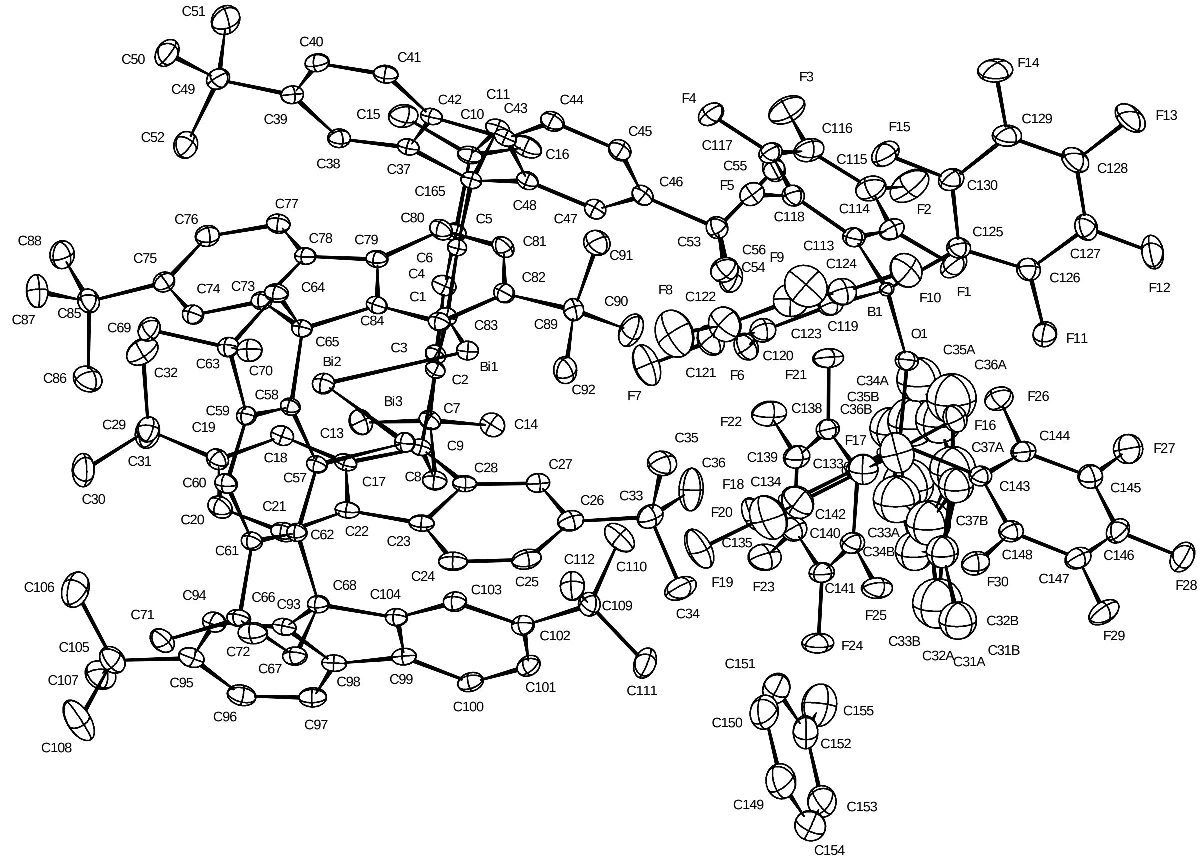

Supplementary Figure S56. ORTEP for structure**2** · 2 toluene-d_8 solvate; thermal ellipsoids at 50% probability level. H and D atoms have been removed for clarity.

X-ray Crystal Structure Analysis of complex **2** · 2 toluene-d_8 solvate:

C_{162} H_{131} B_2 Bi_3 D_{16} F_{30} O, M_r = 3336.40 g mol^{-1}, red plate, crystal size 0.419 x 0.23 x 0.05 mm^3, triclinic, space group *P*-1 [2], a = 11.9290(3) Å, b = 22.8775(5) Å, c = 28.7042(7) Å, α = 112.7350(10)°, β = 94.5760(10)°, γ = 96.3540(10)°, V = 7116.0(3) Å^3, T = 100(2) K, Z = 2, D_{calc} = 1.557 g·cm^3, λ = 0.71073 Å, $\mu(Mo\text{-}K_{\alpha})$ = 3.793 mm^{-1}, Numerical correction ($T_{\min}$ = 0.3829, $T_{\max}$ = 0.9763), Bruker-AXS D8 Venture with Photon III detector and IµS Diamond microfocus Mo-anode X-ray source, 1.992 < θ < 33.142°, 1313320 measured reflections, 54258 independent reflections, 46812 reflections with $I > 2\sigma(I)$, R_{int} = 0.0759. The structure was solved by *SHELXT* and refined by full-matrix least-squares (*SHELXL*) against F^2 to R_1 = 0.0246 [$I > 2\sigma(I)$], wR_2 = 0.0572 [all data], 1886 parameters and 356 restraints.[5-8]

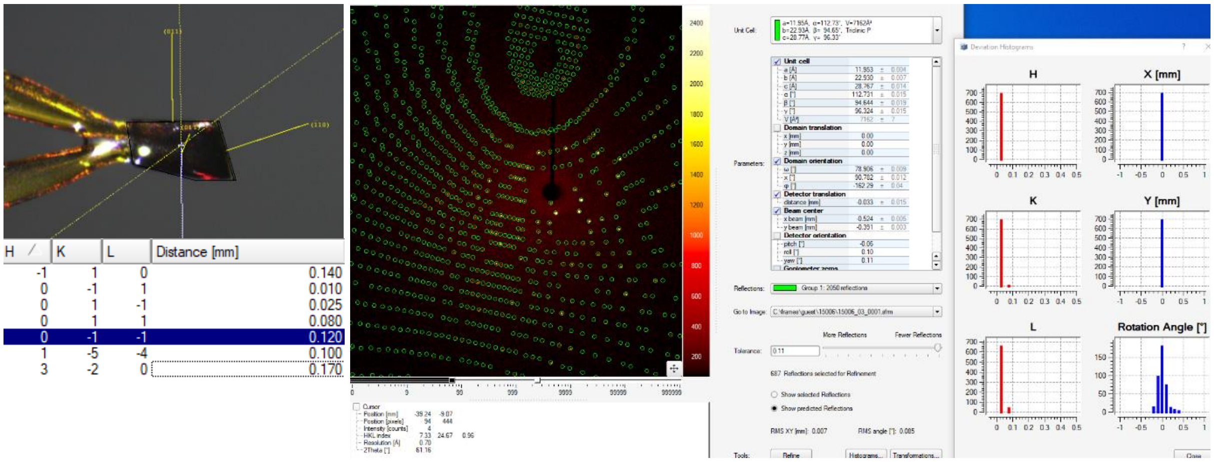

Supplementary Figure S57. Crystal faces and unit cell determination/refinement of complex **2** · 2 toluene-d_8 solvate.

INTENSITY STATISTICS FOR DATASET

Resolution	#Data	#Theory	%Complete	Redundancy	Mean I	Mean I/s	Rmerge	Rsigma
Inf - 2.50	941	954	98.6	30.95	77.68	70.61	0.0322	0.0267
2.50 - 1.67	2249	2251	99.9	37.94	52.11	73.77	0.0331	0.0139
1.67 - 1.33	3111	3112	100.0	39.20	35.69	66.45	0.0409	0.0114
1.33 - 1.16	3246	3246	100.0	37.92	29.60	58.42	0.0511	0.0121
1.16 - 1.05	3319	3319	100.0	31.70	21.89	46.02	0.0644	0.0152
1.05 - 0.98	2954	2954	100.0	28.30	17.25	38.55	0.0779	0.0184
0.98 - 0.92	3253	3253	100.0	26.15	16.19	34.26	0.0850	0.0205
0.92 - 0.87	3543	3543	100.0	24.60	14.46	30.81	0.0949	0.0231
0.87 - 0.84	2517	2517	100.0	23.26	12.26	26.56	0.1085	0.0269
0.84 - 0.80	3949	3949	100.0	22.07	10.14	22.64	0.1261	0.0319
0.80 - 0.78	2302	2302	100.0	21.56	8.92	20.60	0.1413	0.0361
0.78 - 0.75	3940	3940	100.0	20.00	8.16	17.94	0.1479	0.0411
0.75 - 0.73	2990	2990	100.0	18.33	7.93	16.40	0.1567	0.0459
0.73 - 0.71	3310	3310	100.0	17.56	7.38	14.90	0.1657	0.0504
0.71 - 0.69	3726	3726	100.0	17.16	6.51	13.60	0.1838	0.0576
0.69 - 0.68	2075	2075	100.0	16.69	5.98	12.28	0.1996	0.0640
0.68 - 0.66	4403	4403	100.0	15.77	5.09	10.16	0.2311	0.0788
0.66 - 0.65	2436	2436	100.0	15.31	4.92	9.51	0.2403	0.0843
0.65 - 0.64	2579	2579	100.0	15.04	4.22	8.18	0.2696	0.0984
0.64 - 0.62	5870	6163	95.2	11.21	3.95	6.62	0.2851	0.1350
0.72 - 0.62	22793	23086	98.7	14.85	5.15	10.03	0.2224	0.0835
Inf - 0.62	62713	63022	99.5	22.55	14.38	26.99	0.0775	0.0288

Complete .cif-data are available under the CCDC number CCDC-**2336523**.

Alert B was found: PLAT910_ALERT_3_B Missing # of FCF Reflection(s) Below Theta(Min). Author response: Some low index reflexes were slightly affected by the beam stop and were excluded before the final refinement cycles.

Some low index reflections were slightly affected by the beam stop and were excluded before the final refinement cycles. A resolution cut-off was applied to exclude incompletely observed shells at high

diffraction angles. The structure contains a disordered d8-toluene molecule. It was described using the 'toluene' fragment from the FragmentDB implemented in Olex2-1.5.[5-8] The occupancy was freely refined and coupled with an FVAR. This resulted in a refined occupancy of 51.457:48.453% for the respective parts. Anisotropic deflection parameters combined with the ISOR instruction were applied to the disordered solute molecule.The hydrogen atom on the diboroxanium was found in the residual electron density and freely refined at this point. After insertion of the hydrogen atom, the residual electron density at this position decreased to an acceptably flat value.

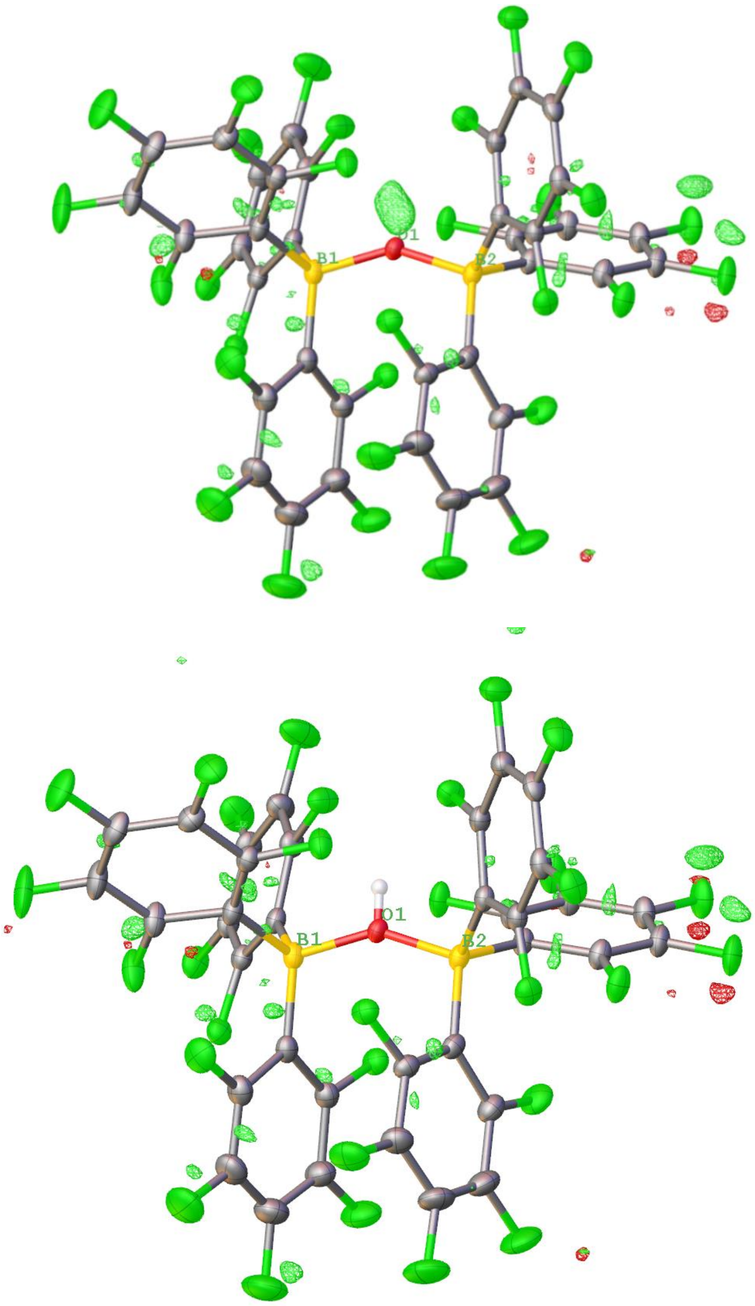

Supplementary Figure S58. Residual electron density map (source: Olex2-1.5, diff-map, level 0.5 e/Å^3, 0.1 Å resolution) of the anion of compound **2** without a H atom attached to O1 (top) and with a H atom at O1 (bottom).

Supplementary Table S1. Crystal data and structure refinement of **2** · 2 toluene-d_8 solvate.

Identification code	15006	
Empirical formula	C_{162} H_{139} D_8 B_2 Bi_3 F_{30} O	
Color	red	
Formula weight	3336.40 g·mol^{-1}	
Temperature	100(2) K	
Wavelength	0.71073 Å	
Crystal system	triclinic	
Space group	*P*-1, (no. 2)	
Unit cell dimensions	a = 11.9290(3) Å	α= 112.7350(10)°.
	b = 22.8775(5) Å	β= 94.5760(10)°.
	c = 28.7042(7) Å	γ = 96.3540(10)°.
Volume	7116.0(3) Å^3	
Z	2	
Density (calculated)	1.557 Mg·m^{-3}	
Absorption coefficient	3.793 mm^{-1}	
F(000)	3312 e	
Crystal size	0.419 x 0.23 x 0.05 mm^3	
θ range for data collection	1.992 to 33.142°.	
Index ranges	$-18 \le h \le 18$, $-35 \le k \le 35$, $-44 \le l \le 44$	
Reflections collected	1313320	
Independent reflections	54258 [R_{int} = 0.0759]	
Reflections with I>2σ(I)	46818	
Completeness to θ = 25.242°	99.9 %	
Absorption correction	Numerical	
Max. and min. transmission	0.9763 and 0.3829	
Refinement method	Full-matrix least-squares on F^2	
Data / restraints / parameters	54258 / 356 / 1886	
Goodness-of-fit on F^2	1.027	
Final R indices [I>2σ(I)]	R_1 = 0.0246	wR^2 = 0.0534
R indices (all data)	R_1 = 0.0343	wR^2 = 0.0572
Extinction coefficient	n/a	
Largest diff. peak and hole	1.394 and -0.985 e·Å^{-3}	

4.2 Structure search in the Cambridge Structural Database (CSD)

A database (ConQuest Version 2023.2.0; CSD Version 5.44. April 2023 + 1 update Jun 2023) survey was performed on 01st March 2024 to search for related structures of acyclic Bi-Bi and Bi=Bi bonds. The following search motifs were used:

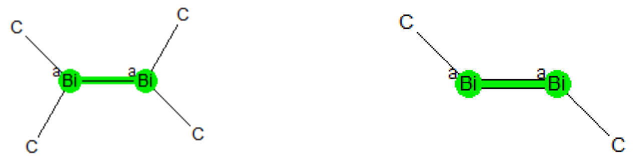

Supplementary Figure S59. Search motif for data survey on related structures of acyclic Bi single bonds (left) and Bi double bonds (right).

The initial search found 13 structures containing the predefined Bi-Bi motif. Furthermore, 9 structures containing the predefined Bi=Bi motif where found. The individual bond lengths of the symmetry independent molecules in the crystal structures were used for the statistical analysis.

Supplementary Table S2. Overview of search results for Bi-Bi motif.

CSD-Refcode	Bi-Bi distance / Å	R_1-factor / %	Publication Year
PERTIT	2.980	3.80	2023
BOZVER01	2.983	3.94	1992
GAVKIZ	2.983	2.00	2011
ODAMEL	2.983	6.20	2001
ODAMAH	2.984	3.40	2001
BOZVER02	2.987	3.64	1992
BOZVER11	2.987	5.55	2021
BOZVER	2.990	5.55	1983
BOZVER10	2.990	3.64	1984
BOZVER11	2.996	5.77	2021
HAHHUX	3.001	1.42	2021
ENIVUV	3.020	5.84	2021
EGEQEM	3.054	2.42	2002
FIYXUH	3.087	3.80	2005

The calculated mean value of the Bi-Bi bond lengths in the found structures is 3.002(32) Å.

Supplementary Table S3. Overview of search results for Bi=Bi motif.

CSD-Refcode	Bi-Bi distance / Å	R_1-factor / %	Publication Year
EKUJAY	2.821	5.56	2021
RETTIT	2.821	4.44	1997
RETCOK	2.831	2.59	2013
CIGVIX	2.833	5.27	1999
GEZHIG	2.847	3.90	2022
WATYIC	2.854	3.15	2017
HOBMUH	2.856	2.54	2008
IFOXEG	2.870	3.99	2002

VUSVEM	2.873	5.12	2015
EKUJAY	2.821	5.56	2021
RETTIT	2.821	4.44	1997
RETCOK	2.831	2.59	2013
CIGVIX	2.833	5.27	1999
GEZHIG	2.847	3.90	2022

The calculated mean value of the Bi-Bi bond lengths in the found structures is 2.845(20) Å.

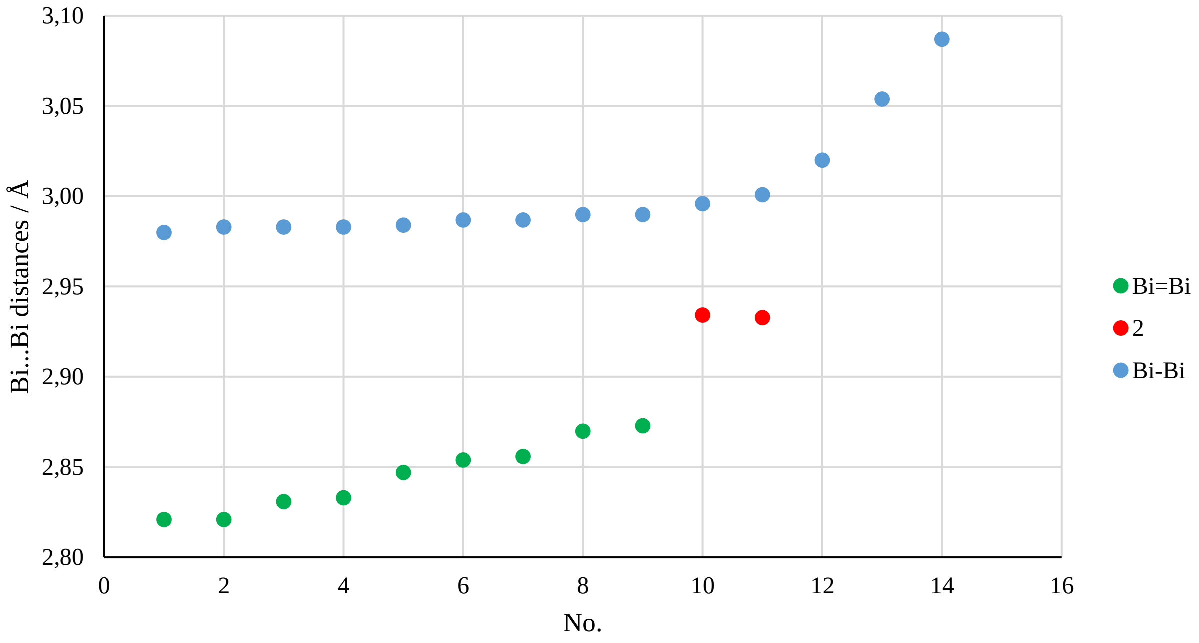

Supplementary Figure S60. Graphical representation of the results of the database search for different Bi-Bi and Bi=Bi bonds. The Bi=Bi distances are shown in green, the Bi-Bi distance in blue and the two distances from **2** in red.

The mean Bi…Bi distance determined in **2** (2.93361(95) Å) is intermediate between the distances of classical and acyclic Bi-Bi (3.002(32) Å) and Bi=Bi (2.845(20) Å) bonds.

A further database (ConQuest Version 2023.2.0; CSD Version 5.44. April 2023 + 1 update Jun 2023) survey was performed on 01st March 2024 to search for related structures of complex diboroxanium counterion. The following search motif was used:

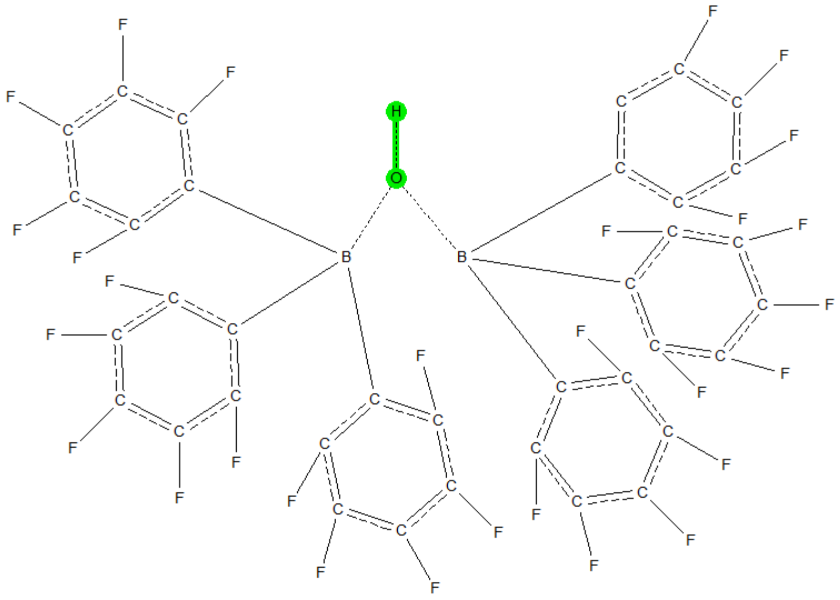

Supplementary Figure S61. Search motif for data survey on related structures of diboroxanium counterions.

The initial search resulted 14 structures containing the predefined molecular motif.

Supplementary Table S4. Overview of search results for the complex diboroxanium counterions.

CSD-Refcode	B(1)-O distance / Å	B(2)-O distance / Å	O-H distance / Å	R_1-factor / %	Publication Year
HUCMAT	1.551	1.566	0.573	4.17	2001
IGOGIX	1.566	1.556	0.621	4.31	2019
MERDOC	1.563	1.558	0.732	5.61	2001
QEHLEX	1.561	1.559	0.759	4.32	2017
TOCFEY	1.561	1.563	0.764	6.13	2014
XABHER	1.557	1.564	0.764	4.39	2020
CUCYUW	1.560	1.567	0.765	3.71	2015
ERIFIU	1.572	1.565	0.781	4.31	2004
TAFSUS	1.570	1.562	0.782	4.32	2020
GIWXEP	1.566	1.563	0.792	5.19	1998
OVINIT	1.576	1.564	0.798	7.60	2021
QEHVEH	1.566	1.565	0.816	7.35	2017
GECHAB	1.565	1.558	0.821	3.54	2022
ITULOA	1.562	1.571	0.878	4.60	2016

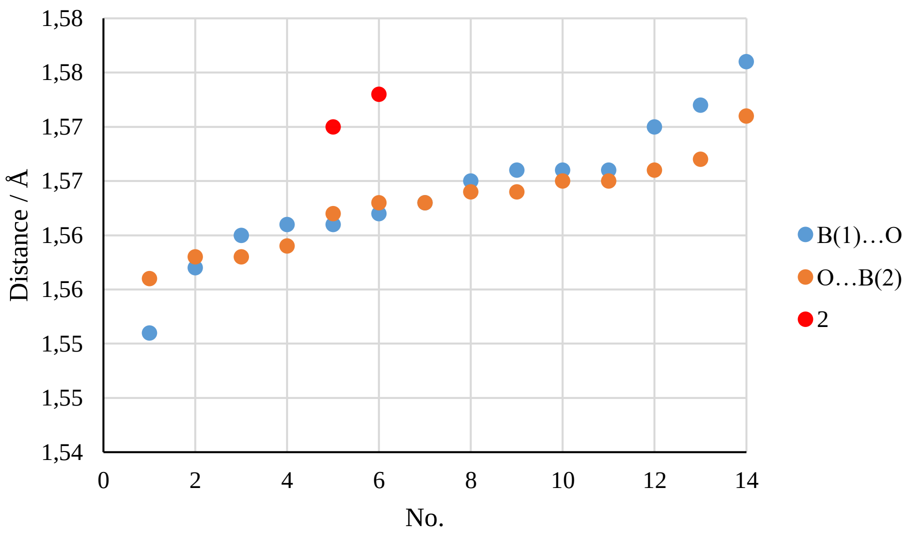

Supplementary Figure S62. Graphical representation of the results of the database search for different diboroxanium counterions. The B…O distances are shown in blue and orange. The distance found in **2** are shown in red.

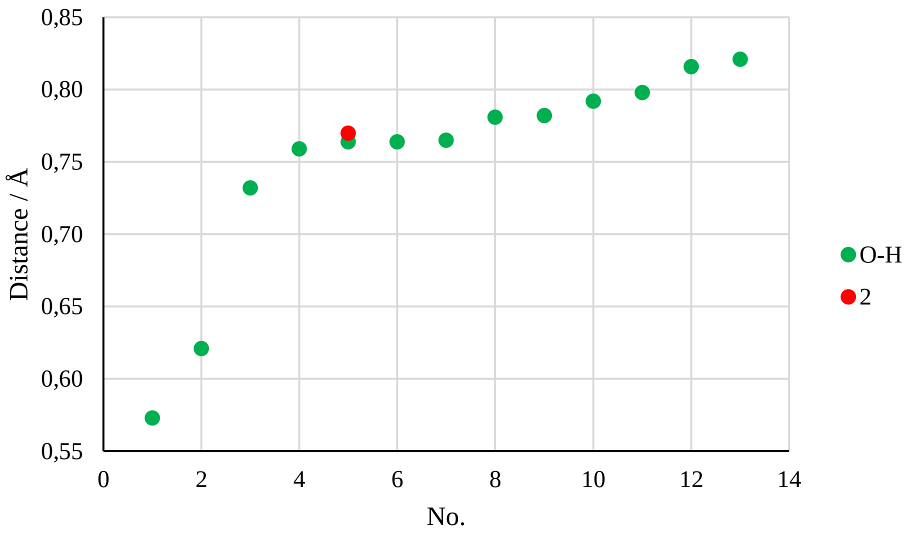

Supplementary Figure S63. Graphical representation of the results of the database search for different diboroxanium counterions. The O-H distances are shown in green and distance found in **2** is shown in red.

4.3 Structure chemical discussions

Compound **2** crystallizes in the triclinic space group *P*-1 (SG No.2) with two units per unit cell. The solid consists of a layered structure with a layer of anions alternating with a layer of cations. At 11.371 Å, the anion is relatively far away from the positive charge of the trimetallic cation.

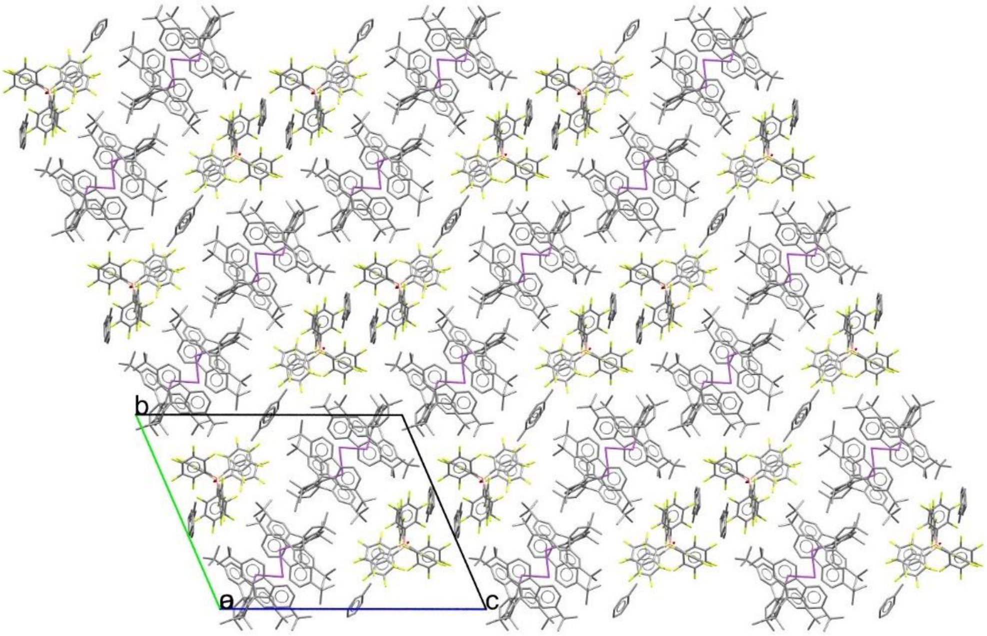

Supplementary Figure S64. Alternating layers of anions and cations in the solid state structure viewed along crystallographic *a* axis.

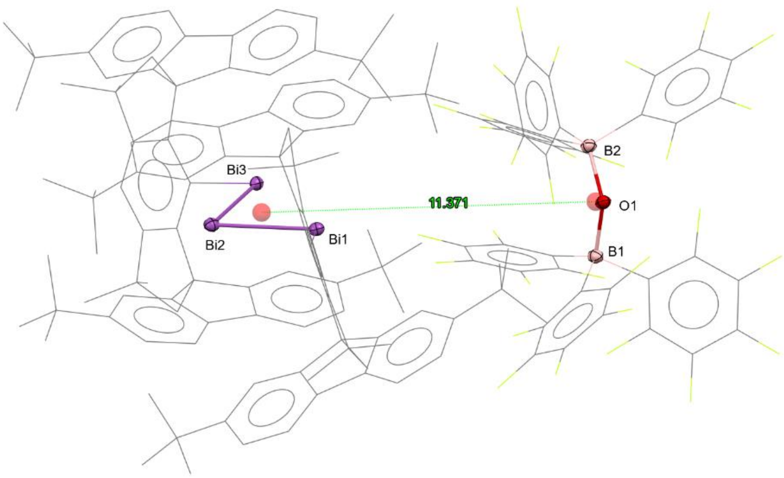

Supplementary Figure S65. The distance between the anion and cation in the crystalline solid. Centroids (red) at the centers of the molecules were chosen as reference points.

In view of the distance of 11.371 Å between the anion and the cation, as well as the steric requirements of the anion, this is probably a weak co-ordination effect. The weakly coordinating anion is capable to stabilize the sensitive bismallyl cation in solid phase.

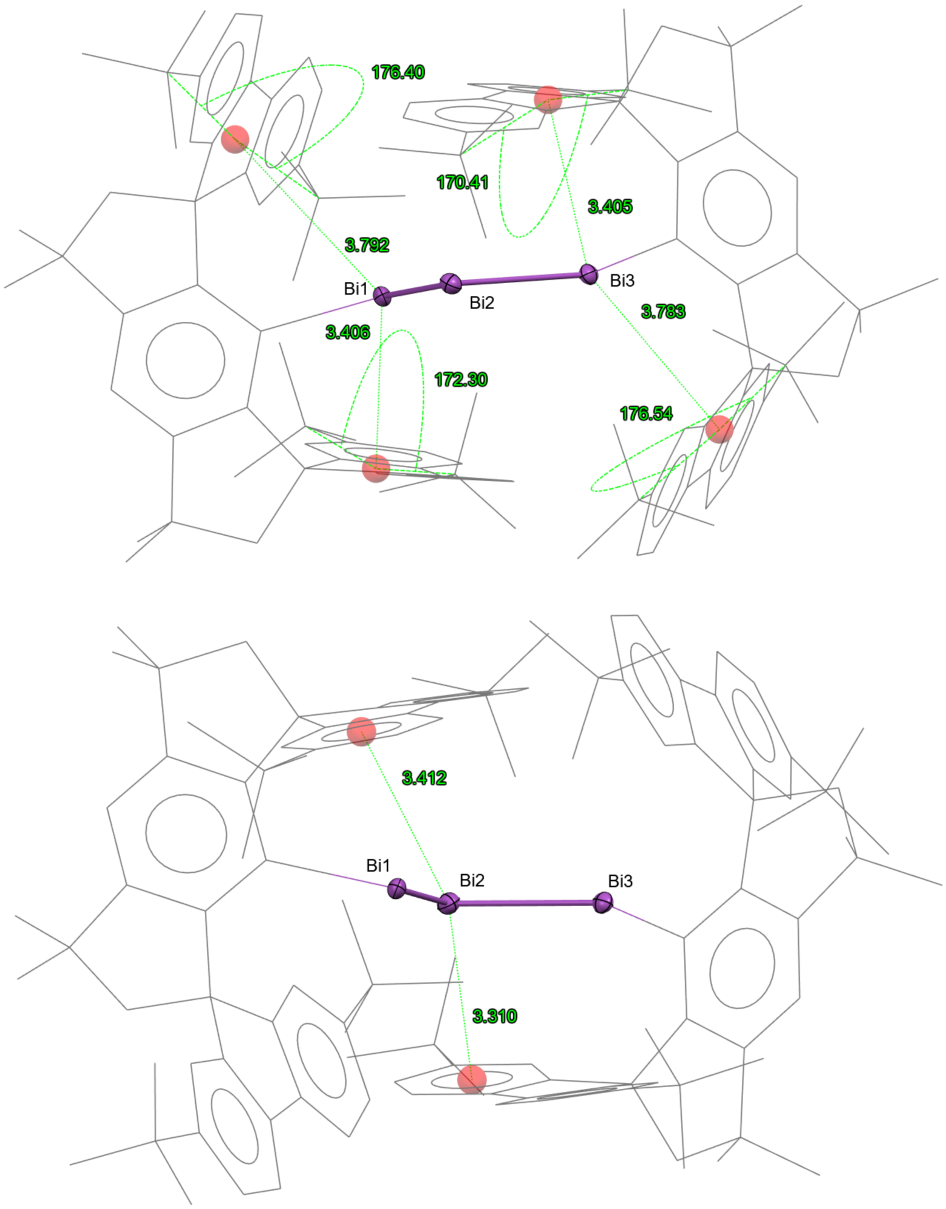

Supplementary Figure S66. Simplified representations of the structure of **2**. The distances between Bi1 or Bi3 and the side arms of the fluorenly ligand as well as the bending angles (top). The interaction of the central Bi2 with the side arms of the fluorenyl ligand (bottom).

A closer look at the structure of the allyl cation reveals further structural-chemical properties of compound **2**. The two terminal Bi1 and Bi3 atoms have relatively short distances (3.406 and 3.405 Å) to a side arm of the fluorene ligands (centroids), respectively. In comparison with the recently described **1** (ref. code: GEZGUR; Bi...centroid distances: 3.353 and 3.449 Å), similar distances to the side arms of the ligand are present in the case of structure **2**. In the study of **1**, significant interactions between the fluorenyl ligand and the central Bi(I) atom were found by theoretical calculations. Due to the two distances of similar length in structure **2**, a similar stabilising intra molecular interaction can be assumed. The other two side arms are further away (3.792 and 3.783 Å) and do not seem to have a stabilising effect to the cationic centre.

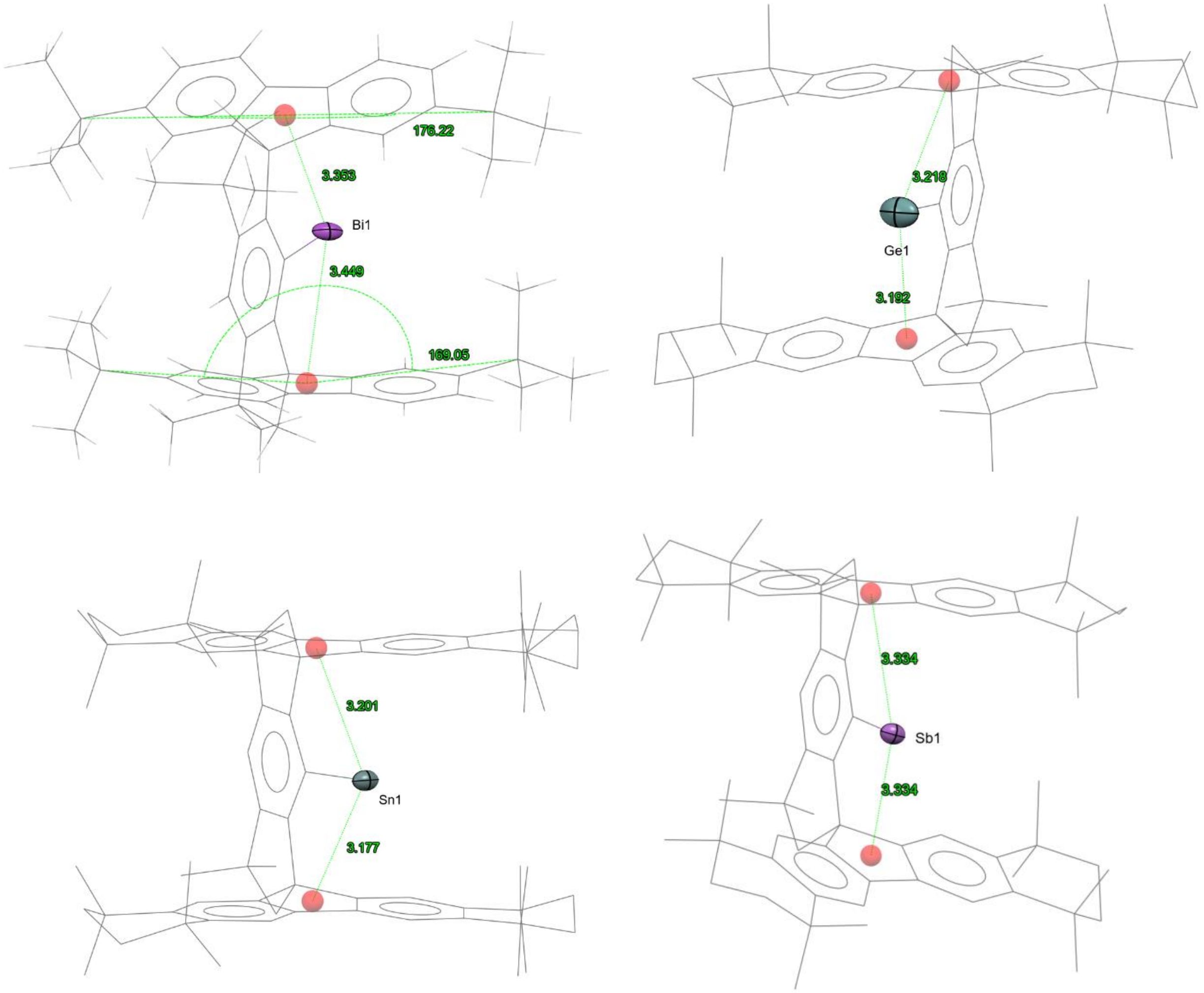

Supplementary Figure S67. Simplified representations of the different singly bounded metal-fluorenyl complexes (ref. codes: GEZGUR top left; VEQMEN top right; HIBNAL bottom left; OFOKED bottom right). The distances between the metal centers and the centroids of the side arms of the fluorenly ligand are shown.

Another interesting point is that the two side arms interacting with Bi1 and Bi3 show a slight bending (172.30 and 170.41°). The other two side arms, which do not interact, are almost planar (176.40 and 176.54°) as one would expect. It is also noticeable that the central Bi2 atom in **2** has relatively short distances to two aromatic six-membered rings of the two fluorenyl ligands. This could potentially be a cation - π interaction, which also contributes to the further stabilization of the moiety. Overall, the allylic Bi-Bi-Bi unit in the center of the cation seems to be sterically well shielded by the two bulky fluorenyl ligands surrounding it.

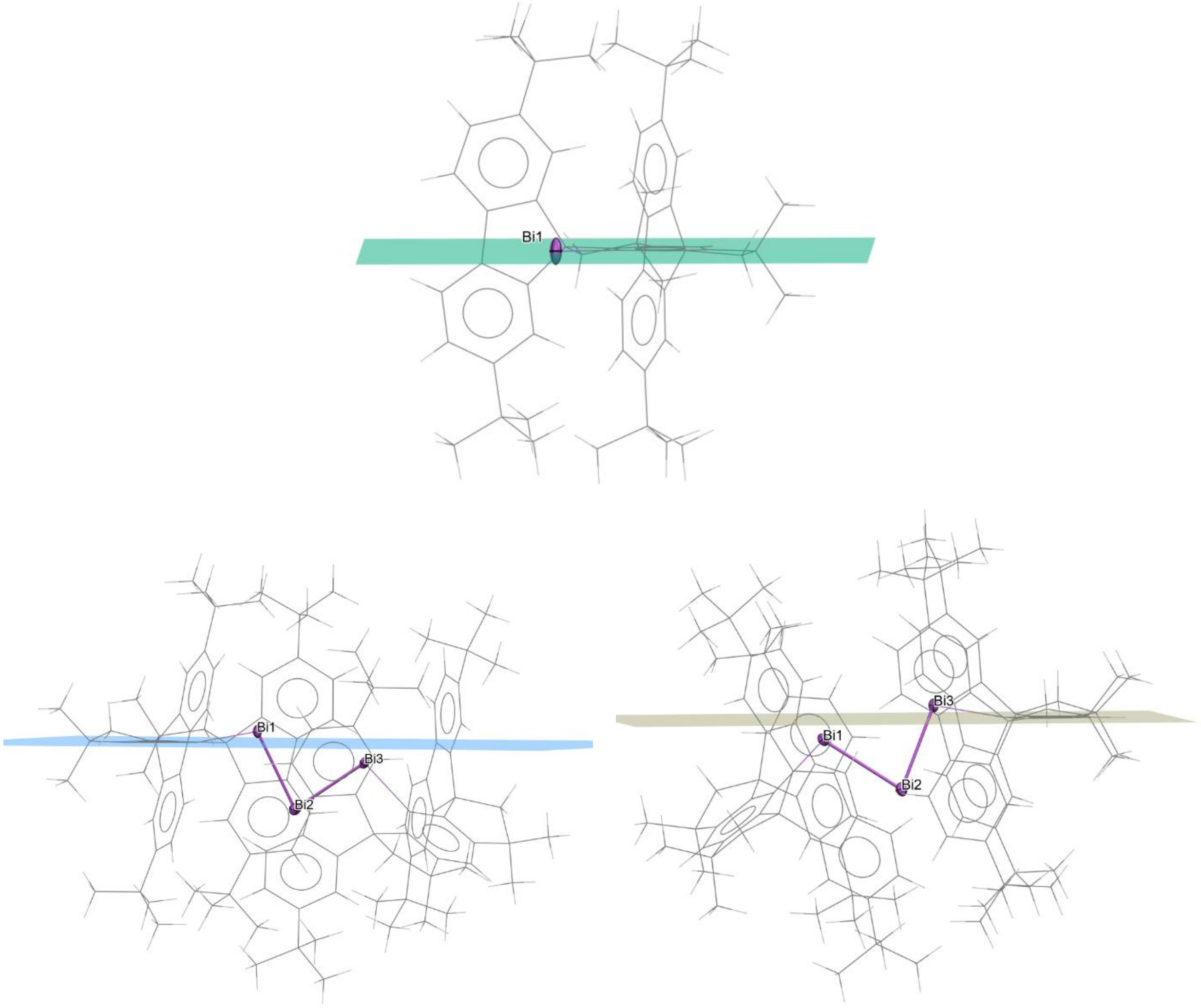

Supplementary Figure S68. Comparison between the position of the Bi-atoms in Bi(I) **1** structure (top; GEZGUR) and **2** (bottom). The coloured planes are placed through the plane of the aromatic compound bonded to the Bi-atom.

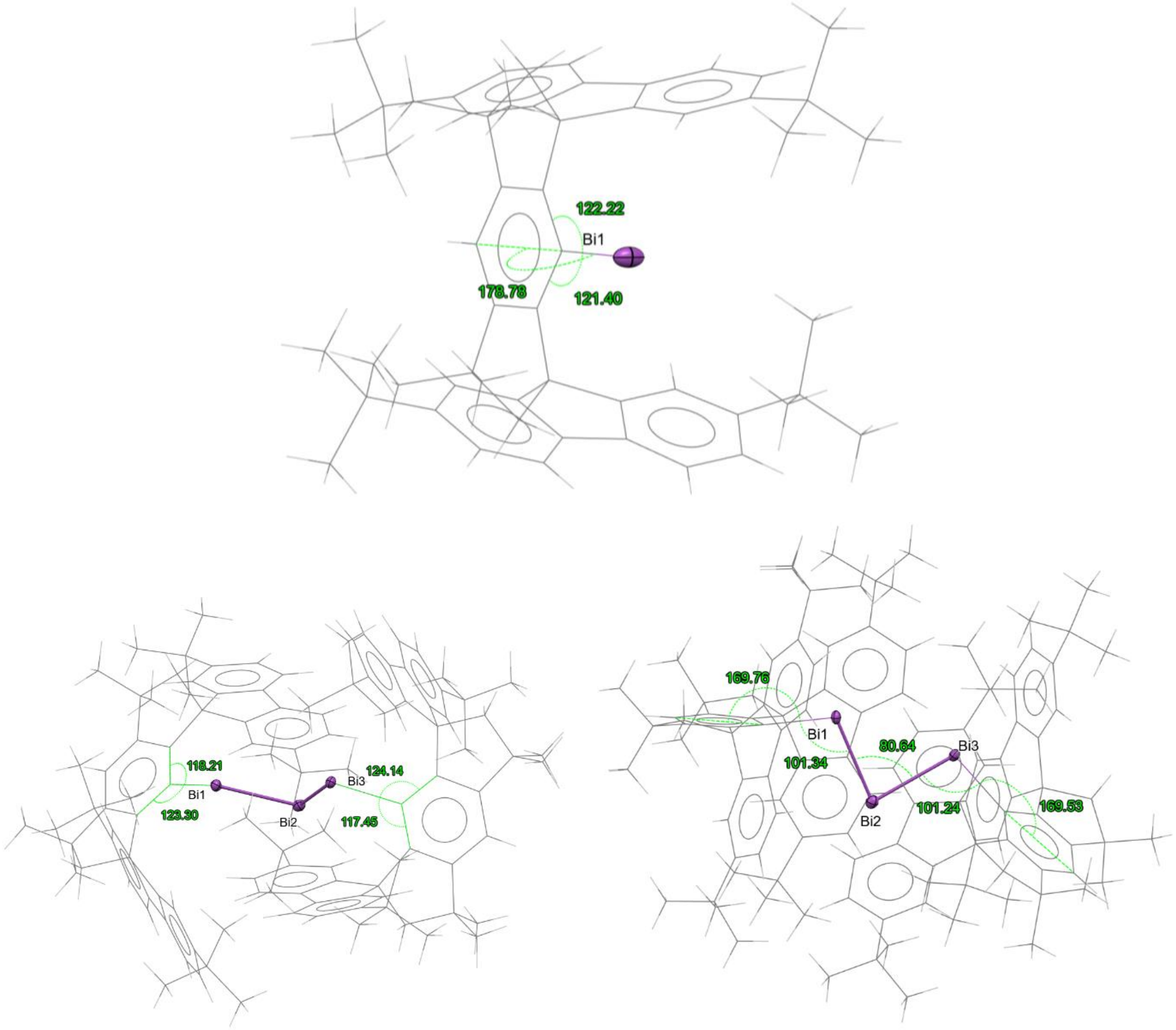

Supplementary Figure S69. Comparison of different bond angles and lengths between Bi(I) **1** (top; GEZGUR) and structure **2** (bottom).

Another difference between structure **2** and **1** is the distortion of the Bi-C unit. **1** has an expected geometry and has two C-C-Bi angles of almost 120° (122.22 and 121.40°). The central Bi1 atom is centered on the plane of the aromatic ring bonded to it (C_{ipso}-C-Bi angle of 178.78°). The situation is slightly different in structure **2**, where the two Bi1 and Bi3 atoms are shifted out of the plane of the attached aromatic rings. This results in slightly different C-C-Bi angles (118.21 and 123.30° for Bi1, 124.14 and 117.45° for Bi3). The two C_{ipso}-C-Bi angles here are 169.76 and 169.53°. It is noteworthy that the Bi-C distance in the Bi(I) **1** with 2.2783(10) Å is not so different from the two Bi-C distances with 2.2833(16) and 2.2869(16) Å in **2**.

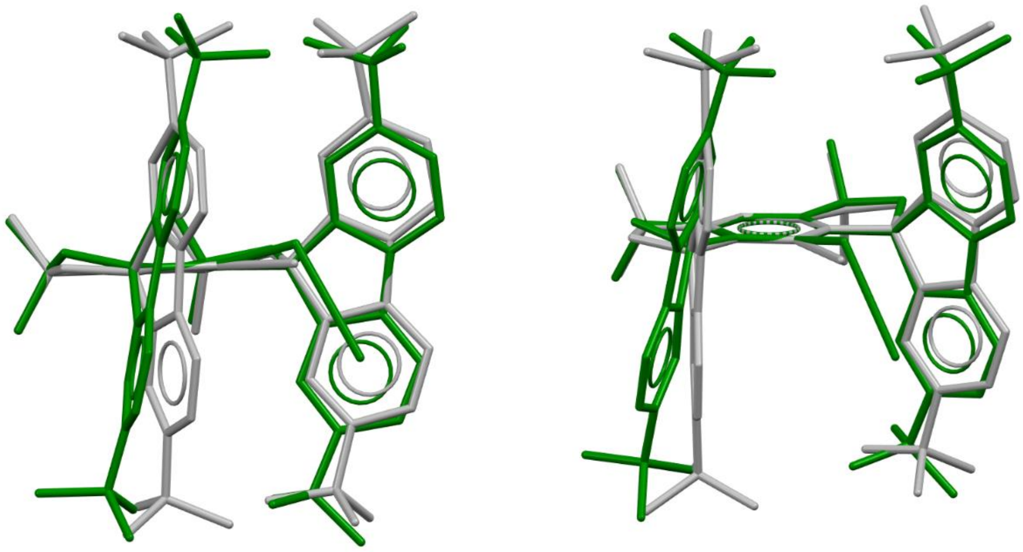

Supplementary Figure S70. Overlay of the two Bi-fluorenyl fragments Bi1 and Bi3 of **2** (green) with the structure of Bi(I) **1** (grey).

The geometric differences become particularly apparent when the respective Bi-fluorenyl fragments are super positioned with the Bi(I) **1**. The six carbon atoms of the central aromatic ring were used as reference points for the super positioning. An RMS of 0.0118 is observed for the superposition of the Bi1 fragment with the structure in **1**. An even greater difference is observed when the Bi3 fragment is super positioned, an RMS of 0.0157 is observed.

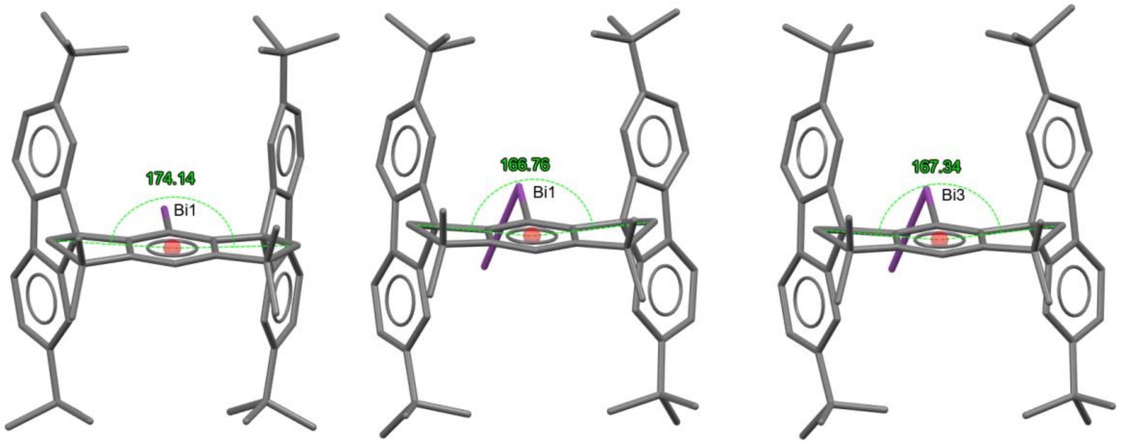

Supplementary Figure S71. Illustration of the distortion of the fluorenyl backbone of the Bi(I) **1** (left) and the two sub units in structure of **2** (centre and right).

Another structural difference between the **2** and **1** is the bending of the fluorene backbone in the plane of the Bi atoms. In **1**, this fragment is almost flat, with a C-centroid-C angle of 174.14°. The situation is different for the two fluorene backbones in **2**, where C-centroid-C angles of 166.76° and 167.34° are observed. This significant distortion indicates a certain flexibility of the backbone structure, which allows the three central Bi atoms in **2** to interact with each other. The distorted geometry is due to the steric

requirements of the two fluorenyl ligands, but also to the large space requirement of the allylic unit Bi-Bi-Bi.

4.4. Single crystal structure analysis of **2’**

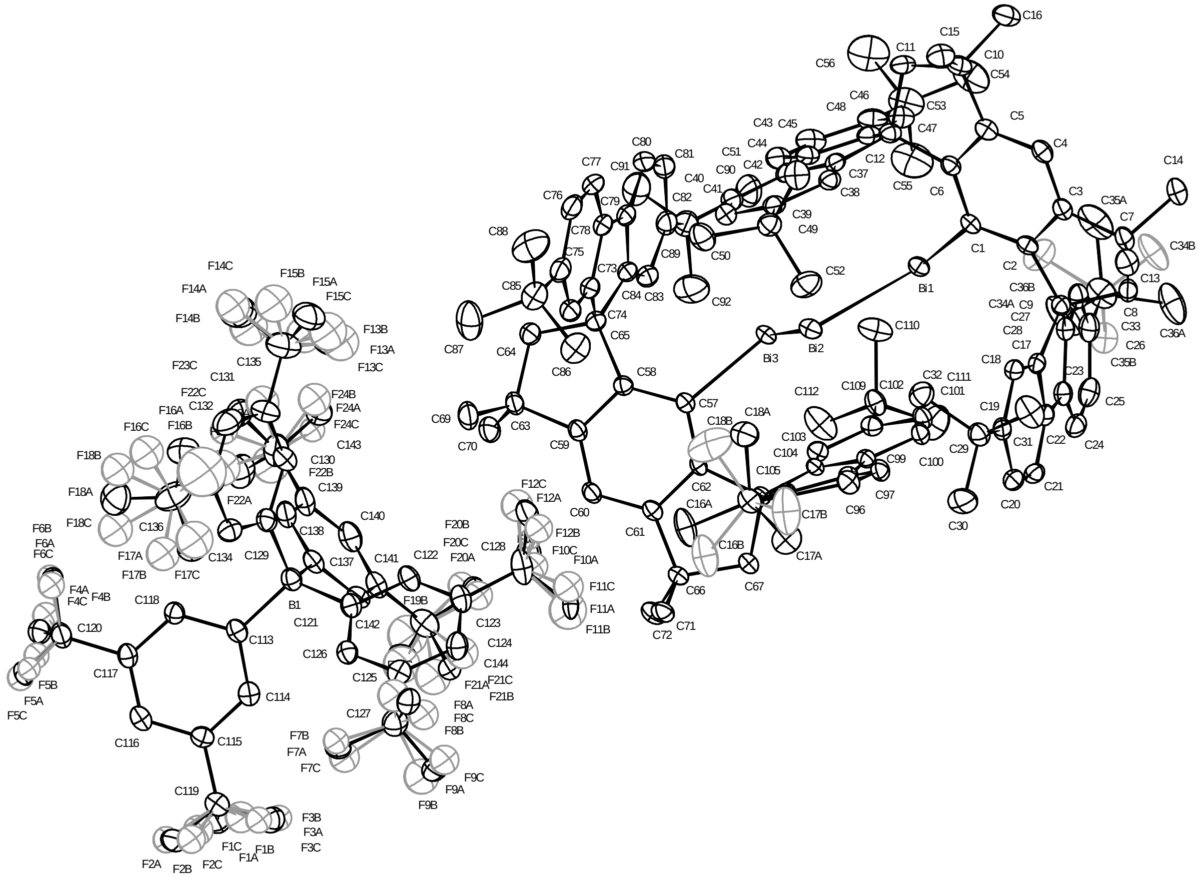

Supplementary Figure S72. ORTEPfor structure **2’**, thermal ellipsoid at 50% probability level. H atoms have been removed for clarity.

X-ray Crystal Structure Analysis of complex **2’**:

$C_{144}H_{142}BBi_3F_{24}$, M_r = 2966.27 g mol^{-1}, brown-red prism, crystal size 0.205 x 0.121 x 0.041 mm^3, monoclinic, space group $P2_1/c$ [14], a = 11.6467(5) Å, b = 48.3956(19) Å, c = 23.2244(8) Å, β = 93.310(2)°, V = 13068.6(9) Å^3, T = 100(2) K, Z = 4, D_{calc} = 1.508 g·cm^3, λ = 0.71073 Å, $\mu(Mo\text{-}K_\alpha)$ = 4.113 mm^{-1}, Numerical correction ($T_{\min}$ = 0.6003, $T_{\max}$ = 0.9140), Bruker-AXS D8 Venture with Photon III detector and IµS Diamond microfocus Mo-anode X-ray source, 1.899 < θ < 30.034°, 1016108 measured reflections, 38252 independent reflections, 30178 reflections with $I > 2\sigma(I)$, R_{int} = 0.1243. The structure was solved by *SHELXT* and refined by full-matrix least-squares (*SHELXL*) against F^2 to R_1 = 0.0382 [$I > 2\sigma(I)$], wR_2 = 0.0862 [all data], 1836 parameters and 331 restraints.[5-8]

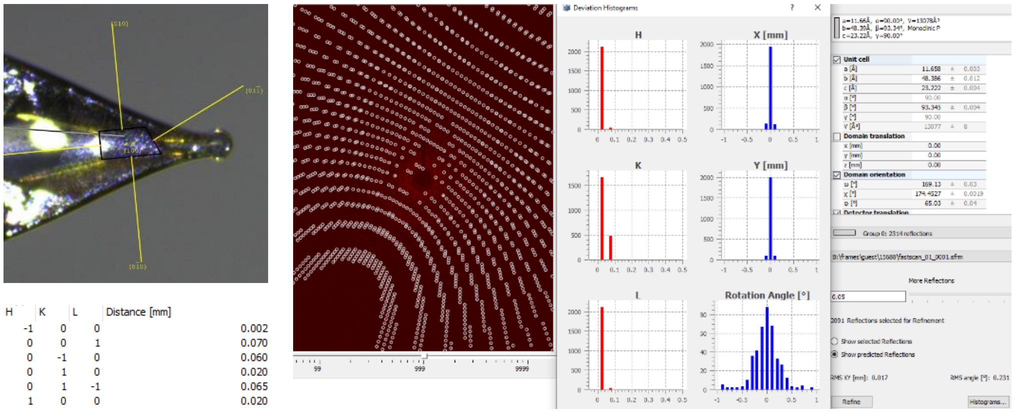

Supplementary Figure S73. Crystal faces and unit cell determination/refinement of complex **2'**.

INTENSITY STATISTICS FOR DATASET

Resolution	#Data	#Theory	%Complete	Redundancy	Mean I	Mean I/s	Rmerge	Rsigma
Inf - 2.85	611	622	98.2	50.58	99.66	83.47	0.0325	0.0105
2.85 - 1.90	1439	1439	100.0	43.78	70.80	65.00	0.0410	0.0108
1.90 - 1.51	2029	2029	100.0	34.55	45.50	48.36	0.0574	0.0144
1.51 - 1.32	2002	2002	100.0	32.04	35.35	38.85	0.0729	0.0174
1.32 - 1.19	2207	2207	100.0	29.44	30.57	33.19	0.0871	0.0206
1.19 - 1.11	1913	1913	100.0	29.19	23.82	27.77	0.1097	0.0249
1.11 - 1.04	2189	2189	100.0	28.71	19.53	23.88	0.1305	0.0295
1.04 - 0.99	1945	1945	100.0	28.39	16.35	20.77	0.1540	0.0349
0.99 - 0.95	1897	1897	100.0	26.56	16.76	19.70	0.1576	0.0371
0.95 - 0.91	2192	2192	100.0	25.71	14.93	17.56	0.1771	0.0430
0.91 - 0.88	1945	1945	100.0	24.28	13.18	14.95	0.1967	0.0508
0.88 - 0.85	2232	2232	100.0	23.54	11.13	12.51	0.2266	0.0609
0.85 - 0.82	2561	2561	100.0	23.47	9.66	10.77	0.2573	0.0717
0.82 - 0.80	1921	1921	100.0	23.14	8.18	9.12	0.3021	0.0870
0.80 - 0.78	2096	2096	100.0	22.39	7.67	8.23	0.3195	0.0968
0.78 - 0.76	2392	2392	100.0	22.02	7.24	7.56	0.3467	0.1074
0.76 - 0.75	1290	1290	100.0	21.64	6.83	7.04	0.3683	0.1174
0.75 - 0.73	2713	2713	100.0	20.98	6.26	6.31	0.3962	0.1330
0.73 - 0.72	1515	1515	100.0	20.53	5.90	5.72	0.4340	0.1481
0.72 - 0.71	1622	1622	100.0	17.74	5.51	4.92	0.4520	0.1741
0.71 - 0.70	1774	1868	95.0	15.14	5.00	4.19	0.4824	0.2186
0.80 - 0.70	13402	13496	99.3	20.20	6.41	6.39	0.3844	0.1341
Inf - 0.70	40485	40590	99.7	25.90	18.65	19.84	0.1166	0.0388

Complete .cif-data are available under the CCDC number CCDC**- 2368301**.

Alert A: PLAT307_ALERT_2_A Isolated Metal Atom found in Structure (Unusual) Bi2 Check.
Author response: it was the scope of this research project to obtain such an unusual bond situation. Please see manuscript and SI for further information.

Alert A: PLAT308_ALERT_2_A Single Bonded Metal Atom in Structure (Unusual) Bi1 Check.

Author response: it was the scope of this research project to obtain such an unusual bond situation. Please see manuscript and SI for further information.
Alert A: PLAT308_ALERT_2_A Single Bonded Metal Atom in Structure (Unusual) Bi3 Check.
Author response: it was the scope of this research project to obtain such an unusual bond situation. Please see manuscript and SI for further information.

Alert B: PLAT971_ALERT_2_B Check Calcd Resid. Dens. 1.62Ang From Bi2 2.68 eA^{-3}.
Author Response: This is probably due to some trace impurities. Bi has a remarkably high electron number, trace amounts of other species or crystal domains can cause such results. The structure is confirmed by several other analytical techniques and there is no evidence for twinning or misaligned symmetry. In addition, anharmonic motions are probably responsible for the high residual electron density around Bi1 and Bi3.

A result cut-off (SHEL 999 0.71) was applied to exclude incomplete shells at high diffraction angles. Prior to the final refinement cycles, some reflexes showed a high I/σI and were omitted from the data set. The occupancy of the disordered areas were refined with free variables.
In addition, restrains were used to improve the geometries of the disordered regions. The high residual electron density is probably due to some trace impurities. Bi has a remarkably high electron number, trace amounts of other species or crystal domains can cause such results. The structure is confirmed by several other analytical techniques and there is no evidence for twinning or misaligned symmetry. In addition, the high residual electron density around Bi1 and Bi3 is likely to be caused by caused by anharmonic motions.[5-8]

Supplementary Table S5. Crystal data and structure refinement of **2'**.

Identification code	15688sadabs	
Empirical formula	$C_{144} H_{142} B Bi_3 F_{24}$	
Color	brown-red	
Formula weight	2966.27 $g \cdot mol^{-1}$	
Temperature	100(2) K	
Wavelength	0.71073 Å	
Crystal system	Monoclinic	
Space group	$P2_1/c$, (no. 14)	
Unit cell dimensions	a = 11.6467(5) Å	α= 90°.
	b = 48.3956(19) Å	β= 93.310(2)°.
	c = 23.2244(8) Å	γ = 90°.
Volume	13068.6(9) $Å^3$	

Z	4	
Density (calculated)	1.508 $Mg{\cdot}m^{-3}$	
Absorption coefficient	4.113 mm^{-1}	
F(000)	5904 e	
Crystal size	0.205 x 0.121 x 0.041 mm^3	
θ range for data collection	1.899 to 30.034°.	
Index ranges	$-16 \le h \le 16$, $-68 \le k \le 68$, $-32 \le l \le 32$	
Reflections collected	1016108	
Independent reflections	38252 [R_{int} = 0.1243]	
Reflections with I>2σ(I)	30178	
Completeness to θ = 25.242°	99.9 %	
Absorption correction	Numerical	
Max. and min. transmission	0.9140 and 0.6003	
Refinement method	Full-matrix least-squares on F^2	
Data / restraints / parameters	38252 / 331 / 1836	
Goodness-of-fit on F^2	1.072	
Final R indices [I>2σ(I)]	R_1 = 0.0382	wR^2 = 0.0789
R indices (all data)	R_1 = 0.0583	wR^2 = 0.0862
Extinction coefficient	n/a	
Largest diff. peak and hole	2.584 and -1.080 $e{\cdot}Å^{-3}$	

4.5. Single crystal structure analysis of **8**

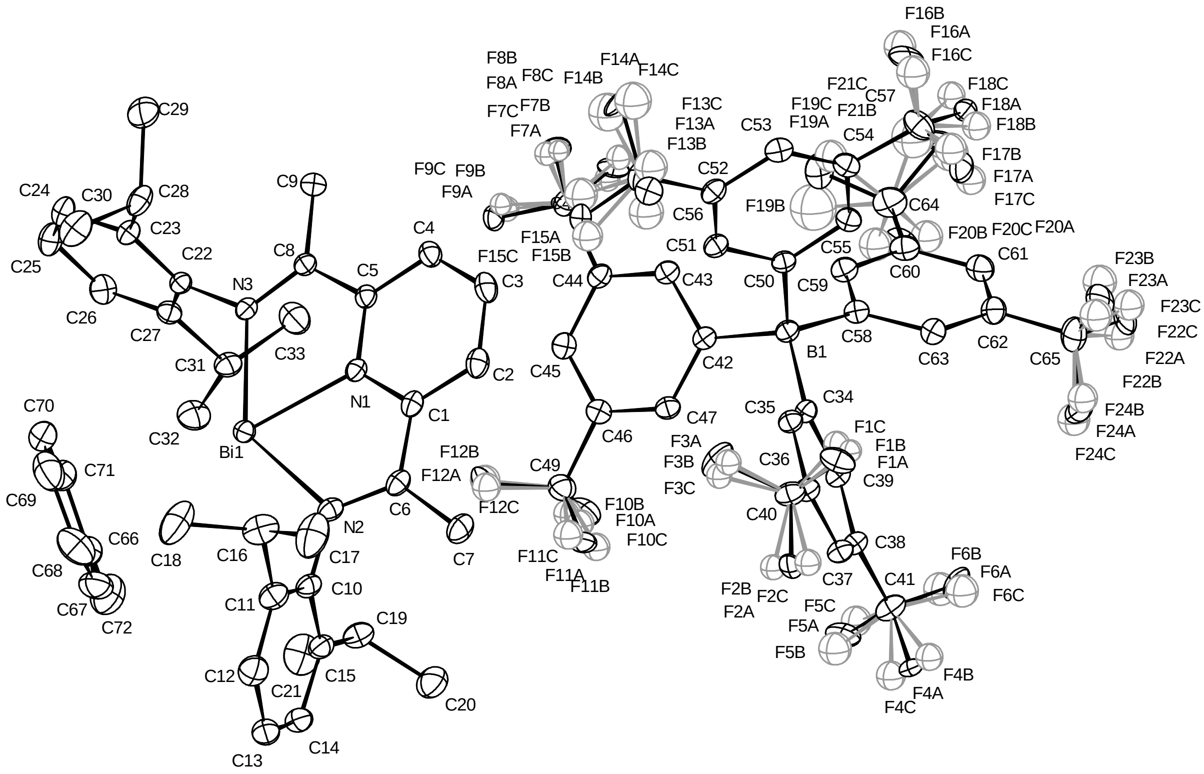

Supplementary Figure S74. ORTEP for structure **8**; thermal ellipsoid at 50% probability level. H atoms have been removed for clarity, disordered parts shown in grey.

X-ray Crystal Structure Analysis of complex **8**:

C_{72} H_{63} B Bi F_{24} N_3, M_r = 1646.14 g mol^{-1}, blue prism, crystal size 0.402 x 0.188 x 0.18 mm^3, triclinic, space group *P*-1 [2], *a* = 12.4476(5) Å, *b* = 16.0366(7) Å, *c* = 17.8158(7) Å, α = 94.051(2)°, β = 98.636(2)°, γ = 96.952(2)°, *V* = 3475.8(2) Å^3, *T* = 100(2) K, *Z* = 2, D_{calc} = 1.573 g·cm^3, λ = 0.71073 Å, $\mu(Mo\text{-}K_\alpha)$ = 3.793 mm^{-1}, Numerical correction ($T_{\min}$ = 0.4348, $T_{\max}$ = 1.0000), Bruker-AXS D8 Venture with Photon III detector and IµS Diamond microfocus Mo-anode X-ray source, 2.322 < θ < 31.544°, 788631 measured reflections, 23178 independent reflections, 21757 reflections with $I > 2\sigma(I)$, R_{int} = 0.0777. The structure was solved by *SHELXT* and refined by full-matrix least-squares (*SHELXL*) against F^2 to R_1 = 0.0193 [$I > 2\sigma(I)$], wR_2 = 0.0509 [all data], 1116 parameters and 289 restraints.[5-8]

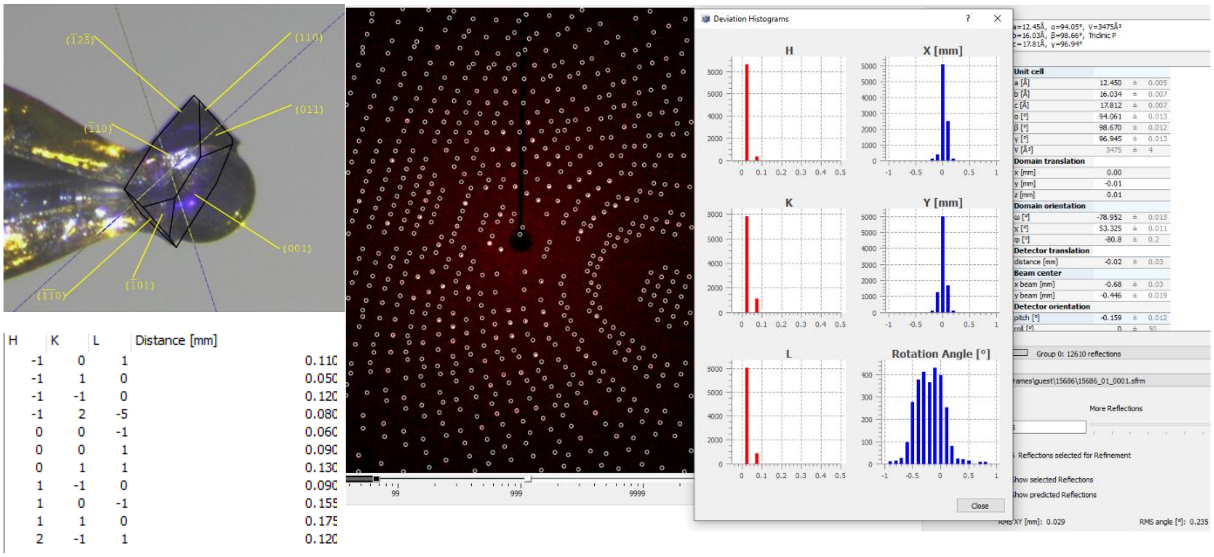

Supplementary Figure S75. Crystal faces and unit cell determination/refinement of complex **8**.

INTENSITY STATISTICS FOR DATASET

Resolution	#Data	#Theory	%Complete	Redundancy	Mean I	Mean I/s	Rmerge	Rsigma
Inf - 2.66	707	776	91.1	16.08	79.23	64.52	0.0381	0.0469
2.66 - 1.81	1639	1678	97.7	17.83	54.28	68.31	0.0369	0.0236
1.81 - 1.45	2306	2312	99.7	19.49	40.03	66.57	0.0391	0.0136
1.45 - 1.27	2318	2320	99.9	19.17	29.08	58.41	0.0455	0.0138
1.27 - 1.15	2494	2494	100.0	18.86	24.22	51.75	0.0528	0.0156
1.15 - 1.07	2308	2308	100.0	18.38	21.32	46.03	0.0579	0.0172
1.07 - 1.01	2234	2234	100.0	18.17	18.06	40.97	0.0660	0.0195
1.01 - 0.96	2298	2298	100.0	17.89	16.15	37.54	0.0741	0.0215
0.96 - 0.92	2286	2286	100.0	17.69	13.62	33.40	0.0852	0.0244
0.92 - 0.88	2678	2678	100.0	17.53	11.36	28.98	0.0978	0.0280
0.88 - 0.85	2334	2334	100.0	17.27	10.42	26.87	0.1075	0.0309
0.85 - 0.82	2712	2712	100.0	16.95	8.99	23.33	0.1187	0.0353
0.82 - 0.80	1996	1996	100.0	16.13	8.54	21.50	0.1268	0.0387
0.80 - 0.78	2244	2244	100.0	16.04	8.04	20.07	0.1318	0.0414
0.78 - 0.76	2504	2504	100.0	16.04	7.15	18.33	0.1435	0.0456
0.76 - 0.74	2742	2742	100.0	16.04	6.60	16.89	0.1495	0.0492
0.74 - 0.73	1470	1470	100.0	15.79	6.36	16.26	0.1538	0.0524
0.73 - 0.71	3296	3296	100.0	15.73	5.59	14.36	0.1645	0.0583
0.71 - 0.70	1756	1756	100.0	15.53	5.11	13.21	0.1710	0.0639
0.70 - 0.69	1864	1864	100.0	15.43	5.08	12.92	0.1756	0.0659
0.69 - 0.68	2044	2178	93.8	13.14	4.49	11.00	0.1849	0.0848
0.78 - 0.68	15676	15810	99.2	15.42	5.83	14.88	0.1598	0.0574
Inf - 0.68	46230	46480	99.5	16.97	15.76	31.60	0.0758	0.0277

Complete .cif-data are available under the CCDC number CCDC-**2368300**.
Some reflexes showed a high I/σI and were omitted from the data set before the final refinement cycles.

Supplementary Table S6. Crystal data and structure refinement of **8**.

Identification code	15686	
Empirical formula	$C_{72} H_{63} B Bi F_{24} N_3$	
Color	blue	
Formula weight	1646.14 g·mol^{-1}	
Temperature	100.00 K	
Wavelength	0.71073 Å	
Crystal system	Triclinic	
Space group	*P*-1, (no. 2)	
Unit cell dimensions	a = 12.4476(5) Å	α= 94.051(2)°.
	b = 16.0366(7) Å	β= 98.636(2)°.
	c = 17.8158(7) Å	γ = 96.952(2)°.
Volume	3475.8(2) Å^3	
Z	2	
Density (calculated)	1.573 Mg·m^{-3}	
Absorption coefficient	2.645 mm^{-1}	
F(000)	1640 e	
Crystal size	0.402 x 0.188 x 0.18 mm^3	
θ range for data collection	2.322 to 31.544°.	
Index ranges	$-18 \le h \le 18, -23 \le k \le 23, -26 \le l \le 26$	
Reflections collected	788631	
Independent reflections	23178 [R_{int} = 0.0777]	
Reflections with I>2σ(I)	21757	
Completeness to θ = 25.242°	99.8 %	
Absorption correction	Numerical	
Max. and min. transmission	1.0000 and 0.4348	
Refinement method	Full-matrix least-squares on F^2	
Data / restraints / parameters	23178 / 289 / 1116	
Goodness-of-fit on F^2	1.052	
Final R indices [I>2σ(I)]	R_1 = 0.0193	wR^2 = 0.0501
R indices (all data)	R_1 = 0.0216	wR^2 = 0.0509
Extinction coefficient	n/a	
Largest diff. peak and hole	0.814 and -1.276 e·Å^{-3}	

5. Computational Details

5.1. Computational methods

All calculations were carried out with a development version of ORCA based on ORCA 5.0.[9,10] Geometries were optimized without constraints using the B3LYP functional[11,12] and the D3BJ dispersion correction[13] the def2-TZVP basis set[14] and associated effective core potentials.

For the TD-DFT calculations we used the X2C scalar relativistic Hamiltonian together with a finite nucleus representation (the generic acronym "X2C" for exact two-component Hamiltonians resulted from intensive discussions among H. J. Aa. Jensen, W. Kutzelnigg, W. Liu, T. Saue and L. Visscher during the Twelfth International Conference on the Applications of Density Functional Theory DFT-2007, Amsterdam, 26-30 August 2007)[15] as implemented into a development version of ORCA[16] (unpublished work of G. Stoychev, C. Riplinger, F. Neese) and the X2C-SVPall (C,H) and X2C-TZVPall (Bi) basis set.[17,18] Spin-orbit coupling (SOC) was treated using the mean-field SOC Hamiltonian[19,20] including picture change effects. The SOC was incorporated by quasi-degenerate perturbation theory (QDPT) by diagonalizing the SOC operator over the lowest 20 singlet and triplet roots. The resulting scalar relativistic or SOC containing transition intensities were computed in the dipole length formalism and the resulting stick spectrum was convoluted with Gaussian bands using a uniform bandwidth of 1500 cm^{-1}.

5.2. Calculated absorption of **2**

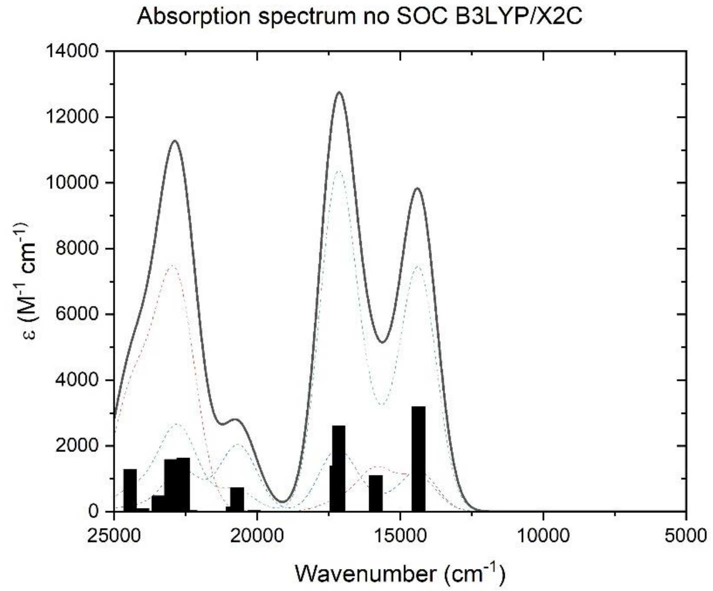

Supplementary Figure S76. Calculated absoprtion spectra of **2** without Spin Orbital Coupling evaluation.

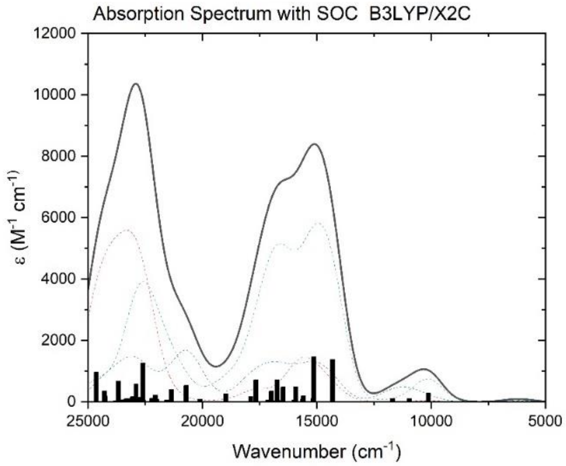

Supplementary Figure S77. Calculated absoprtion spectra of **2** with Spin Orbital Coupling included.

5.3. Comparison of different functionals

For this comparison the linewidth of the Gaussian bands used to convolute the spectral traces has been set to 1000 cm^{-1} in order to allow for a more detailed comparison.

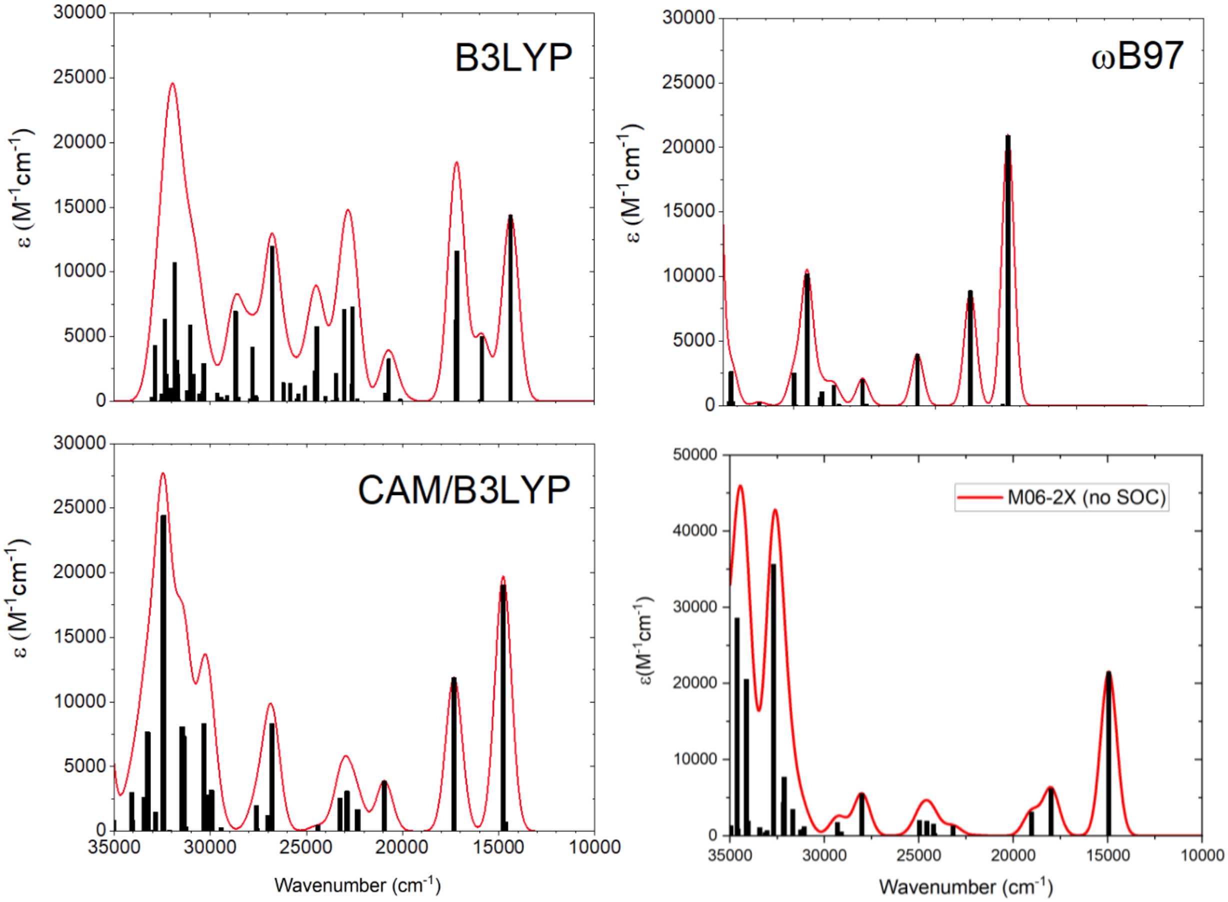

Supplementary Figure S78. Calculated absoprtion spectra of **2** without SOC and with different density functionals.

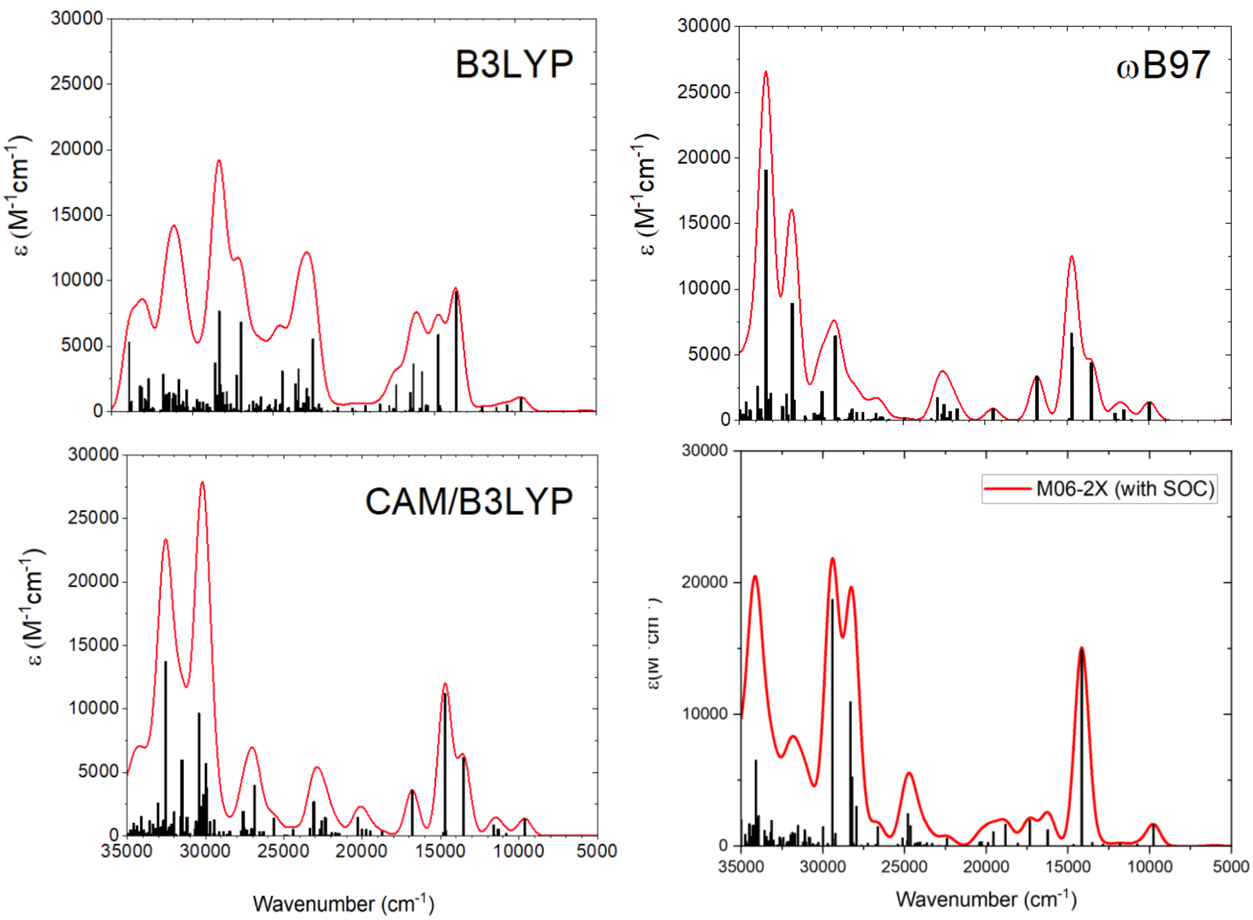

Supplementary Figure S79. Calculated absoprtion spectra of **2** with Spin Orbit Coupling and with different density functionals.

Despite obvious visible differences in the shape of the calculated spectra, the results obtained with different funcitonals are qualitatively consistent. They all predict the singlet-triplet dominated electronic transitions around 10000 cm^{-1} that gain intensity through spin-orbit coupling. Three main transitions in the area around 150000 cm^{-1} carry the main intensity in the red region of the spectrum with a number of minor transitions borrowing intensity from them. These transitions have been discusssed in the main text. The spectrum than drops in intensity and the density of states is significantly increasing before high metal-to-ligand intensity picks up towards the blue region of the spectrum. The resolution of the experimental spectrum does not permit a more detailed assignment of the observed spectral features. Among the different density functionals none is in strikingly better agreement with the experimental absorption envelope than the other tested ones. Possibly wB97, a range separated functional, is slightly preferred as it provides an overall intensity distribution that appears to match the experimental envelope slightly better. However, we emphasize that there are no significant qualitative differences in the calculated spectra.

5.4. Calculated orbitals for the C-Bi_3^+-C core

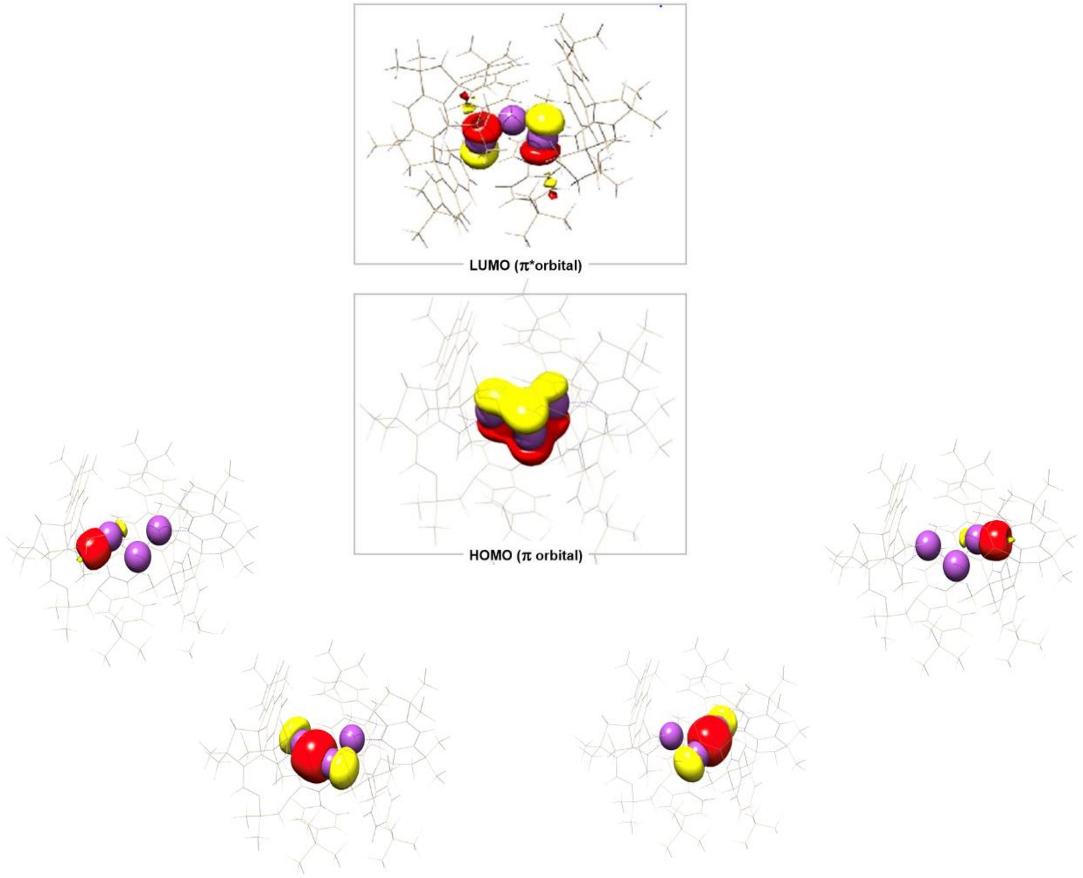

Supplementary Figure S80. Calculated orabitals for the C-Bi_3^+-C core. From top to the bottom: LUMO (π* orbital), HOMO (π orbital), Bi-C (σ bond), and Bi-Bi (σ bond).

6. References

[1] Pang, Y. *et al.* Synthesis and isolation of a triplet bismuthinidene with a quenched magnetic response. *Science* **380**, 1043-1048 (2023).

[2] Lesnichin, S. B. *et al.* Intrinsic Proton-Donating Power of Zinc-Bound Water in a Carbonic Anhydrase Active Site Model Estimated by NMR. *J. Am. Chem. Soc.* **133**, 11331–11338 (2011).

[3] Harris, K. R. *et al.* FURTHER CONVENTIONS FOR NMR SHIELDING AND CHEMICAL SHIFTS. *Pure Appl. Chem.* **80**, 59–84 (2008).

[4] Mato, M. *et al.* Bismuth radical catalysis in the activation and coupling of redox-active electrophiles. *Nat. Chem.* **15**, 1138–1145 (2023).

[5] Kratzert, D. & Krossing, I. Recent improvements in DSR. *J. Appl. Cryst.* **51**, 928–934 (2018).

[6] Kratzert, D., Holstein, J.J., & Krossing, I. DSR: enhanced modelling and refinement of disordered structures with SHELXL. *J. Appl. Cryst*. **48**, 933–938 (2015).

[7] Dolomanov, O. V., Bourhis, L. J., Gildea, R. J., Howard J. A. K., Puschmann, H. OLEX2: a complete structure solution, refinement and analysis program. *J. Appl. Cryst.* **42**, 339–341 (2009).

[8] Sheldrick, G.M. Crystal structure refinement with SHELXL. *Acta Cryst*. C**71**, 3–8 (2015).

[9] Neese, F., Wennmohs, F., Becker, U. & Riplinger, C. The ORCA quantum chemistry program package. *J. Chem. Phys.* **152**, 224108 (2020).

[10] Neese, F. Software update: The ORCA program system—Version 5.0. *Wiley Interdiscip. Rev. Comput. Mol. Sci.* **12**, e1606 (2022).

[11] Van Wüllen, C. Molecular density functional calculations in the regular relativistic approximation: Method, application to coinage metal diatomics, hydrides, fluorides and chlorides, and comparison with first-order relativistic calculations. *J. Chem. Phys.* **109**, 392–399 (1998).

[12] Becke, A. D. A new mixing of Hartree–Fock and local density-functional theories. *J. Chem. Phys.* **98**, 1372–1377 (1993).

[13] Pantazis, D. A & Neese, F. All-electron scalar relativistic basis sets for the 6p elements. *Theor. Chem. Acc*. **131**, 1292 (2012).

[14] Weigend, F. & Ahlrichs, R. Balanced basis sets of split valence, triple zeta valence and quadruple zeta valence quality for H to Rn: Design and assessment of accuracy. *Phys. Chem. Chem. Phys.* **7**, 3297–3305 (2005).

[15] Angeli, C., Cimiraglia, R., Evangelisti, S., Leininger, T. & Malrieu, J. P. Introduction of n-electron valence states for multireference perturbation theory. *J. Chem. Phys.* **114**, 10252–10264 (2001).

[16] Kutzelnigg, W. & Liu, W. Quasirelativistic theory equivalent to fully relativistic theory. *J. Chem. Phys*. **123**, 241102 (2005).

[17] Pollak, P. & Weigend, F. Segmented contracted error-consistent basis sets of double- and triple-ζ valence quality for one- and two-component relativistic all-electron calculations. *J. Chem. Theory Comput.* **13**, 3696–3705 (2017).

[18] Franzke, Y. J., Treß, R., Pazdera, T. M. & Weigend, F. Error-consistent segmented contracted all-electron relativistic basis sets of double- and triple-zeta quality for NMR shielding constants. *Phys. Chem. Chem. Phys.* **21**, 16658–16664 (2019).

[19] Heß, B. A., Marian, C. M., Wahlgren, U. & Gropen, O. A mean-field spin-orbit method applicable tocorrelated wavefunctions. *Chem. Phys. Lett.* **251**, 365–371 (1996).

[20] Neese, F. Efficient and accurate approximations to the molecular spin-orbit coupling operator and their use in molecular g-tensor calculations. *J. Chem. Phys*. **122**, 34107 (2005).
